# Supplementary material for: Reporting of outcomes in gastric cancer surgery trials: a systematic review
Source: BMJ Open. 2018 Oct 17;8(10):e021796. doi: 10.1136/bmjopen-2018-021796 (PMC6196805; doi:10.1136/bmjopen-2018-021796)
Supplement: Supplementary file 1 [file bmjopen-2018-021796supp001.pdf]

Appendix 1. Search Algorithm

Database: Ovid MEDLINE (R)
Search Strategy:
----
1 (Gastr$ or stomach$ or oesophagogastric junction or esophagogastric junction).mp. (645201)
2 exp Stomach/ (112711)
3 adenocarcinoma/ or neoplasms/ (457904)
4 1 or 2 (648680)
5 3 and 4 (37749)
6 exp Stomach Neoplasms/ (81208)
7 5 or 6 (100682)
8 chemoradiotherapy/ or chemotherapy, adjuvant/ or consolidation chemotherapy/ or antineoplastic combined chemotherapy protocols/ or induction chemotherapy/ (140865)
9 (antineoplast$ or antitumor$ or anti-tumor$ or anti-neoplast$ or chemotherp$).mp. (454727)
10 exp Gastrectomy/ or endoscopy, gastrointestinal/ or duodenoscopy/ or esophagoscopy/ or gastroscopy/ (69973)
11 (gastrectom$ or duodenoscop$ or esophagoscop$ or oesophagoscop$ or gastroscop$).mp. [mp=title, abstract, original title, name of substance word, subject heading word, keyword heading word, protocol supplementary concept word, rare disease supplementary concept word, unique identifier] (66259)
12 radiotherapy/ or brachytherapy/ or radioimmunotherapy/ or radiotherapy, adjuvant/ (74386)
13 (radiotherap$ or chemoradiotherap$ or chemo-radiotherap$ or "radiation therap$" or bracytherap$ or irradiat$).mp. (366760)
14 8 or 9 or 10 or 12 or 13 (854226)
15 7 and 14 (32194)
16 randomi?ed controlled trial.pt. (416592)
17 controlled clinical trial.pt. (92207)
18 randomized.ab. (308489)
19 randomly.ab. (218498)
20 exp animals/ not humans.sh. (4150916)
21 16 or 17 or 18 or 19 (743790)
22 21 not 20 (673762)
23 15 and 22 (2255)
24 limit 23 to yr="1996 -Current" (1806)
25 (Gastr$ or stomach$ or oesophagogastric junction or esophagogastric junction).mp. (645201)
26 exp Stomach/ (112711)
27 adenocarcinoma/ or neoplasms/ (457904)
28 25 or 26 (648680)
29 27 and 28 (37749)
30 exp Stomach Neoplasms/ (81208)
31 29 or 30 (100682)
32 chemoradiotherapy/ or chemotherapy, adjuvant/ or consolidation chemotherapy/ or antineoplastic combined chemotherapy protocols/ or induction chemotherapy/ (140865)
33 (antineoplast$ or antitumor$ or anti-tumor$ or anti-neoplast$ or chemotherp$).mp. (454727)
34 exp Gastrectomy/ or endoscopy, gastrointestinal/ or duodenoscopy/ or esophagoscopy/ or gastroscopy/ (69973)
35 (gastrectom$ or duodenoscop$ or esophagoscop$ or oesophagoscop$ or gastroscop$).mp. [mp=title, abstract, original title, name of substance word, subject heading word, keyword heading word, protocol supplementary concept word, rare disease supplementary concept word, unique identifier] (66259)
36 radiotherapy/ or brachytherapy/ or radioimmunotherapy/ or radiotherapy, adjuvant/ (74386)
37 (radiotherap$ or chemoradiotherap$ or chemo-radiotherap$ or "radiation therap$" or bracytherap$ or irradiat$).mp. (366760)
38 32 or 33 or 34 or 36 or 37 (854226)
39 31 and 38 (32194)
40 randomi?ed controlled trial.pt. (416592)
41 controlled clinical trial.pt. (92207)

42 randomized.ab. (308489)
43 randomly.ab. (218498)
44 exp animals/ not humans.sh. (4150916)
45 40 or 41 or 42 or 43 (743790)
46 45 not 44 (673762)
47 39 and 46 (2255)
48 limit 47 to yr="1996 -Current" (1806)
