# Supplementary material for: Reporting of outcomes in gastric cancer surgery trials: a systematic review
Source: BMJ Open. 2018 Oct 17;8(10):e021796. doi: 10.1136/bmjopen-2018-021796 (PMC6196805; doi:10.1136/bmjopen-2018-021796)
Supplement: Supplementary file 2 [file bmjopen-2018-021796supp002.pdf]

Appendix 2. Outcomes reported in 48 publications from 32 gastric cancer surgery trials.

Theme	Original Outcome	Frequency Reported
Adverse Events	Abdominal abscess	10
Adverse Events	Abdominal distention	1
Adverse Events	Abdominal drainage	1
Adverse Events	Abdominal liquid accumulation	1
Adverse Events	Abscess intra-abdominal	7
Adverse Events	Abscess subphrenic	1
Adverse Events	Acute enteritis	1
Adverse Events	Acute urinary retention	1
Adverse Events	Adverse drug reaction	1
Adverse Events	Afferent loop syndrome	2
Adverse Events	Allogenic blood transfusion	1
Adverse Events	Amylase level in drainage fluid	1
Adverse Events	Anastomosis failure	1
Adverse Events	Anastomosis stricture	2
Adverse Events	Anastomotic bleeding	1
Adverse Events	Anastomotic dehiscence	2
Adverse Events	Anastomotic leak	17
Adverse Events	Anastomotic leakage from GJ	1
Adverse Events	Anastomotic leakage from OJ	1
Adverse Events	Anastomotic leakage type 1	1
Adverse Events	Anastomotic leakage type 2	1
Adverse Events	Anastomotic stenosis	5
Adverse Events	Any complication	2
Adverse Events	ARDS	1

Adverse Events	Arteriosclerosis obliterans of the leg	1
Adverse Events	Ascites	2
Adverse Events	Atelectasis	2
Adverse Events	Atelectasis or pleural effusion	1
Adverse Events	Atrial fibrillation	1
Adverse Events	Bleeding	4
Adverse Events	Bleeding abdominal	1
Adverse Events	Bleeding from anastomosis	1
Adverse Events	Blood transfusion	2
Adverse Events	Blood transfusion volume	2
Adverse Events	Body temperature exceeding 37 ° C (days)	2
Adverse Events	Bowel obstruction	1
Adverse Events	Bowel obstruction/ileus	1
Adverse Events	Bronchopneumonia	1
Adverse Events	Bronchoscopic toilet	1
Adverse Events	Cardiac complications	6
Adverse Events	Cardiac failure	1
Adverse Events	Cardiocirculatory	1
Adverse Events	Cardiopulmonary disaese	1
Adverse Events	Catheter-induced sepsis	1
Adverse Events	Cerebrovascular	1
Adverse Events	Cholecystitis	3
Adverse Events	Cholecystitis acute	1
Adverse Events	Cholecystitis requiring percutaneous drainage	1
Adverse Events	Chyle leakage	2
Adverse Events	Chylous drainage	1
Adverse Events	Chylous lymphorrhea	1

Adverse Events	Colonic perforation	1
Adverse Events	Complications	3
Adverse Events	Complications after discharge	1
Adverse Events	Complications number of	1
Adverse Events	Deep vein thrombosis	3
Adverse Events	Delayed gastric emptying	2
Adverse Events	Delayed gastric emptying without obstruction	1
Adverse Events	Diarrhoea	1
Adverse Events	Drug-induced hepatitis	1
Adverse Events	Dumping syndrome	1
Adverse Events	Duodenal leak	1
Adverse Events	Duodenal stump leak	3
Adverse Events	Early dumping syndrome	1
Adverse Events	Early surgical complications	1
Adverse Events	Empyema thoracis	1
Adverse Events	Endocrine complications	1
Adverse Events	Endocrine events	1
Adverse Events	Enterocutaneous fistula	3
Adverse Events	Esophagus and remnant stomach infarction	1
Adverse Events	Fever	1
Adverse Events	Fluid collection	1
Adverse Events	Fluid collection/abscesses	1
Adverse Events	Gastric atonia	1
Adverse Events	Gastric remnant necrosis	1
Adverse Events	Gastrointestinal bleeding	1
Adverse Events	Gastrointestinal complications	1
Adverse Events	Gastrointestinal injury	1

Adverse Events	Gastroparesis	3
Adverse Events	Haemorrhage	2
Adverse Events	Hb	2
Adverse Events	Hepatic complications	2
Adverse Events	Hepatic failure	1
Adverse Events	Herpes zoster	1
Adverse Events	Hiccups	1
Adverse Events	Hospital morbidity	1
Adverse Events	Hypercapnia	1
Adverse Events	Iatrogenic spleen injury	1
Adverse Events	Idiopathic small bowel perforation	1
Adverse Events	Iileus mechanical	1
Adverse Events	Ileus	2
Adverse Events	Ileus adhesive	2
Adverse Events	Ileus paralytic	1
Adverse Events	Ileus prolonged	1
Adverse Events	Incision fat liquefaction	1
Adverse Events	Incision infection	1
Adverse Events	Infection	1
Adverse Events	Intestinal fistula	2
Adverse Events	Intestinal ischaemia	1
Adverse Events	Intestinal obstruction	3
Adverse Events	Intra-abdominal bleeding	3
Adverse Events	Intra-abdominal collections	1
Adverse Events	Intra-abdominal complications	1
Adverse Events	Intra-abdominal infection	1
Adverse Events	Intraluminal bleeding	3

Adverse Events	Intraoperative blood transfusion	2
Adverse Events	Intraoperative complications	3
Adverse Events	intraoperative major bleeding	1
Adverse Events	Intraperitoneal haemorrhage	1
Adverse Events	Late surgical complications	1
Adverse Events	Leakage	1
Adverse Events	Liver dysfunction	1
Adverse Events	Local complications	1
Adverse Events	Long-term complications	1
Adverse Events	Lung Infection	1
Adverse Events	Lymphatic leakage	1
Adverse Events	Lymphorrhoea	1
Adverse Events	Major abdominal infections	1
Adverse Events	Major cardiorespiratory incidents	1
Adverse Events	Major complications	2
Adverse Events	Major post-operative complication	1
Adverse Events	Major surgical complications	1
Adverse Events	Malabsorption	1
Adverse Events	Mediastinitis	1
Adverse Events	Medical complications	1
Adverse Events	Metabolic complications	1
Adverse Events	Minor complications	1
Adverse Events	Minor discharge of pancreatic juice	1
Adverse Events	Minor leakage	1
Adverse Events	Minor patchy pulmonary collapse	1
Adverse Events	Minor pulmonary atelectasis	2
Adverse Events	Morbidity	8

Adverse Events	Morbidity rate	1
Adverse Events	Multiple organ failure	1
Adverse Events	Myocardial infarction	1
Adverse Events	Nausea	1
Adverse Events	Need for blood transfusion	1
Adverse Events	Non-surgical Complications	6
Adverse Events	Number of patients with complications	1
Adverse Events	Operative complications	1
Adverse Events	Operative morbidity	3
Adverse Events	Other complications	7
Adverse Events	Overall complications	2
Adverse Events	Overall Post-operative complications	1
Adverse Events	Pancreas-related complications	1
Adverse Events	Pancreatic fistula	15
Adverse Events	Pancreatic injury	2
Adverse Events	Pancreatic leak	4
Adverse Events	Pancreatitis	1
Adverse Events	Pancreatitis acute	3
Adverse Events	Pancreatitis edematous	1
Adverse Events	Pancreatitis severe	1
Adverse Events	Pancreatitis traumatic	1
Adverse Events	Peri-operative complications	1
Adverse Events	Peritoneal haemorrhage	1
Adverse Events	Pleural	1
Adverse Events	Pleural effusion	5
Adverse Events	Pleural fluid	1
Adverse Events	Pneumonia	11

Adverse Events	Post-operative bleeding	1
Adverse Events	Post-operative complications	5
Adverse Events	Post-operative drain discharge	1
Adverse Events	Post-operative glucose tolerance	1
Adverse Events	Post-operative hemorrhage	2
Adverse Events	Post-operative major complications	1
Adverse Events	Post-operative minor complication	1
Adverse Events	Post-operative morbidity	1
Adverse Events	Post-operative psychosis	1
Adverse Events	Post-operative respiratory care	1
Adverse Events	Post-operative respiratory function	1
Adverse Events	Post-operative surgical parameters	1
Adverse Events	Post-operative symptoms	1
Adverse Events	Presence of gallstones	1
Adverse Events	Procedure-related morbidity and mortality	1
Adverse Events	Prolonged diarrhea	1
Adverse Events	Prolonged retention of intra-abdominal fluid	1
Adverse Events	Pulmonary	4
Adverse Events	Pulmonary complications	4
Adverse Events	Pulmonary edema	1
Adverse Events	Pulmonary embolism	2
Adverse Events	Pulmonary infection	1
Adverse Events	Pyothorax	1
Adverse Events	Rate of reinsertion of NG tube	1
Adverse Events	Recurrent laryngeal nerve palsy	1
Adverse Events	Reflux oesophagitis	2
Adverse Events	Re-laparotomy	2

Adverse Events	Renal complications	2
Adverse Events	Renal failure	2
Adverse Events	Re-operation	8
Adverse Events	Re-operation details	1
Adverse Events	Respirator use after surgery	1
Adverse Events	Respiratory complications	1
Adverse Events	Respiratory failure	1
Adverse Events	Return to theatre	1
Adverse Events	Septic complications	1
Adverse Events	Serious and potentially fatal complications	1
Adverse Events	Severe diarrhoea	1
Adverse Events	Severe feeding problem requiring prolonged hyperalimentation	1
Adverse Events	Severity of complications	2
Adverse Events	Severity of post-operative complications	1
Adverse Events	Short-term complications	1
Adverse Events	Small-bowel obstruction	1
Adverse Events	Splenic artery pseudoaneurysm	1
Adverse Events	Splenic injury	1
Adverse Events	Stasis	1
Adverse Events	Stenosis	1
Adverse Events	Surgical complications	8
Adverse Events	Surgical risk	1
Adverse Events	Systemic complications	1
Adverse Events	Systemic infections	1
Adverse Events	Thermal injury	1
Adverse Events	Thoracic effusion requiring thoracic drainage	1

Adverse Events	Thromboembolic complications	1
Adverse Events	Total complications	1
Adverse Events	Total major complications	1
Adverse Events	Total morbidity	4
Adverse Events	Tracheotomy	1
Adverse Events	Transfusion	3
Adverse Events	Transfusions received	1
Adverse Events	Transient ischemic attack	1
Adverse Events	Transient LFT abnormality	1
Adverse Events	Trocar related injury	1
Adverse Events	Tube tracheotomy	1
Adverse Events	Uncomplicated calf vein thrombosis	1
Adverse Events	Upper gastro-intestinal haemorrhage	1
Adverse Events	Urinary complications	2
Adverse Events	Urinary retention	1
Adverse Events	Urinary tract complications	1
Adverse Events	Urinary tract infection	3
Adverse Events	Viral infection	1
Adverse Events	Vomiting	1
Adverse Events	Wound abscess	1
Adverse Events	Wound complications	2
Adverse Events	Wound dehiscence	2
Adverse Events	Wound evisceration	1
Adverse Events	Wound haematoma	1
Adverse Events	Wound infection	10
Adverse Events	Wound infection/dehiscence	1
Adverse Events	Wound problem	1

Adverse Events	Wound seroma	2
Cost	Medical cost	1
Mortality	Death	8
Mortality	Death from a post-operative complication	1
Mortality	Death from all causes	1
Mortality	Death from gastric cancer as a cause	1
Mortality	Disease free survival	4
Mortality	Disease free survival 4-year	2
Mortality	Disease free survival 5-year	3
Mortality	Disease specific survival	1
Mortality	Disease specific survival 5-year	1
Mortality	Gastric cancer related deaths	1
Mortality	Hospital death	3
Mortality	Hospital mortality	7
Mortality	In-hospital mortality	2
Mortality	Mortality not related to surgery	1
Mortality	Operative death	1
Mortality	Operative mortality	3
Mortality	Overall survival	8
Mortality	Overall survival 10-year	1
Mortality	Overall survival 3-year	2
Mortality	Overall survival 5-year	6
Mortality	Overall survival 6-year	1
Mortality	Overall survival 7-year	1
Mortality	Post-operative death	4
Mortality	Post-operative mortality	3
Mortality	Post-operative survival	1

Mortality	Recurrence-free survival	5
Mortality	Relapse-free survival	1
Mortality	Survival 11-year	1
Mortality	Survival 5-year	11
Mortality	Survival Period	1
Mortality	Treatment related deaths	1
Patient Pathway	Days of hospitalization	1
Patient Pathway	Duration of hospital stay	12
Patient Pathway	Duration of post-operative hospital stay	11
Patient Pathway	Readmission	1
Patient Reported Outcomes	Degree of pain	1
Patient Reported Outcomes	Overall satisfaction	1
Patient Reported Outcomes	Pain	1
Patient Reported Outcomes	Post-operative Pain	1
Patient Reported Outcomes	QoL	3
Recovery From Surgery	4-day post-operative use of analgesics	1
Recovery From Surgery	Blood urea nitrogen	1
Recovery From Surgery	Body weight	1
Recovery From Surgery	CK	1
Recovery From Surgery	CRP	4
Recovery From Surgery	CRP 3 days after surgery	1
Recovery From Surgery	Days of fever	1
Recovery From Surgery	Days to sips of water	1
Recovery From Surgery	Decrease in body weight	1
Recovery From Surgery	Decrease of body weight 1 month after the operation	1
Recovery From Surgery	Dose of analgesic (mg)	1
Recovery From Surgery	Duration of pain control	1

Recovery From Surgery	Eating	1
Recovery From Surgery	FEV1(L)	1
Recovery From Surgery	FEVC(L)	1
Recovery From Surgery	Fever	1
Recovery From Surgery	First eating (post-operative day)	1
Recovery From Surgery	First walking (post-operative day)	1
Recovery From Surgery	Food intake	1
Recovery From Surgery	Frequency of analgesics injection	1
Recovery From Surgery	Frequency of injection given according to analgesic requests	1
Recovery From Surgery	IL-6	1
Recovery From Surgery	Immediate postoperative inflammatory and immune responses	1
Recovery From Surgery	Immunological response to surgery	2
Recovery From Surgery	Lean body mass	1
Recovery From Surgery	LFT	1
Recovery From Surgery	Number of days to get out of bed	1
Recovery From Surgery	Nutritional Status	1
Recovery From Surgery	Pain control	2
Recovery From Surgery	Post-operative analgesia	1
Recovery From Surgery	Post-operative course	1
Recovery From Surgery	Post-operative pain	1
Recovery From Surgery	Post-operative recovery	1
Recovery From Surgery	Prealbumin	1
Recovery From Surgery	Progression of oral intake	1
Recovery From Surgery	Pulmonary function	1
Recovery From Surgery	Recovery of Physical Activity	1
Recovery From Surgery	Residual pain at day 7	1

Recovery From Surgery	SaO2	1
Recovery From Surgery	Serum Albumin	1
Recovery From Surgery	Surgical stress response	1
Recovery From Surgery	The early recovery course	1
Recovery From Surgery	Time of first flatus/index of peristalsis recovery	1
Recovery From Surgery	Time to ambulation	1
Recovery From Surgery	Time to first flatus	4
Recovery From Surgery	Time to first flatus (days)	5
Recovery From Surgery	Time to first liquid intake	1
Recovery From Surgery	Time to first soft diet uptake	1
Recovery From Surgery	Time to food intake	1
Recovery From Surgery	Time to liquid diet	1
Recovery From Surgery	Time to removal of epidural anesthesia (days)	1
Recovery From Surgery	Time to sips of water	2
Recovery From Surgery	Time to start oral intake (days)	1
Recovery From Surgery	Time until removal of the naso-gastric tube	1
Recovery From Surgery	Time until start of meals	1
Recovery From Surgery	Times analgesic given	1
Recovery From Surgery	Times of pain rescue	1
Recovery From Surgery	Total amount of analgesics infused	1
Recovery From Surgery	Total body weight	1
Recovery From Surgery	Total protein	1
Recovery From Surgery	Walking	1
Recovery From Surgery	WBC	1
Recovery From Surgery	WCC	3
Surviving & Controlling Cancer	Cumulative risk of recurrence	2

Surviving & Controlling Cancer	Disease recurrence rate	1
Surviving & Controlling Cancer	Port site metastasis	2
Surviving & Controlling Cancer	Recurrence	4
Surviving & Controlling Cancer	Recurrence patterns	1
Surviving & Controlling Cancer	Recurrent disease	1
Surviving & Controlling Cancer	Tumor recurrence	1
Technical Aspects of Surgery	Amount of bleeding	1
Technical Aspects of Surgery	Blood loss	7
Technical Aspects of Surgery	Clear margin distance	1
Technical Aspects of Surgery	Conversion to open surgery	3
Technical Aspects of Surgery	Dissected Lymph nodes - mediastinal	1
Technical Aspects of Surgery	Dissected Lymph nodes - para-aortic	1
Technical Aspects of Surgery	Distal resection margin	2
Technical Aspects of Surgery	Duration of surgery	3
Technical Aspects of Surgery	Estimated blood loss	5
Technical Aspects of Surgery	Intraoperative blood loss	7
Technical Aspects of Surgery	Length of laparotomy incision	1
Technical Aspects of Surgery	Length of lesser curvature of resected stomach	1
Technical Aspects of Surgery	Length of lesser curvature of resected stomach	1
Technical Aspects of Surgery	Length of longest wound	1
Technical Aspects of Surgery	Length of resection on greater cureve	1

Technical Aspects of Surgery	Length of resection on lesser curve	1
Technical Aspects of Surgery	Main wound size	1
Technical Aspects of Surgery	Mean blood loss	2
Technical Aspects of Surgery	Mean operating time	1
Technical Aspects of Surgery	Number of lymph nodes dissected or resected or retrieved	22
Technical Aspects of Surgery	Number of lymph nodes removed N1 group	1
Technical Aspects of Surgery	Number of lymph nodes removed N2 group	1
Technical Aspects of Surgery	Number of lymph nodes removed N3 group	1
Technical Aspects of Surgery	Number of lymph nodes removed N4 group	1
Technical Aspects of Surgery	Operative blood loss	2
Technical Aspects of Surgery	Operative time	18
Technical Aspects of Surgery	Pathological outcomes	1
Technical Aspects of Surgery	Proximal margin positive/negative	1
Technical Aspects of Surgery	Proximal resection margin	2
Technical Aspects of Surgery	R0 resection	2
Technical Aspects of Surgery	Radicality R0	1
Technical Aspects of Surgery	Radicality R1	1
Technical Aspects of Surgery	Resection line involvement - distal	1
Technical Aspects of Surgery	Resection line involvement - proximal	1
Technical Aspects of Surgery	Residual Tumour	1
Technical Aspects of Surgery	Residual tumour R0	2
Technical Aspects of Surgery	Residual tumour R1/2	1
Technical Aspects of Surgery	Residual tumour R1/2	1
Technical Aspects of Surgery	Surgical time	2
Technical Aspects of Surgery	Time for operation	1
Technical Aspects of Surgery	Wound size	5
