# Supplementary material for: Reporting of outcomes in gastric cancer surgery trials: a systematic review
Source: BMJ Open. 2018 Oct 17;8(10):e021796. doi: 10.1136/bmjopen-2018-021796 (PMC6196805; doi:10.1136/bmjopen-2018-021796)
Supplement: Author's manuscript [file bmjopen-2018-021796.draft_revisions.pdf]

BMJ Open is committed to open peer review. As part of this commitment we make the peer review history of every article we publish publicly available.

When an article is published we post the peer reviewers' comments and the authors' responses online. We also post the versions of the paper that were used during peer review. These are the versions that the peer review comments apply to.

The versions of the paper that follow are the versions that were submitted during the peer review process. They are not the versions of record or the final published versions. They should not be cited or distributed as the published version of this manuscript.

BMJ Open is an open access journal and the full, final, typeset and author-corrected version of record of the manuscript is available on our site with no access controls, subscription charges or pay-per-view fees (http://bmjopen.bmj.com).

If you have any questions on BMJ Open's open peer review process please email info.bmjopen@bmj.com

BMJ Open

Reporting of Outcomes in Gastric Cancer Surgery Trials: A Systematic Review.

Journal:	*BMJ Open*
Manuscript ID	bmjopen-2018-021796
Article Type:	Research
Date Submitted by the Author:	22-Jan-2018

Complete List of Authors:	Alkhaffaf, Bilal; Department of Oesophago-Gastric Surgery<br>Blazeby, Jane; University of Bristol, Centre for Surgical Research<br>Williamson, Paula; University of Liverpool<br>Bruce, Iain; Manchester University Hospitals NHS Foundation Trust, Paediatric ENT Department<br>Glenny, Anne-Marie; University of Manchester, Division of Dentistry

Keywords:	Surgical Oncology, Stomach Neoplasms, Outcome Assessment, Delphi Technique, Patient Reported Outcome Measures, Outcome reporting

SCHOLARONE™
Manuscripts

For peer review only

Title Page

Reporting of Outcomes in Gastric Cancer Surgery Trials: A Systematic Review.

Authors

Bilal Alkhaffaf[1,2], Jane M Blazeby[3], Paula R Williamson[4], Iain A Bruce[5,6] & Anne-Marie Glenny[7]

Affiliations

[1]Department of Oesophago-Gastric Surgery, Manchester University Hospitals NHS foundation Trust, Manchester Academic Health Science Centre, Manchester, UK

[2]Division of Molecular & Clinical Cancer, Faculty of Biology, Medicine and Health, University of Manchester

[3]Centre for Surgical Research, University of Bristol

[4]MRC North West Hub for Trials Methodology Research, University of Liverpool

[5]Paediatric ENT Department, Royal Manchester Children's Hospital, Manchester University Hospitals NHS Foundation Trust, Manchester Academic Health Science Centre, Manchester, UK

[6]Division of Infection, Immunity and Respiratory Medicine, Faculty of Biology, Medicine and Health, University of Manchester, Manchester, UK

[7]Division of Dentistry, University of Manchester

Corresponding Author

Mr Bilal Alkhaffaf
Consultant Oesophago-Gastric Surgeon
Manchester University Hospitals NHS Foundation Trust
Manchester Royal Infirmary
Oxford Road
M13 9WL
E-mail: bilal.alkhaffaf@mft.nhs.uk

For peer review only - http://bmjopen.bmj.com/site/about/guidelines.xhtml

Telephone: +44 161 276 1234

Manuscript date: 17/01/2018

Reprints

Reprints will not be available from the authors.

Funding

This study is funded by the National Institute for Health Research (NIHR) Doctoral Research Fellowship Grant (DRF-2015-08-023).

Disclaimer

This paper presents independent research funded by the National Institute for Health Research (NIHR). The views expressed are those of the author(s) and not necessarily those of the NHS, the NIHR or the Department of Health.

Running Title

Reporting of Outcomes in Gastric Cancer Surgery Trials.

Word Count: 2709

Number of tables: 5

Number of figures: 2

Number of online only appendices: 1

For peer review only

Structured Abstract

Objective

This review examines the degree of variation in the reporting of outcomes used in gastric cancer surgery trials.

Background

The development of clinical guidelines for the surgical management of gastric cancer should be based on robust evidence from well-designed trials. Being able to reliably compare and combine the outcomes of these trials is a key factor in this process.

Methods

Systematic literature searches and a review of study protocols were undertaken to identify randomized controlled trials (RCTs), published between 1996 and 2016, investigating therapeutic surgical interventions for gastric cancer. Outcomes were listed verbatim, categorized into groups (outcome themes) and examined for definitions and measurement instruments.

Results

Of 1919 abstracts screened, 32 trials (9,073 participants) were identified. A total of 749 outcomes were reported of 96 (13 per cent) were accompanied by an attempted definition. No single outcome was reported by all trials. ‘Adverse events’ was the most frequently reported ‘outcome theme’ in which 240 unique terms were described. 12 trials (38%) classified complications according to severity, with 5 (16%) using a formal classification system (Clavien-Dindo or Accordion scale). Of 27 trials which described ‘short-term’ mortality, 15 (47%) used one of 5 different definitions. Six out of the 32 trials (19%) described ‘patient-reported outcomes’.

Conclusion

Reporting of outcomes in gastric cancer surgery trials is inconsistent. A consensus approach to develop a minimum set of well-defined, standardized outcomes to be used by all future trials

For peer review only - http://bmjopen.bmj.com/site/about/guidelines.xhtml

examining therapeutic surgical interventions for gastric cancer is needed. This should consider the views of all key stakeholders, including patients.

KEYWORDS:

Surgical Oncology

Stomach Neoplasms

Outcome Assessment

Delphi Technique

Patient Reported Outcome Measures

Treatment Outcome

Outcome reporting

For peer review only

Strengths & Limitations of the Study

This study is based on a reproducible and transparent methodology which has been subjected to critical appraisal during a peer-review process.

The GASTROS study, of which this review forms the first stage, is advised by a Study Advisory Group which includes patient representatives.

Including non-English and non-randomized studies in our search strategy may have yielded a greater number of outcomes.

For peer review only

For peer review only - http://bmjopen.bmj.com/site/about/guidelines.xhtml

Main Article

Introduction

Background

Gastric cancer remains a leading cause of cancer-related death globally[1]. Long-term survival remains poor and has not improved significantly over the last four decades[2]. Whilst there has been a shift to multi-modal therapy over the last decade, surgery remains the primary method of curative treatment. Many developments in surgical techniques aim to improve long-term survival, whilst minimizing post-operative complications. Understanding which of these approaches are optimal for patients should be based on robust evidence from well-designed trials. This process involves the synthesis of evidence in the form of systematic reviews which can only be reliably undertaken if trials report the same outcomes and measure them in the same manner.

Aims & Objectives

This review forms part of the first stage of a three-stage study, which intends to examine and address problems with inconsistent outcome reporting in gastric cancer surgery trials (GASTROS – **GA**stric Cancer **S**urgery **TR**ials **R**eported **O**utcome **S**tandardisation). The study aims to develop a 'core outcome set' (COS) – a *minimum* group of standardized and well-defined outcomes, relevant to key stakeholders and measured by all trials[3] – to standardize the reporting of outcomes in randomized control trials within this field. Our previously published study protocol contains an overview of all three stages[4] . This review specifically aims to examine the degree of variation in the reporting of outcomes described by gastric cancer surgery trials. Additionally, the outcomes reported by trials in this review will then be used to generate a 'long-list' of potentially important outcomes which will be prioritized during a Delphi survey in stage two of the study.

For peer review only - http://bmjopen.bmj.com/site/about/guidelines.xhtml

Methods

Definitions

The GASTROS study, and more specifically this review, focuses on outcome reporting in 'therapeutic surgical trials'. A 'surgical trial' has been previously defined as one of the following [5]:

- Type 1 - A trial of medical interventions in surgical patients
- Type 2 - A trial which compares a surgical intervention to another surgical intervention
- Type 3 - A trial which compares a surgical intervention to a non-surgical intervention

The GASTROS study focuses on 'type 2' trials. In the context of gastric cancer, a 'therapeutic surgical intervention' is defined as a potentially curative procedure which aims to excise the gastric neoplasm resulting in partial or total organ loss.

Search strategy

A summary of the review's inclusion and exclusion criteria is summarised in table 1.

Timeline

Trials were searched from 1996 when the first CONSORT statement for the reporting of RCTs was published up to and including March 2016. The COS aims to influence trial design regardless of the country of origin of participating centers and patients. Many of the surgical interventions (e.g. D2 lymphadenectomy) which have recently been examined in the West have long been established practice in the Far East and Asia. Searching trials over a 20-year period allows a comprehensive understanding of which outcomes have been measured for similar trials regardless of the trial location.

Identifying studies

Detailed search strategies were developed for each of the following electronic databases examined:

- Evidence Based Medicine Reviews via OVID
    - Cochrane Database of Systematic Reviews 2005 to March 30, 2016,
    - ACP Journal Club 1991 to March 2016,

For peer review only - http://bmjopen.bmj.com/site/about/guidelines.xhtml

- Database of Abstracts of Reviews of Effects 1st Quarter 2016
- Cochrane Central Register of Controlled Trials February 2016
- Health Technology Assessment 1st Quarter 2016
- NHS Economic Evaluation Database 1st Quarter 2016

- MEDLINE via OVID (January 1st 1996 to March 30, 2016)
- EMBASE via OVID (January 1st 1996 to March 30, 2016);
- CINAHL via EBSCO (January 1st 1996 to March 30, 2016).

In order to identify surgical interventions and outcome measures being used in current studies, we searched the following databases for protocols of ongoing trials, including completed trials not yet published:

- The US National Institutes of Health Trials Register (http://clinicaltrials.gov);
- The WHO International Clinical Trials Registry Platform (http://apps.who.int/trialsearch/default.aspx).

Non-English language studies were excluded from this review due to resource limitations. Trials published only as conference abstracts were excluded as they are often limited by 'word count' and hence the abstract would not represent a comprehensive list of outcomes measured in the respective study.

Assessment of eligibility

For quality assurance, two review authors (BA and AMG) independently screened the titles and abstracts retrieved from the electronic searches. This assessment was undertaken in groups of ten abstracts in reverse chronological order. Once there was complete agreement with two consecutive groups of ten abstracts, the remaining abstracts were split and each reviewer screened independently. Full text copies of all study publications that appeared to meet the inclusion criteria were obtained.

For peer review only - http://bmjopen.bmj.com/site/about/guidelines.xhtml

For peer review only

Full text copies were also obtained where there was insufficient information in the title or abstract to make a clear judgement.

BA and AMG independently assessed the full text copies for eligibility. This assessment was undertaken in groups of ten publications in reverse chronological order. Once there was complete agreement with two consecutive groups of ten abstracts, the remaining publications were split and each reviewer extracted data independently. Any disagreements were resolved through discussion. Any unresolved disagreements were referred to the GASTROS study management team for a final decision.

Data Extraction

BA and AMG independently reviewed all eligible publications and extracted data into a Microsoft Excel (Version 2013, Microsoft, Washington, DC, USA) spreadsheet.

Publication versus Study

It is not uncommon that investigators publish results at different stages of their trial and with each publication present a new set of outcomes. The GASTROS study team decided to amalgamate the outcomes published in all publications associated with a single trial to more fairly reflect outcomes being reported by research groups.

Demographics

The following demographic data were recorded for each trial:

1. Author details
2. Title of publication
3. Journal
4. Year of publication
5. Number of participating centres

For peer review only - http://bmjopen.bmj.com/site/about/guidelines.xhtml

6. Country of 1st Author
7. Countries of participating centres
8. Total number of patients recruited to the study
9. Length of follow up
10. Interventions being investigated

Outcomes

The following data were recorded for each outcome:

1. Outcome measured (and whether stated as primary or secondary outcome). Where a primary outcome was not explicitly stated, the outcome on which the sample size calculation was based was taken as the primary outcome.
2. Whether the outcome was defined or not. Outcomes were considered defined if text of their meaning or a citation was provided.
3. The definition of the outcome.
4. The method of outcome measurement (indicators and/or tools used, if relevant).
5. Time points and time-period at or during which the outcome was measured (for example quality of life at 3-months post-surgery).

Rationalizing & Grouping Outcomes

Outcomes were extracted verbatim from publications and minimal merging of terms was undertaken. Outcomes were merged only where terms were clearly identical. For example, ‘anastomotic leak’, ‘anastomotic leak**age**’ and ‘anastomotic leak**s**’ would be merged into ‘anastomotic leak’. Outcomes were organised into ‘outcome themes’ **-** broad categories encompassing similar types of outcomes.

For peer review only - http://bmjopen.bmj.com/site/about/guidelines.xhtml

Patient & Public Involvement

A Study Advisory Group forms part of the management structure of the wider GASTROS study[4], of which this review forms part of the first stage. The SAG is made up of key stakeholder representatives including patients, oncology nurses and surgeons. The group provides advice on the methodology of the study, general delivery of the study against its stated objectives, and ensures that the viewpoints of all stakeholder groups are considered.

For peer review only

For peer review only - http://bmjopen.bmj.com/site/about/guidelines.xhtml

Table 1. Inclusion and exclusion criteria for this review.

	Included	*Excluded*
Types of Studies	• Type 2* surgical randomized controlled trials (RCTs) and protocols of surgical RCTs (all trial phases).<br>• Systematic reviews of type 2 surgical RCTs.<br>• English Language studies.	• Type 1 or type 3* surgical RCTs and systematic reviews of type 1 or type 3 RCTs.<br>• Non-randomized studies.<br>• Non-English language studies.
Population	• Patients aged 18 years and over.	• Patients below the age of 18.
Interventions	• Partial or total gastrectomy.<br>• Surgery with curative intent.	• Esophagectomy for gastro-esophageal junctional tumors.<br>• Surgery with non-curative intent (i.e. in stage 4 cancer with prior expectation of an R1 or R2 resection) for the relief of symptoms such as gastric outlet obstruction or bleeding.<br>• Endoscopic interventions.
Conditions	• Invasive cancer of the stomach and gastro-esophageal junction.	• Dysplasia or non-invasive gastric neoplasms.<br>• Sarcoma (including gastrointestinal stromal tumors).<br>• Gastric lymphoma.

*Type 1 - a trial of medical interventions in surgical patients, type 2 - a trial which compares a surgical intervention to another surgical intervention, type 3 - a trial which compares a surgical intervention to a non-surgical intervention[5].

For peer review only - http://bmjopen.bmj.com/site/about/guidelines.xhtml

Results

Summary

A total of 1,919 abstracts were screened which resulted in the identification of 48 publications from 32 trials (having recruited a total of 9073 patients) eligible for inclusion in the review (figure 1). A further 875 protocols were screened which identified 24 active or unpublished trials aiming to recruit 10,761 patients. A summary of all trials included in the analysis is described in table 2. During the data extraction process, no disagreements requiring discussion in the study management group arose between the two independent reviewers. A total of 749 (392 unique terms) outcomes were reported of which 13% (96 out of 749) were accompanied by an attempted definition. Thirty-eight percent of trials (12 out of 32) described a primary outcome or provided a sample size calculation. No single outcome was reported in every trial.

Analysis of Outcomes According to Themes

Outcomes were organised into eight 'outcome themes', illustrated in figure 2 and described in table 3. A comprehensive list of reported outcomes is presented in appendix 1.

Mortality

Death after surgery was generally described as 'short-term' and 'long-term' survival. Long-term survival was used as a primary outcome measure in 41% of trials (13 out of 32). The terms used to describe long-term mortality and the time-points at which they were measured was inconsistent (table 4). 'Short-term' mortality was reported by 84% of trials (27 out of 32) of which fifteen provided one of the following definitions (frequency each definition was used is presented in brackets):

1. *'Death within **30-days** of surgery' (3/15)*
2. *'Death of **any cause** within **30 days**, or death within the **same hospitalization' (9/15)***
3. ***'In-hospital** deaths and 'deaths' **within 1 month' (1/15)***
4. *'Death within **30 days** of the operation or during **any hospital stay' (1/15)***
5. ***'Any death** that occurred during the **hospital stay (1/15)***

For peer review only - http://bmjopen.bmj.com/site/about/guidelines.xhtml

Adverse Events

Adverse events were the commonest outcome theme to be reported and made up half of the ten most reported outcomes (table 5). 'Anastomotic leak' was the commonest adverse event to be reported and was described using 5 different definitions (frequency each definition was used is presented in brackets):

- *'Clinical and radiological diagnosis' (2).*
- *'Confirmed by gastrointestinal x-ray imaging, endoscopy, or angiography' (1).*
- *'Dehiscence confirmed by radiographic examination using contrast medium' (1).*
- *'Type I anastomotic leakage: a small localized leakage at the esophagojejunal anastomosis, without pleural or abdominal spillage, demonstrated by radiologic studies with barium' (1).*
- *'Type II anastomotic leakage: an important dehiscence of the esophago-jejunal anastomosis, with pleural or abdominal dissemination, appearance of intestinal content through the drains, a positive methylene blue test (appearance of orally ingested methylene blue through the drains), and clear demonstration of this leakage by radiologic contrast studies' (1).*

Adverse events were categorized by 12 out of 32 trials using terms including 'major', 'minor', 'short-term' and 'long-term'. Five trials used a formal classification system (Clavien-Dindo Classification or Accordian Severity Grading).

Patient Reported Outcomes

Patient-reported Outcomes (PROs) were reported in 19% of trials (6 out of 32) and included measures of quality of life (QoL) (n=3) and 'pain' (n=3). QoL was measured using validated tools for gastric cancer in two trials (EORTC QLQ-C30 with QLQ and Spitzer QoL Index) and a non-validated tool in one trial. Pain was measured using 3 different visual-analogue scales.

For peer review only

Findings from Study Protocols

Most of the 24 ongoing or unpublished trials [6–29] are recruiting in China (n=13), with twenty examining 'extent of lymphadenectomy' or minimally invasive approaches to surgery. A total of 220 uniquely termed outcomes are planned to be reported, thirty-five of which (16%) have an accompanying definition in the respective protocol. The commonest term used to report 'long-term survival' is 'overall survival' (OS) which will be measured by sixteen trials. Seven of these trials plan to measure OS after 5-years of follow-up, three at 3-years of follow-up, and six did not identify time points at which OS would be measured. At the time of our search, one trial protocol contained no information about which outcomes are to be measured.

QoL is due to be measured by ten trials (42 per cent) with five trials proposing to use one or a combination of four different measurement instruments (EORTC QLQ-C30 with QLQ-STO22, SF-36, GIQLI and Euro-Quality of Life-5D). Seven protocols described the timing of the quality of life measurements as follows:

- *'Preoperative, postoperative* ***3 weeks*** *and postoperative* ***12 months'.***
- *'In* ***pre-therapy <7 days****,* ***pre-operative <7 days****, and post-operative at* ***12 months*** *after surgery'.*
- ***'90th postoperative day'.***
- ***'Regularly*** *for three years after surgery'.*
- ***'Preoperatively****,* ***five days postoperatively****,* ***three*** *months,* ***six*** *months and* ***one year*** *postoperatively'.*
- ***'Baseline****,* ***1 week****,* ***1*** *month,* ***6****-month,* ***1 year****,* ***3 years'.***
- ***'6 weeks****,* ***12****,* ***24****,* ***36****,* ***48*** *and* ***50 months*** *after surgery'.*

For peer review only - http://bmjopen.bmj.com/site/about/guidelines.xhtml

For peer review only

Table 2. Published gastric cancer surgery trials included in the study analysis.

Trial Reference(s)	Trial Number	Author	Year of 1st Publication	Participating Countries	Number of Recruiting Sites	Patients Recruited	Interventions
30–33	1	Hartgrink et al, Bonenkamp et al., Sasako et al., Songun et al.	1995	Netherlands	80	1078	• D1 lymphadenectomy<br>• D2 lymphadenectomy
34,35	2	Cuschieri et al.	1996	UK	32 surgeons*	737	• D1 lymphadenectomy<br>• D2 lymphadenectomy
36,37	3	Marubini et al. Bozzetti et al.	1999	Italy	31	615	• D2 subtotal gastrectomy<br>• D2 total gastrectomy
38	4	Maeta et al	1999	Japan	1	70	• D3 total gastrectomy<br>• D4 total gastrectomy
39	5	Furukawa et al.	2000	Japan	1	110	• Total gastrectomy and distal pancreatectomy

For peer review only - http://bmjopen.bmj.com/site/about/guidelines.xhtml

							• Pancreas preserving total gastrectomy
40	6	Csendes et al.	2002	Chile	1	187	• D2 total gastrectomy with splenectomy<br>• Spleen preserving D2 total gastrectomy
41	7	Kitano et al.	2002	Japan	1	28	• Laparoscopic assisted distal gastrectomy<br>• Open distal gastrectomy
42	8	Fujii et al	2003	Japan	1	20	• Laparoscopic assisted distal gastrectomy<br>• Open distal gastrectomy
43–45	9	Degiuili et al	2004	Italy	5	267	• D1 lymphadenectomy<br>• D2 lymphadenectomy
46–49	10	Sano et al.,<br>Sasako et al.,<br>Kodera et al,<br>Tsujinaka et al.	2004	Japan	24	523	• D2 lymphadenectomy<br>• D2 lymphadenectomy & para-aortic node dissection

For peer review only - http://bmjopen.bmj.com/site/about/guidelines.xhtml

[50]	11	Shibata et al	2004	Japan	9	81	• Pylorus-preserving gastrectomy<br>• Distal gastrectomy
[51]	12	Inaba et el.	2004	Japan	1	410	• Midline laparotomy<br>• Transverse laparotomy
[52]	13	Lee et al.	2005	South Korea	1	47	• Laparoscopic assisted D2 distal gastrectomy<br>• Open D2 distal gastrectomy
[53]	14	Hayashi et al	2005	Japan	1	28	• Laparoscopic assisted distal gastrectomy with extra-perigastric node dissection.<br>• Open distal gastrectomy with extra-perigastric node dissection.
[54]	15	Huscher et al	2005	Italy	1	59	• Laparoscopic sub-total gastrectomy<br>• Open sub-total gastrectomy
[55–58]	*16*	*Wu et al.*	2006	Taiwan	1	221	• D1 lymphadenectomy<br>• D3 lymphadenectomy
[59,60]	17	Sasako et al.	2006	Japan	27	167	• Left Thoracoabdominal approach

For peer review only - http://bmjopen.bmj.com/site/about/guidelines.xhtml

For peer review only

		Kurokawa et al.					• Abdominal trans-hiatal approach
61	18	Yu et al	2006	South Korea	1	207	• Total gastrectomy with splenectomy<br>• Spleen preserving total gastrectomy
62	19	Kulig et al.	2007	Poland	6	275	• D2 lymphadenectomy<br>• D2 lymphadenectomy & para-aortic node dissection
63,64	20	Kim et al.	2008	South Korea	1	164	• Laparoscopic assisted distal gastrectomy<br>• Open distal gastrectomy
65	21	Yonemura et al.	2008	Japan, Taiwan, South Korea	10	269	• D2 lymphadenectomy<br>• D4 lymphadenectomy
66,67	22	Kim et al.	2010	South Korea	13	1416	• Laparoscopic assisted D2 distal gastrectomy<br>• Open distal D2 gastrectomy
68	23	Imamura et al	2011	Japan	11	210	• D2 gastrectomy and bursectomy

For peer review only - http://bmjopen.bmj.com/site/about/guidelines.xhtml

							• D2 gastrectomy without bursectomy
69	24	Cai et al.	2012	China	1	123	• Laparoscopic assisted distal gastrectomy<br>• Open distal gastrectomy
70	25	Chen Hu et al.	2012	China	1	88	• Laparosopic gastrectomy<br>• Open gastrectomy<br>• Standard post-operative protocol<br>• Fast-track post-operative protocol
71	26	Takiguchi	2013	Japan	1	40	• Laparoscopic assisted distal gastrectomy<br>• Open distal gastrectomy
72	27	Lee et al.	2013	South Korea	1	204	• D2 distal gastrectomy<br>• D2 total gastrectomy
73	28	Sakuramoto et al	2013	Japan	1	64	• Laparoscopic assisted distal gastrectomy<br>• Open distal gastrectomy
74	29	Aoyama et al	2014	Japan	1	26	• Laparoscopic assisted distal

For peer review only - http://bmjopen.bmj.com/site/about/guidelines.xhtml

							gastrectomy<br>• Open distal gastrectomy
[75]	*30*	*Hirao et al.*	2015	Japan	11	210	• D2 gastrectomy with bursectomy<br>• D2 gastrectomy without bursectomy
[76]	31	Galizia et al	2015	Italy	1	73	• D1'+' total gastrectomy<br>• D2 total gastrectomy
[77]	32	Hu et al.	2016	China	14	1056	• Laparoscopic assisted D2 distal gastrectomy<br>• Open D2 distal gastrectomy

*Number of sites not specified.

For peer review only

For peer review only - http://bmjopen.bmj.com/site/about/guidelines.xhtml

Table 3. Number of times at least one outcome from respective theme was reported in published trials and unpublished or actively recruiting trial protocols.

**Outcome Theme**	**Theme Definition**	Published Trials (n=32) (%)	Unpublished or Actively Recruiting Trial Protocols (n=23**) (%)
Cost	Relating to delivery of surgery as part of clinical care within a healthcare system.	1 (3)	5 (23)
Patient Pathway	Outcomes related to the flow of patients through the healthcare system (e.g. hospital stay, readmission).	20 (63)	4 (17)
Patient-Reported Outcomes	Outcomes taken from the patient perspective.*	6 (19)	13 (57)
Surviving & Controlling Cancer	Measures of disease recurrence or disease progression.	15 (47)	17 (74)
Mortality	Outcomes related to short- and long-term survival/death rates and cause of death.	27 (84)	19 (83)
Technical Aspects of Surgery	Outcomes recorded directly in the operating theatre (e.g. operation time, blood loss).	31 (97)	13 (57)
Recovery from Surgery	Report of patient condition following surgery and the ability to return to preoperative or premorbid state.	16 (50)	9 (39)
Adverse Events	Forms of short- and long-term postoperative complications following surgery.	31 (97)	18 (78)

* Certain patient-reported outcomes may fall under other 'themes', e.g. 'post-operative pain' may relate to 'recovery from surgery'. ** One trial protocol contained no information about planned outcomes to report, therefore 23 out of total 24 trials were included in this table.

For peer review only - http://bmjopen.bmj.com/site/about/guidelines.xhtml

Table 4. Reporting of 'long-term mortality' in gastric cancer surgery trials.

Term used	Number of trials reporting outcome	Follow-up period used	Frequency defined	Definitions provided
'Overall Survival' including 'death from all causes'	19	Not described<br>3 years<br>5 years<br>6 years<br>7 years<br>10 years	7	• Date of randomisation until the day of death or the day of last follow-up (censored).<br>• Date of surgery to the date of death from any cause, censoring the follow-up time at the most recent date for living patients.<br>• Date of randomization to the date of death.<br>• Date of randomization to the date of death from any cause.<br>• OS included operative deaths.<br>• OS excluded post-operative deaths.
'Survival' including 'survival period'	13	Not described<br>5 years<br>11 years	4	• Survival excluding operative mortality<br>• Survival 5 years after curative surgery
'Disease-specific survival'	4	Not described	1	• Proportion of patients who had not died from gastric cancer.

For peer review only - http://bmjopen.bmj.com/site/about/guidelines.xhtml

including 'gastric cancer related deaths'		5 years		
*Disease-free survival**	1	Not described	1	• Time from randomization to recurrence or death due to any cause.
*Recurrence-free survival**	2	Not described	2	• Time from randomization to either the first recurrence or death from any cause.<br>• Time from randomization to the first documentation of cancer recurrence or death from any cause.

*Although these terms do not relate to mortality, they have been included in this table as the definitions provided by papers describe death as an end-point.

For peer review only

For peer review only - http://bmjopen.bmj.com/site/about/guidelines.xhtml

Table 5. The ten most frequently reported outcomes.

Outcome	Theme	Number of trials reporting outcome	Trials reporting the outcome*
Number of lymph nodes dissected/resected/retrieved	Technical aspects of surgery	22	2, 3, 5, 7, 9, 10, 13, 14, 15, 16, 17, 18, 19, 20, 21, 23, 24, 25, 26, 29, 31, 32
Operative time	Technical aspects of surgery	18	4, 5, 7, 8, 10, 11, 13, 14, 15, 16, 20, 22, 23, 24, 25, 26, 27, 30
Anastomotic leak	Adverse events	17	1, 2, 4, 6, 10, 14, 16, 17, 19, 22, 23, 24, 25, 27, 28, 31, 32
Pancreatic fistula	Adverse events	15	1, 2, 4, 5, 10, 16, 17, 19, 22, 23, 26, 27, 28, 29, 32
Duration of hospital stay	Patient pathway	12	1, 2, 5, 6, 7, 9, 13, 15, 20, 23, 26, 28
Duration of post-operative hospital stay	Recovery from surgery	11	2, 7, 9, 11, 14, 16, 19, 24, 25, 27, 32
Pneumonia	Adverse events	11	7, 10, 11, 12, 17, 23, 27, 28, 29, 31, 32
'5-year' survival	Mortality	11	1, 2, 6, 9, 10, 16, 17, 18, 19, 22, 26
Wound infection	Adverse events	10	2, 6, 12, 15, 16, 18, 19, 22, 24, 28
Abdominal abscess	Adverse events	10	5, 9, 10, 16, 17, 19, 23, 26, 28, 29

*See table 1 for trial numbers and associated publications.

For peer review only - http://bmjopen.bmj.com/site/about/guidelines.xhtml

Discussion

This review demonstrates significant inconsistencies in the outcomes measured in trials examining therapeutic surgical interventions for gastric cancer. Not only is there disagreement about 'what' outcomes should be measured, but also 'when' and 'how' they should be measured. Consequently, undertaking meta-analyses and systematic reviews of these interventions becomes problematic and impacts negatively on the ability of researchers and clinicians to formulate robust clinical guidelines for the radical treatment of gastric cancer.

This problem is not confined to previously reported trials. Our review of active trial protocols has demonstrated similar issues and further highlights the potential 'research waste' within this field. Glasziou and Chambers' estimate that 85 per cent of all biomedical research is 'wasted', and that a significant proportion of this can be attributed to problems choosing and reporting relevant outcomes in trials[78]. Thus, if we combine the number of patients who have participated in gastric cancer surgery trials over the last two decades with those that actively recruiting trials wish to attract, a total of twenty thousand patients may have participated in trials which, from a methodological perspective, could have had a far greater benefit and impact. Not only does this represent an inefficient use of time and scarce financial resource, but it may also have a longer-lasting negative impact on future trial participation by patients. Similar issues related to outcome reporting have been identified by several other groups supporting the theory that this is a widespread problem[79–82].

Furthermore, if the methodology of a particular trial is not sufficiently robust or the outcomes reported are not relevant to key stakeholders, the natural course will be for other researchers to examine the same interventions again, using a different approach. If these subsequent trials do not address the underlying methodological issues, they only contribute to a perpetual cycle which serves to weaken the evidence base. This is reflected within the field of gastric cancer surgery where thirteen trials have examined minimally invasive gastrectomy and a further 13 are actively recruiting to trials examining the same intervention.

Reported outcomes should be relevant to key stakeholders *including* patients. Given the sheer volume of complications that are reported by gastric surgical trials, one may expect to find the impact on QoL

For peer review only - http://bmjopen.bmj.com/site/about/guidelines.xhtml

is routinely reported. Indeed, QoL has been demonstrated as an important outcome to measure in other gastrointestinal cancer fields[83]. This has certainly not been the case with gastric cancer surgery trials over the last two decades and whilst there seems to be a greater acceptance by trials currently in recruitment that QoL is important to measure (although this group still represents less than half of ongoing trials), there remains great variation in relation to 'how' and 'when' it is measured.

To address these inconsistencies, we believe that a 'core outcome set' (COS) is required for gastric cancer surgery studies. Developing a minimum reporting standard will significantly contribute to getting the most out of randomized control trials which are expensive, labour intensive and logistically challenging to set up. A COS does not aim to restrict the outcomes that are reported, but merely to ensure that *the most critical outcomes* (as decided by key stakeholders) are clearly defined and measured uniformly.

One of the strengths of this study is that it is based on a reproducible and transparent methodology which has been subjected to critical appraisal from a study management team and peer-review process; a protocol of the GASTROS study which aims to develop this COS has been published previously[4]. Nonetheless, there are limitations. Including non-English and non-randomized studies in our search strategy may have yielded a greater number of outcomes and may have identified further 'outcomes'. However, when finalising our inclusion criteria, the two primary objectives of this review were considered - namely a) to describe the current landscape of outcome reporting in gastric cancer surgery RCTs and b) to take forward a 'long-list' of outcomes to be prioritised (by means of a Delphi survey) to form the basis of a COS for RCTs. Whilst we accept that such a COS would have benefits to non-RCTs and national audits, our primary focus was to improve the quality of RCTs and hence excluding other study types. In addition, there will be an opportunity during the Delphi survey (stage 2 of the GASTROS study) for participants to add further outcomes (not already identified from this review) which key stakeholders deem important to be considered for prioritisation.

In summary, the reporting of outcomes in gastric cancer surgery trials is inconsistent and there is large variation with respect to definitions, measurement tools and timing of measurement. This means that research and audit data cannot be synthesized efficiently. We believe that a COS to define a minimum

For peer review only - http://bmjopen.bmj.com/site/about/guidelines.xhtml

set of standards to implement across all gastric surgical trial is warranted. Including patients in the selection of the COS is important.

For peer review only

For peer review only - http://bmjopen.bmj.com/site/about/guidelines.xhtml

Declarations

Ethics Approval and Consent to Participate

Consent for Publication

Not applicable.

Availability of Data and Materials

Upon the completion of the study, supporting data will be available upon request.

Competing interests

The authors declare that they have no competing interests.

Funding

This study is funded by the National Institute for Health Research (NIHR) Doctoral Research Fellowship Grant programme (DRF-2015-08-023). The funding body has no role in the study design and implementation, writing the report, or decision to submit the report for publication.

Authors' contributions

BA contributed to the study design and drafted the manuscript. AMG, JMB, PRW and IAB contributed to the study design and revised the manuscript. All authors read and approved the final manuscript.

Authors' Information

Not applicable.

Acknowledgements

The authors would like to thank the patient representatives on the GASTROS Study Advisory Group for their continued advice and invaluable contribution to this study.

For peer review only

References

1. Fact Sheets by Cancer. http://globocan.iarc.fr/Pages/fact_sheets_cancer.aspx. Accessed July 24, 2017.

2. Quaresma M, Coleman MP, Rachet B. 40-year trends in an index of survival for all cancers combined and survival adjusted for age and sex for each cancer in England and Wales, 1971–2011: a population-based study. *Lancet*. 2015;385(9974):1206-1218. doi:10.1016/S0140-6736(14)61396-9.

3. Williamson PR, Altman DG, Bagley H, et al. The COMET Handbook: version 1.0. *Trials*. 2017;18(S3):280. doi:10.1186/s13063-017-1978-4.

4. Alkhaffaf B, Glenny A-M, Blazeby JM, Williamson P, Bruce IA. Standardising the Reporting of Outcomes in Gastric Cancer Surgery Trials: Protocol for the Development of a Core Outcome Set and Accompanying Outcome Measurement Instrument Set (The GASTROS Study). *Trials*. 2017;18(1):370. doi:10.1186/s13063-017-2100-7.

5. McCulloch P. Randomised trials in surgery: problems and possible solutions. *BMJ*. 2002;324(7351):1448-1451. doi:10.1136/bmj.324.7351.1448.

6. Hasegawa S, Yamamoto Y, Taguri M, et al. A Randomized Phase II Trial of Omentum-preserving Gastrectomy for Advanced Gastric Cancer. doi:10.1093/jjco/hys208.

7. Nakamura K, Katai H, Mizusawa J, et al. A Phase III Study of Laparoscopy-assisted Versus Open Distal Gastrectomy with Nodal Dissection for Clinical Stage IA/IB Gastric Cancer (JCOG0912). *Jpn J Clin Oncol*. 2013;43(3):324-327. doi:10.1093/jjco/hys220.

8. Yoshikawa T, Fukunaga T, Taguri M, et al. Laparoscopic or Open Distal Gastrectomy After Neoadjuvant Chemotherapy for Operable Gastric Cancer, a Randomized Phase II Trial (LANDSCOPE Trial). *Jpn J Clin Oncol*. 2012;42(7):654-657. doi:10.1093/jjco/hys057.

9. Effect of Laparoscopy-Assisted Distal Gastrectomy for Locally Advanced Gastric Cancer - Full Text View - ClinicalTrials.gov. https://clinicaltrials.gov/ct2/show/NCT02464215.

For peer review only - http://bmjopen.bmj.com/site/about/guidelines.xhtml

Accessed July 25, 2017.

10. The Long-term Effect of D4 Lymphadenectomy for Gastric Cancer - Full Text View - ClinicalTrials.gov. https://clinicaltrials.gov/ct2/show/NCT02423278. Accessed July 25, 2017.

11. A Comparison of Laparoscopic With Open Distal Gastrectomy in Advanced Gastric Cancer After Neoadjuvant Chemotherapy - Full Text View - ClinicalTrials.gov. https://clinicaltrials.gov/ct2/show/NCT02404753. Accessed July 25, 2017.

12. Randomized Controlled Trials Comparing Clinical Outcomes of 3D Versus 2D Laparoscopic Surgery for Gastric Cancer - Full Text View - ClinicalTrials.gov. https://clinicaltrials.gov/ct2/show/NCT02327481. Accessed July 25, 2017.

13. Prospective Study on Modified Delta-shaped Gastroduodenostomy in Totally Laparoscopic Surgery for Distal Gastric Cancer - Full Text View - ClinicalTrials.gov. https://clinicaltrials.gov/ct2/show/NCT02289183. Accessed July 25, 2017.

14. Haverkamp L, Brenkman HJF, Seesing MFJ, et al. Laparoscopic versus open gastrectomy for gastric cancer, a multicenter prospectively randomized controlled trial (LOGICA-trial). *BMC Cancer*. 2015;15:556. doi:10.1186/s12885-015-1551-z.

15. Laparoscopy-assisted and Open Distal Gastrectomy for Gastric Cancer in the Elderly Patients. https://clinicaltrials.gov/ct2/show/NCT02246153.

16. A Clinical Trial of D1+ Versus D2 Distal Gastrectomy for Stage IB & II Advanced Gastric Cancer - Full Text View - ClinicalTrials.gov. https://clinicaltrials.gov/ct2/show/NCT02144727. Accessed July 25, 2017.

17. Straatman J, van der Wielen N, Cuesta MA, et al. Surgical techniques, open versus minimally invasive gastrectomy after chemotherapy (STOMACH trial): study protocol for a randomized controlled trial. *Trials*. 2015;16(1):123. doi:10.1186/s13063-015-0638-9.

18. D2 and D2+ Radical Surgery for the Treatment of Advanced Distal Gastric Cancer - Full Text View - ClinicalTrials.gov. https://clinicaltrials.gov/ct2/show/NCT01836991. Accessed July 25,

For peer review only

For peer review only - http://bmjopen.bmj.com/site/about/guidelines.xhtml

2017.

19. Park JY, Kim Y-W, Ryu KW, et al. Assessment of laparoscopic stomach preserving surgery with sentinel basin dissection versus standard gastrectomy with lymphadenectomy in early gastric cancer–A multicenter randomized phase III clinical trial (SENORITA trial) protocol. *BMC Cancer*. 2016;16(1):340. doi:10.1186/s12885-016-2336-8.

20. Laparoscopy-assisted Total Gastrectomy for Clinical Stage I Gastric Cancer (KLASS-03). https://clinicaltrials.gov/ct2/show/study/NCT01584336. Accessed July 25, 2017.

21. Efficacy of Laparoscopic Subtotal Gastrectomy With D2 Lymph Node Dissection for Locally Advanced Gastric Cancer (KLASS-02-RCT). https://clinicaltrials.gov/ct2/show/NCT01456598.

22. GCSSG-SPNX: Trial to Evaluate Splenectomy in Total Gastrectomy for Proximal Gastric Carcinoma: JCOG0110 - Full Text View - ClinicalTrials.gov. https://clinicaltrials.gov/ct2/show/NCT00112099. Accessed July 25, 2017.

23. Randomized Controlled Trial to Evaluate Laparoscopic versus Open Surgery for Advanced Gastric Cancer (JLSSG0901: Adv.GC-LAP/OPEN, PII/III). http://apps.who.int/trialsearch/Trial2.aspx?TrialID=JPRN-UMIN000003420. Accessed July 25, 2017.

24. Comparsion of D2 verus D3 surgical procedure outcome in locally advanced gastric cancer patients following perioperative chemotherapy. http://ctri.nic.in/Clinicaltrials/pmaindet2.php?trialid=6992&EncHid=&userName=CTRI/2013/08/003882. Accessed July 25, 2017.

25. Prospective clinical trial on the long-term outcome and survival of vagus (coeliac branch)-preserving gastrectomy for cT1-3 gastric cancer. http://www.chictr.org.cn/showprojen.aspx?proj=4360. Accessed July 25, 2017.

26. Prospective clinical trial on the long-term outcome and survival of pylorus-preserving

For peer review only - http://bmjopen.bmj.com/site/about/guidelines.xhtml

gastrectomy for early gastric cancer. http://www.chictr.org.cn/showprojen.aspx?proj=4361. Accessed July 25, 2017.

27. Prospective and open multicenter randomize control study for enlarged lymphadenectomy of gastric cancer. http://www.chictr.org.cn/showprojen.aspx?proj=7870. Accessed July 25, 2017.

28. Open vs minimal invasive surgery for gastric submucosal tumor: a randomized trial on efficacy and safety. http://www.chictr.org.cn/showprojen.aspx?proj=4811. Accessed July 25, 2017.

29. A Multicenter Clinical Trial on Laparoscopic Gastric Cancer Surgery Compared With Open Surgery - Full Text View - ClinicalTrials.gov. https://clinicaltrials.gov/ct2/show/NCT02302794. Accessed July 25, 2017.

30. Hartgrink HH. Extended Lymph Node Dissection for Gastric Cancer: Who May Benefit? Final Results of the Randomized Dutch Gastric Cancer Group Trial. *J Clin Oncol*. 2004;22(11):2069-2077. doi:10.1200/JCO.2004.08.026.

31. Bonenkamp JJ, Songun I, Welvaart K, et al. Randomised comparison of morbidity after D1 and D2 dissection for gastric cancer in 996 Dutch patients. *Lancet*. 1995;345(8952):745-748. doi:10.1016/S0140-6736(95)90637-1.

32. Sasako M. Risk factors for surgical treatment in the Dutch Gastric Cancer Trial. *Br J Surg*. 1997;84(11):1567-1571.

33. Songun I, Putter H, Kranenbarg EM-K, Sasako M, van de Velde CJH. Surgical treatment of gastric cancer: 15-year follow-up results of the randomised nationwide Dutch D1D2 trial. *Lancet Oncol*. 2010;11(5):439-449. doi:10.1016/S1470-2045(10)70070-X.

34. Cuschieri a, Weeden S, Fielding J, et al. Patient survival after D1 and D2 resections for gastric cancer: long-term results of the MRC randomized surgical trial. Surgical Co-operative Group. *Br J Cancer*. 1999;79(9-10):1522-1530. doi:10.1016/S0959-8049(01)80998-9.

35. Cuschieri a, Fayers P, Fielding J, et al. Postoperative morbidity and mortality after D1 and D2

For peer review only - http://bmjopen.bmj.com/site/about/guidelines.xhtml

resections for gastric cancer: preliminary results of the MRC randomised controlled surgical trial. The Surgical Cooperative Group. *Lancet*. 1996;347:995-999. doi:10.1016/S0959-8049(01)80998-9.

36. Marubini E, Bozzetti F, Miceli R, Bonfanti G, Gennari L. Lymphadenectomy in gastric cancer: prognostic role and therapeutic implications. *Eur J Surg Oncol*. 2002;28(4):406-412. doi:Clinical Trial Comparative Study Randomized Controlled Trial Research Support, Non-U.S. Gov't.

37. Bozzetti F, Marubini E, Bonfanti G, Miceli R, Piano C, Gennari L. Subtotal versus total gastrectomy for gastric cancer: five-year survival rates in a multicenter randomized Italian trial. Italian Gastrointestinal Tumor Study Group. *Ann Surg*. 1999;230(2):170-178. doi:10.1097/00000658-199908000-00006.

38. Maeta M, Yamashiro H, Saito H, et al. A prospective pilot study of extended (D3) and superextended para-aortic lymphadenectomy (D4) in patients with T3 or T4 gastric cancer managed by total gastrectomy. *Surgery*. 1999;125(3):325-331. doi:10.1016/S0039-6060(99)70244-8.

39. Furukawa H, Hiratsuka M, Ishikawa O, et al. Total gastrectomy with dissection of lymph nodes along the splenic artery: a pancreas-preserving method. *Ann Surg Oncol*. 2000;7(9):669-673.

40. Csendes A, Burdiles P, Rojas J, Braghetto I, Diaz JC, Maluenda F. A prospective randomized study comparing D2 total gastrectomy versus D2 total gastrectomy plus splenectomy in 187 patients with gastric carcinoma. *Surgery*. 2002;131(4):401-407. doi:10.1067/msy.2002.121891.

41. Kitano S, Shiraishi N, Fujii K, Yasuda K, Inomata M, Adachi Y. A randomized controlled trial comparing open vs laparoscopy-assisted distal gastrectomy for the treatment of early gastric cancer: an interim report. *Surgery*. 2002;131(1 Suppl):0-11. doi:a120115 [pii].

For peer review only - http://bmjopen.bmj.com/site/about/guidelines.xhtml

42. Fujii K, Sonoda K, Izumi K, Shiraishi N, Adachi Y, Kitano S. T lymphocyte subsets and Th1/Th2 balance after laparoscopy-assisted distal gastrectomy. *Surg Endosc Other Interv Tech*. 2003;17(9):1440-1444. doi:10.1007/s00464-002-9149-3.

43. Degiuli M, Sasako M, Calgaro M, et al. Morbidity and mortality after D1 and D2 gastrectomy for cancer: interim analysis of the Italian Gastric Cancer Study Group (IGCSG) randomised surgical trial. *Eur J Surg Oncol*. 2004;30(3):303-308. doi:10.1016/j.ejso.2003.11.020.

44. Degiuli M, Sasako M, Ponti a. Morbidity and mortality in the italian gastric cancer study group randomized clinical trial of D1 versus D2 resection for gastric cancer. *Br J Surg*. 2010;97(5):643-649. doi:10.1002/bjs.6936.

45. Degiuli M, Sasako M, Ponti A, et al. Randomized clinical trial comparing survival after D1 or D2 gastrectomy for gastric cancer. *Br J Surg*. 2014;101(2):23-31. doi:10.1002/bjs.9345.

46. Sasako M, Sano T, Yamamoto S, et al. D2 lymphadenectomy alone or with para-aortic nodal dissection for gastric cancer. *N Engl J Med*. 2008;359(5):453-462. doi:10.1056/NEJMoa0707035.

47. Takeshi Sano. Gastric Cancer Surgery : Morbidity and Mortality Results From a Prospective Randomized Controlled Trial Comparing D2 and Extended Para-Aortic Lymphadenectomy — Japan Clinical Oncology Group Study 9501. *J Clin Oncol*. 2004;22(14):2767-2773. doi:10.1200/JCO.2004.10.184.

48. Kodera Y, Sasako M, Yamamoto S, Sano T, Nashimoto a, Kurita a. Identification of risk factors for the development of complications following extended and superextended lymphadenectomies for gastric cancer. *Br J Surg*. 2005;92(9):1103-1109. doi:10.1002/bjs.4979.

49. Tsujinaka T, Sasako M, Yamamoto S, et al. Influence of overweight on surgical complications for gastric cancer: results from a randomized control trial comparing D2 and extended para-aortic D3 lymphadenectomy (JCOG9501). *Ann Surg Oncol*. 2007;14(2):355-361.

For peer review only - http://bmjopen.bmj.com/site/about/guidelines.xhtml

doi:10.1245/s10434-006-9209-3.

50. Shibata C, Shiiba K, Funayama Y, et al. Outcomes after Pylorus-preserving Gastrectomy for Early Gastric Cancer: A Prospective Multicenter Trial. *World J Surg*. 2004;28(9):857-861. doi:10.1007/s00268-004-7369-5.

51. Inaba T, Okinaga K, Fukushima R, et al. Prospective randomized study of two laparotomy incisions for gastrectomy: Midline incision versus transverse incision. *Gastric Cancer*. 2004;7(3):167-171. doi:10.1007/s10120-004-0291-6.

52. Lee JH, Han HS. A prospective randomized study comparing open vs laparoscopy-assisted distal gastrectomy in early gastric cancer: early results. *Surg Endosc*. 2005;19(2):168-173. doi:10.1007/s00464-004-8808-y.

53. Hayashi H, Ochiai T, Shimada H, Gunji Y. Prospective randomized study of open versus laparoscopy-assisted distal gastrectomy with extraperigastric lymph node dissection for early gastric cancer. *Surg Endosc*. 2005;19(9):1172-1176. doi:10.1007/s00464-004-8207-4.

54. Huscher CGSS, Mingoli A, Sgarzini G, et al. Laparoscopic versus open subtotal gastrectomy for distal gastric cancer: five-year results of a randomized prospective trial. *Ann Surg*. 2005;241(2):232-237. doi:10.1097/01.sla.0000151892.35922.f2.

55. Wu C-W, Hsiung C a, Lo S-S, et al. Nodal dissection for patients with gastric cancer: a randomised controlled trial. *Lancet Oncol*. 2006;7(4):309-315. doi:10.1016/S1470-2045(06)70623-4.

56. Wu C-W, Chiou J-M, Ko F-S, et al. Quality of life after curative gastrectomy for gastric cancer in a randomised controlled trial. *Br J Cancer*. 2008;98(1):54-59. doi:10.1038/sj.bjc.6604097.

57. Wu CW, Hsiung C a., Lo SS, Hsieh MC, Shia LT, Whang-Peng J. Randomized clinical trial of morbidity after D1 and D3 surgery for gastric cancer. *Br J Surg*. 2004;91(3):283-287. doi:10.1002/bjs.4433.

58. Wu C-W, Chang I-S, Lo S, et al. Complications following D3 gastrectomy: post hoc analysis

For peer review only - http://bmjopen.bmj.com/site/about/guidelines.xhtml

of a randomized trial. *World J Surg*. 2006;30(1):12-16. doi:10.1007/s00268-005-7951-5.

59. Sasako M, Sano T, Yamamoto S, et al. Left thoracoabdominal approach versus abdominal-transhiatal approach for gastric cancer of the cardia or subcardia: a randomised controlled trial. *Lancet Oncol*. 2006;7(8):644-651. doi:10.1016/S1470-2045(06)70766-5.

60. Kurokawa Y, Sasako M, Sano T, et al. Ten-year follow-up results of a randomized clinical trial comparing left thoracoabdominal and abdominal transhiatal approaches to total gastrectomy for adenocarcinoma of the oesophagogastric junction or gastric cardia. *Br J Surg*. 2015;102(4):341-348. doi:10.1002/bjs.9764.

61. Yu W, Choi GS, Chung HY. Randomized clinical trial of splenectomy versus splenic preservation in patients with proximal gastric cancer. *Br J Surg*. 2006;93(5):559-563. doi:10.1002/bjs.5353.

62. Kulig J, Popiela T, Kolodziejczyk P, Sierzega M, Szczepanik A. Standard D2 versus extended D2 (D2+) lymphadenectomy for gastric cancer: an interim safety analysis of a multicenter, randomized, clinical trial. *Am J Surg*. 2007;193(1):10-15. doi:10.1016/j.amjsurg.2006.04.018.

63. Kim YW, Baik YH, Yun YH, et al. Improved quality of life outcomes after laparoscopy-assisted distal gastrectomy for early gastric cancer: results of a prospective randomized clinical trial. *Ann Surg*. 2008;248(5):721-727. doi:10.1097/SLA.0b013e318185e62e\r00000658-200811000-00006 [pii].

64. Kim YW, Yoon HM, Yun YH, et al. Long-term outcomes of laparoscopy-assisted distal gastrectomy for early gastric cancer: Result of a randomized controlled trial (COACT 0301). *Surg Endosc Other Interv Tech*. 2013;27(11):4267-4276. doi:10.1007/s00464-013-3037-x.

65. Yonemura Y, Wu CC, Fukushima N, et al. Randomized clinical trial of D2 and extended paraaortic lymphadenectomy in patients with gastric cancer. *Int J Clin Oncol*. 2008;13(2):132-137. doi:10.1007/s10147-007-0727-1.

66. Kim W, Kim H-H, Han S-U, et al. Decreased Morbidity of Laparoscopic Distal Gastrectomy

For peer review only - http://bmjopen.bmj.com/site/about/guidelines.xhtml

Compared With Open Distal Gastrectomy for Stage I Gastric Cancer. *Ann Surg*. 2015;XX(X):1. doi:10.1097/SLA.0000000000001346.

67. Kim H-H, Hyung WJ, Cho GS, et al. Morbidity and mortality of laparoscopic gastrectomy versus open gastrectomy for gastric cancer: an interim report--a phase III multicenter, prospective, randomized Trial (KLASS Trial). *Ann Surg*. 2010;251(3):417-420. doi:10.1097/SLA.0b013e3181cc8f6b.

68. Imamura H, Kurokawa Y, Kawada J, et al. Influence of bursectomy on operative morbidity and mortality after radical gastrectomy for gastric cancer: Results of a randomized controlled trial. *World J Surg*. 2011;35(3):625-630. doi:10.1007/s00268-010-0914-5.

69. Cai J, Wei D, Gao CF, Zhang CS, Zhang H, Zhao T. A prospective randomized study comparing open versus laparoscopy-assisted D2 radical gastrectomy in advanced gastric cancer. *Dig Surg*. 2012;28(5-6):331-337. doi:10.1159/000330782.

70. Chen Hu J, Xin Jiang L, Cai L, et al. Preliminary experience of fast-track surgery combined with laparoscopy-assisted radical distal gastrectomy for gastric cancer. *J Gastrointest Surg*. 2012;16(10):1830-1839. doi:10.1007/s11605-012-1969-4.

71. Takiguchi S, Fujiwara Y, Yamasaki M. Laparoscopy-Assisted Distal Gastrectomy Versus Open Distal. *World J Surg*. 2013;37(10):2379-2386. doi:10.1007/s00268-013-2121-7.

72. Lee J-H, Son S-Y, Lee CM, Ahn SH, Park DJ, Kim H-H. Morbidity and mortality after laparoscopic gastrectomy for advanced gastric cancer: Results of a phase II clinical trial. *Surg Endosc Other Interv Tech*. 2013;27(8):2877-2885. doi:10.1007/s00464-013-2848-0.

73. Sakuramoto S, Yamashita K, Kikuchi S, et al. Laparoscopy versus open distal gastrectomy by expert surgeons for early gastric cancer in Japanese patients: short-term clinical outcomes of a randomized clinical trial. *Surg Endosc*. 2013;27(5):1695-1705. doi:10.1007/s00464-012-2658-9.

74. Aoyama T, Yoshikawa T, Hayashi T, et al. Randomized comparison of surgical stress and the

For peer review only - http://bmjopen.bmj.com/site/about/guidelines.xhtml

For peer review only

nutritional status between laparoscopy-assisted and open distal gastrectomy for gastric cancer. *Ann Surg Oncol*. 2014;21(6):1983-1990. doi:10.1245/s10434-014-3509-9.

75. Hirao M, Kurokawa Y, Fujita J, et al. Long-term outcomes after prophylactic bursectomy in patients with resectable gastric cancer: Final analysis of a multicenter randomized controlled trial. *Surgery*. 2015;157(6):1099-1105. doi:10.1016/j.surg.2014.12.024.

76. Galizia G, Lieto E, De Vita F, et al. Modified versus standard D2 lymphadenectomy in total gastrectomy for nonjunctional gastric carcinoma with lymph node metastasis. *Surg (United States)*. 2015;157(2):285-296. doi:10.1016/j.surg.2014.09.012.

77. Hu Y, Huang C, Sun Y, et al. Morbidity and Mortality of Laparoscopic Versus Open D2 Distal Gastrectomy for Advanced Gastric Cancer: A Randomized Controlled Trial. *J Clin Oncol*. 2016. doi:10.1200/JCO.2015.63.7215.

78. Chalmers I, Glasziou P. Avoidable waste in the production and reporting of research evidence. *Lancet (London, England)*. 2009;374(9683):86-89. doi:10.1016/S0140-6736(09)60329-9.

79. Blencowe NS, Strong S, McNair AGK, et al. Reporting of short-term clinical outcomes after esophagectomy: a systematic review. *Ann Surg*. 2012;255(4):658-666. doi:10.1097/SLA.0b013e3182480a6a.

80. Currie A, Brigic A, Blencowe NS, et al. Systematic review of surgical innovation reporting in laparoendoscopic colonic polyp resection. *Br J Surg*. 2015;102(2):e108-e116. doi:10.1002/bjs.9675.

81. Hopkins JC, Howes N, Chalmers K, et al. Outcome reporting in bariatric surgery: an in-depth analysis to inform the development of a core outcome set, the BARIACT Study. *Obes Rev*. 2015;16(1):88-106. doi:10.1111/obr.12240.

82. Whistance RN, Forsythe RO, McNair AGK, et al. A systematic review of outcome reporting in colorectal cancer surgery. *Colorectal Dis*. 2013;15(10):e548-60. doi:10.1111/codi.12378.

83. Avery KNL, Chalmers KA, Brookes ST, et al. Development of a Core Outcome Set for

For peer review only - http://bmjopen.bmj.com/site/about/guidelines.xhtml

For peer review only

Clinical Effectiveness Trials in Esophageal Cancer Resection Surgery. *Ann Surg*. March 2017:1. doi:10.1097/SLA.0000000000002204.

For peer review only

For peer review only - http://bmjopen.bmj.com/site/about/guidelines.xhtml

Figure Legends

Figure 1. Study selection and inclusion.

Figure 2. Outcome themes reported in a) gastric cancer surgery trials and b) proposed outcome themes to be reported in future trials based on study protocols.

For peer review only

For peer review only - http://bmjopen.bmj.com/site/about/guidelines.xhtml

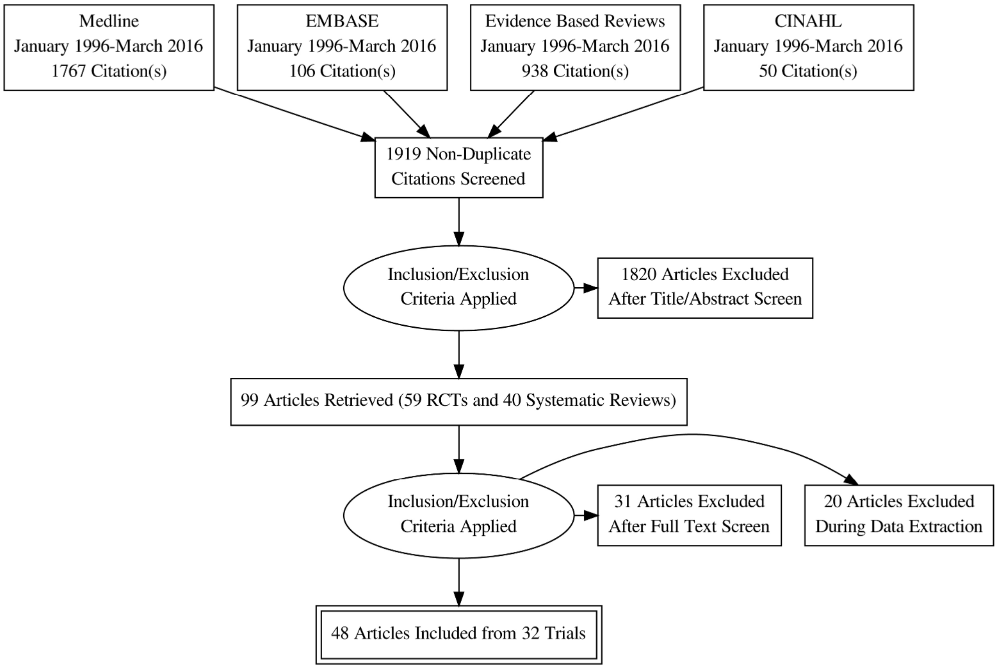

Figure 1. Study selection and inclusion.

269x180mm (300 x 300 DPI)

Figure 2. Outcome themes reported in a) gastric cancer surgery trials and b) proposed outcome themes to be reported in future trials based on study protocols.

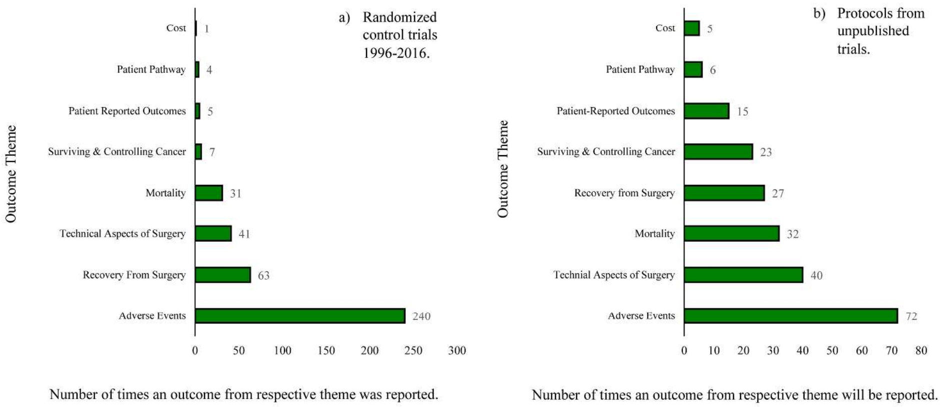

Figure 2. Outcome themes reported in a) gastric cancer surgery trials and b) proposed outcome themes to be reported in future trials based on study protocols.

264x126mm (300 x 300 DPI)

Appendix 1. Outcomes reported in 48 publications from 32 gastric cancer surgery trials.

Theme	Original Outcome	Frequency Reported
Adverse Events	Abdominal abscess	10
Adverse Events	Abdominal distention	1
Adverse Events	Abdominal drainage	1
Adverse Events	Abdominal liquid accumulation	1
Adverse Events	Abscess intra-abdominal	7
Adverse Events	Abscess subphrenic	1
Adverse Events	Acute enteritis	1
Adverse Events	Acute urinary retention	1
Adverse Events	Adverse drug reaction	1
Adverse Events	Afferent loop syndrome	2
Adverse Events	Allogenic blood transfusion	1
Adverse Events	Amylase level in drainage fluid	1
Adverse Events	Anastomosis failure	1
Adverse Events	Anastomosis stricture	2
Adverse Events	Anastomotic bleeding	1
Adverse Events	Anastomotic dehiscence	2
Adverse Events	Anastomotic leak	17
Adverse Events	Anastomotic leakage from GJ	1
Adverse Events	Anastomotic leakage from OJ	1
Adverse Events	Anastomotic leakage type 1	1
Adverse Events	Anastomotic leakage type 2	1
Adverse Events	Anastomotic stenosis	5
Adverse Events	Any complication	2
Adverse Events	ARDS	1

For peer review only

Adverse Events	Arteriosclerosis obliterans of the leg	1
Adverse Events	Ascites	2
Adverse Events	Atelectasis	2
Adverse Events	Atelectasis or pleural effusion	1
Adverse Events	Atrial fibrillation	1
Adverse Events	Bleeding	4
Adverse Events	Bleeding abdominal	1
Adverse Events	Bleeding from anastomosis	1
Adverse Events	Blood transfusion	2
Adverse Events	Blood transfusion volume	2
Adverse Events	Body temperature exceeding 37 ° C (days)	2
Adverse Events	Bowel obstruction	1
Adverse Events	Bowel obstruction/ileus	1
Adverse Events	Bronchopneumonia	1
Adverse Events	Bronchoscopic toilet	1
Adverse Events	Cardiac complications	6
Adverse Events	Cardiac failure	1
Adverse Events	Cardiocirculatory	1
Adverse Events	Cardiopulmonary disaese	1
Adverse Events	Catheter-induced sepsis	1
Adverse Events	Cerebrovascular	1
Adverse Events	Cholecystitis	3
Adverse Events	Cholecystitis acute	1
Adverse Events	Cholecystitis requiring percutaneous drainage	1
Adverse Events	Chyle leakage	2
Adverse Events	Chylous drainage	1
Adverse Events	Chylous lymphorrhea	1

For peer review only

Adverse Events	Colonic perforation	1
Adverse Events	Complications	3
Adverse Events	Complications after discharge	1
Adverse Events	Complications number of	1
Adverse Events	Deep vein thrombosis	3
Adverse Events	Delayed gastric emptying	2
Adverse Events	Delayed gastric emptying without obstruction	1
Adverse Events	Diarrhoea	1
Adverse Events	Drug-induced hepatitis	1
Adverse Events	Dumping syndrome	1
Adverse Events	Duodenal leak	1
Adverse Events	Duodenal stump leak	3
Adverse Events	Early dumping syndrome	1
Adverse Events	Early surgical complications	1
Adverse Events	Empyema thoracis	1
Adverse Events	Endocrine complications	1
Adverse Events	Endocrine events	1
Adverse Events	Enterocutaneous fistula	3
Adverse Events	Esophagus and remnant stomach infarction	1
Adverse Events	Fever	1
Adverse Events	Fluid collection	1
Adverse Events	Fluid collection/abscesses	1
Adverse Events	Gastric atonia	1
Adverse Events	Gastric remnant necrosis	1
Adverse Events	Gastrointestinal bleeding	1
Adverse Events	Gastrointestinal complications	1
Adverse Events	Gastrointestinal injury	1

For peer review only

Adverse Events	Gastroparesis	3
Adverse Events	Haemorrhage	2
Adverse Events	Hb	2
Adverse Events	Hepatic complications	2
Adverse Events	Hepatic failure	1
Adverse Events	Herpes zoster	1
Adverse Events	Hiccups	1
Adverse Events	Hospital morbidity	1
Adverse Events	Hypercapnia	1
Adverse Events	Iatrogenic spleen injury	1
Adverse Events	Idiopathic small bowel perforation	1
Adverse Events	Iileus mechanical	1
Adverse Events	Ileus	2
Adverse Events	Ileus adhesive	2
Adverse Events	Ileus paralytic	1
Adverse Events	Ileus prolonged	1
Adverse Events	Incision fat liquefaction	1
Adverse Events	Incision infection	1
Adverse Events	Infection	1
Adverse Events	Intestinal fistula	2
Adverse Events	Intestinal ischaemia	1
Adverse Events	Intestinal obstruction	3
Adverse Events	Intra-abdominal bleeding	3
Adverse Events	Intra-abdominal collections	1
Adverse Events	Intra-abdominal complications	1
Adverse Events	Intra-abdominal infection	1
Adverse Events	Intraluminal bleeding	3

For peer review only

Adverse Events	Intraoperative blood transfusion	2
Adverse Events	Intraoperative complications	3
Adverse Events	intraoperative major bleeding	1
Adverse Events	Intraperitoneal haemorrhage	1
Adverse Events	Late surgical complications	1
Adverse Events	Leakage	1
Adverse Events	Liver dysfunction	1
Adverse Events	Local complications	1
Adverse Events	Long-term complications	1
Adverse Events	Lung Infection	1
Adverse Events	Lymphatic leakage	1
Adverse Events	Lymphorrhoea	1
Adverse Events	Major abdominal infections	1
Adverse Events	Major cardiorespiratory incidents	1
Adverse Events	Major complications	2
Adverse Events	Major post-operative complication	1
Adverse Events	Major surgical complications	1
Adverse Events	Malabsorption	1
Adverse Events	Mediastinitis	1
Adverse Events	Medical complications	1
Adverse Events	Metabolic complications	1
Adverse Events	Minor complications	1
Adverse Events	Minor discharge of pancreatic juice	1
Adverse Events	Minor leakage	1
Adverse Events	Minor patchy pulmonary collapse	1
Adverse Events	Minor pulmonary atelectasis	2
Adverse Events	Morbidity	8

For peer review only

Adverse Events	Morbidity rate	1
Adverse Events	Multiple organ failure	1
Adverse Events	Myocardial infarction	1
Adverse Events	Nausea	1
Adverse Events	Need for blood transfusion	1
Adverse Events	Non-surgical Complications	6
Adverse Events	Number of patients with complications	1
Adverse Events	Operative complications	1
Adverse Events	Operative morbidity	3
Adverse Events	Other complications	7
Adverse Events	Overall complications	2
Adverse Events	Overall Post-operative complications	1
Adverse Events	Pancreas-related complications	1
Adverse Events	Pancreatic fistula	15
Adverse Events	Pancreatic injury	2
Adverse Events	Pancreatic leak	4
Adverse Events	Pancreatitis	1
Adverse Events	Pancreatitis acute	3
Adverse Events	Pancreatitis edematous	1
Adverse Events	Pancreatitis severe	1
Adverse Events	Pancreatitis traumatic	1
Adverse Events	Peri-operative complications	1
Adverse Events	Peritoneal haemorrhage	1
Adverse Events	Pleural	1
Adverse Events	Pleural effusion	5
Adverse Events	Pleural fluid	1
Adverse Events	Pneumonia	11

For peer review only

Adverse Events	Post-operative bleeding	1
Adverse Events	Post-operative complications	5
Adverse Events	Post-operative drain discharge	1
Adverse Events	Post-operative glucose tolerance	1
Adverse Events	Post-operative hemorrhage	2
Adverse Events	Post-operative major complications	1
Adverse Events	Post-operative minor complication	1
Adverse Events	Post-operative morbidity	1
Adverse Events	Post-operative psychosis	1
Adverse Events	Post-operative respiratory care	1
Adverse Events	Post-operative respiratory function	1
Adverse Events	Post-operative surgical parameters	1
Adverse Events	Post-operative symptoms	1
Adverse Events	Presence of gallstones	1
Adverse Events	Procedure-related morbidity and mortality	1
Adverse Events	Prolonged diarrhea	1
Adverse Events	Prolonged retention of intra-abdominal fluid	1
Adverse Events	Pulmonary	4
Adverse Events	Pulmonary complications	4
Adverse Events	Pulmonary edema	1
Adverse Events	Pulmonary embolism	2
Adverse Events	Pulmonary infection	1
Adverse Events	Pyothorax	1
Adverse Events	Rate of reinsertion of NG tube	1
Adverse Events	Recurrent laryngeal nerve palsy	1
Adverse Events	Reflux oesophagitis	2
Adverse Events	Re-laparotomy	2

For peer review only

Adverse Events	Renal complications	2
Adverse Events	Renal failure	2
Adverse Events	Re-operation	8
Adverse Events	Re-operation details	1
Adverse Events	Respirator use after surgery	1
Adverse Events	Respiratory complications	1
Adverse Events	Respiratory failure	1
Adverse Events	Return to theatre	1
Adverse Events	Septic complications	1
Adverse Events	Serious and potentially fatal complications	1
Adverse Events	Severe diarrhoea	1
Adverse Events	Severe feeding problem requiring prolonged hyperalimentation	1
Adverse Events	Severity of complications	2
Adverse Events	Severity of post-operative complications	1
Adverse Events	Short-term complications	1
Adverse Events	Small-bowel obstruction	1
Adverse Events	Splenic artery pseudoaneurysm	1
Adverse Events	Splenic injury	1
Adverse Events	Stasis	1
Adverse Events	Stenosis	1
Adverse Events	Surgical complications	8
Adverse Events	Surgical risk	1
Adverse Events	Systemic complications	1
Adverse Events	Systemic infections	1
Adverse Events	Thermal injury	1
Adverse Events	Thoracic effusion requiring thoracic drainage	1

For peer review only

Adverse Events	Thromboembolic complications	1
Adverse Events	Total complications	1
Adverse Events	Total major complications	1
Adverse Events	Total morbidity	4
Adverse Events	Tracheotomy	1
Adverse Events	Transfusion	3
Adverse Events	Transfusions received	1
Adverse Events	Transient ischemic attack	1
Adverse Events	Transient LFT abnormality	1
Adverse Events	Trocar related injury	1
Adverse Events	Tube tracheotomy	1
Adverse Events	Uncomplicated calf vein thrombosis	1
Adverse Events	Upper gastro-intestinal haemorrhage	1
Adverse Events	Urinary complications	2
Adverse Events	Urinary retention	1
Adverse Events	Urinary tract complications	1
Adverse Events	Urinary tract infection	3
Adverse Events	Viral infection	1
Adverse Events	Vomiting	1
Adverse Events	Wound abscess	1
Adverse Events	Wound complications	2
Adverse Events	Wound dehiscence	2
Adverse Events	Wound evisceration	1
Adverse Events	Wound haematoma	1
Adverse Events	Wound infection	10
Adverse Events	Wound infection/dehiscence	1
Adverse Events	Wound problem	1

For peer review only

Adverse Events	Wound seroma	2
Cost	Medical cost	1
Mortality	Death	8
Mortality	Death from a post-operative complication	1
Mortality	Death from all causes	1
Mortality	Death from gastric cancer as a cause	1
Mortality	Disease free survival	4
Mortality	Disease free survival 4-year	2
Mortality	Disease free survival 5-year	3
Mortality	Disease specific survival	1
Mortality	Disease specific survival 5-year	1
Mortality	Gastric cancer related deaths	1
Mortality	Hospital death	3
Mortality	Hospital mortality	7
Mortality	In-hospital mortality	2
Mortality	Mortality not related to surgery	1
Mortality	Operative death	1
Mortality	Operative mortality	3
Mortality	Overall survival	8
Mortality	Overall survival 10-year	1
Mortality	Overall survival 3-year	2
Mortality	Overall survival 5-year	6
Mortality	Overall survival 6-year	1
Mortality	Overall survival 7-year	1
Mortality	Post-operative death	4
Mortality	Post-operative mortality	3
Mortality	Post-operative survival	1

For peer review only

Mortality	Recurrence-free survival	5
Mortality	Relapse-free survival	1
Mortality	Survival 11-year	1
Mortality	Survival 5-year	11
Mortality	Survival Period	1
Mortality	Treatment related deaths	1
Patient Pathway	Days of hospitalization	1
Patient Pathway	Duration of hospital stay	12
Patient Pathway	Duration of post-operative hospital stay	11
Patient Pathway	Readmission	1
Patient Reported Outcomes	Degree of pain	1
Patient Reported Outcomes	Overall satisfaction	1
Patient Reported Outcomes	Pain	1
Patient Reported Outcomes	Post-operative Pain	1
Patient Reported Outcomes	QoL	3
Recovery From Surgery	4-day post-operative use of analgesics	1
Recovery From Surgery	Blood urea nitrogen	1
Recovery From Surgery	Body weight	1
Recovery From Surgery	CK	1
Recovery From Surgery	CRP	4
Recovery From Surgery	CRP 3 days after surgery	1
Recovery From Surgery	Days of fever	1
Recovery From Surgery	Days to sips of water	1
Recovery From Surgery	Decrease in body weight	1
Recovery From Surgery	Decrease of body weight 1 month after the operation	1
Recovery From Surgery	Dose of analgesic (mg)	1
Recovery From Surgery	Duration of pain control	1

For peer review only

Recovery From Surgery	Eating	1
Recovery From Surgery	FEV1(L)	1
Recovery From Surgery	FEVC(L)	1
Recovery From Surgery	Fever	1
Recovery From Surgery	First eating (post-operative day)	1
Recovery From Surgery	First walking (post-operative day)	1
Recovery From Surgery	Food intake	1
Recovery From Surgery	Frequency of analgesics injection	1
Recovery From Surgery	Frequency of injection given according to analgesic requests	1
Recovery From Surgery	IL-6	1
Recovery From Surgery	Immediate postoperative inflammatory and immune responses	1
Recovery From Surgery	Immunological response to surgery	2
Recovery From Surgery	Lean body mass	1
Recovery From Surgery	LFT	1
Recovery From Surgery	Number of days to get out of bed	1
Recovery From Surgery	Nutritional Status	1
Recovery From Surgery	Pain control	2
Recovery From Surgery	Post-operative analgesia	1
Recovery From Surgery	Post-operative course	1
Recovery From Surgery	Post-operative pain	1
Recovery From Surgery	Post-operative recovery	1
Recovery From Surgery	Prealbumin	1
Recovery From Surgery	Progression of oral intake	1
Recovery From Surgery	Pulmonary function	1
Recovery From Surgery	Recovery of Physical Activity	1
Recovery From Surgery	Residual pain at day 7	1

For peer review only

Recovery From Surgery	SaO2	1
Recovery From Surgery	Serum Albumin	1
Recovery From Surgery	Surgical stress response	1
Recovery From Surgery	The early recovery course	1
Recovery From Surgery	Time of first flatus/index of peristalsis recovery	1
Recovery From Surgery	Time to ambulation	1
Recovery From Surgery	Time to first flatus	4
Recovery From Surgery	Time to first flatus (days)	5
Recovery From Surgery	Time to first liquid intake	1
Recovery From Surgery	Time to first soft diet uptake	1
Recovery From Surgery	Time to food intake	1
Recovery From Surgery	Time to liquid diet	1
Recovery From Surgery	Time to removal of epidural anesthesia (days)	1
Recovery From Surgery	Time to sips of water	2
Recovery From Surgery	Time to start oral intake (days)	1
Recovery From Surgery	Time until removal of the naso-gastric tube	1
Recovery From Surgery	Time until start of meals	1
Recovery From Surgery	Times analgesic given	1
Recovery From Surgery	Times of pain rescue	1
Recovery From Surgery	Total amount of analgesics infused	1
Recovery From Surgery	Total body weight	1
Recovery From Surgery	Total protein	1
Recovery From Surgery	Walking	1
Recovery From Surgery	WBC	1
Recovery From Surgery	WCC	3
Surviving & Controlling Cancer	Cumulative risk of recurrence	2

For peer review only

Surviving & Controlling Cancer	Disease recurrence rate	1
Surviving & Controlling Cancer	Port site metastasis	2
Surviving & Controlling Cancer	Recurrence	4
Surviving & Controlling Cancer	Recurrence patterns	1
Surviving & Controlling Cancer	Recurrent disease	1
Surviving & Controlling Cancer	Tumor recurrence	1
Technical Aspects of Surgery	Amount of bleeding	1
Technical Aspects of Surgery	Blood loss	7
Technical Aspects of Surgery	Clear margin distance	1
Technical Aspects of Surgery	Conversion to open surgery	3
Technical Aspects of Surgery	Dissected Lymph nodes - mediastinal	1
Technical Aspects of Surgery	Dissected Lymph nodes - para-aortic	1
Technical Aspects of Surgery	Distal resection margin	2
Technical Aspects of Surgery	Duration of surgery	3
Technical Aspects of Surgery	Estimated blood loss	5
Technical Aspects of Surgery	Intraoperative blood loss	7
Technical Aspects of Surgery	Length of laparotomy incision	1
Technical Aspects of Surgery	Length of lesser curvature of resected stomach	1
Technical Aspects of Surgery	Length of lesser curvature of resected stomach	1
Technical Aspects of Surgery	Length of longest wound	1
Technical Aspects of Surgery	Length of resection on greater cureve	1

For peer review only

Technical Aspects of Surgery	Length of resection on lesser curve	1
Technical Aspects of Surgery	Main wound size	1
Technical Aspects of Surgery	Mean blood loss	2
Technical Aspects of Surgery	Mean operating time	1
Technical Aspects of Surgery	Number of lymph nodes dissected or resected or retrieved	22
Technical Aspects of Surgery	Number of lymph nodes removed N1 group	1
Technical Aspects of Surgery	Number of lymph nodes removed N2 group	1
Technical Aspects of Surgery	Number of lymph nodes removed N3 group	1
Technical Aspects of Surgery	Number of lymph nodes removed N4 group	1
Technical Aspects of Surgery	Operative blood loss	2
Technical Aspects of Surgery	Operative time	18
Technical Aspects of Surgery	Pathological outcomes	1
Technical Aspects of Surgery	Proximal margin positive/negative	1
Technical Aspects of Surgery	Proximal resection margin	2
Technical Aspects of Surgery	R0 resection	2
Technical Aspects of Surgery	Radicality R0	1
Technical Aspects of Surgery	Radicality R1	1
Technical Aspects of Surgery	Resection line involvement - distal	1
Technical Aspects of Surgery	Resection line involvement - proximal	1
Technical Aspects of Surgery	Residual Tumour	1
Technical Aspects of Surgery	Residual tumour R0	2
Technical Aspects of Surgery	Residual tumour R1/2	1
Technical Aspects of Surgery	Residual tumour R1/2	1
Technical Aspects of Surgery	Surgical time	2
Technical Aspects of Surgery	Time for operation	1
Technical Aspects of Surgery	Wound size	5

For peer review only

PRISMA 2009 Checklist

Section/topic	#	Checklist item	Reported on page #
**TITLE**			
Title	1	Identify the report as a systematic review, meta-analysis, or both.	1
**ABSTRACT**			
Structured summary	2	Provide a structured summary including, as applicable: background; objectives; data sources; study eligibility criteria, participants, and interventions; study appraisal and synthesis methods; results; limitations; conclusions and implications of key findings; systematic review registration number.	4
**INTRODUCTION**			
Rationale	3	Describe the rationale for the review in the context of what is already known.	6
Objectives	4	Provide an explicit statement of questions being addressed with reference to participants, interventions, comparisons, outcomes, and study design (PICOS).	6
**METHODS**			
Protocol and registration	5	Indicate if a review protocol exists, if and where it can be accessed (e.g., Web address), and, if available, provide registration information including registration number.	6
Eligibility criteria	6	Specify study characteristics (e.g., PICOS, length of follow-up) and report characteristics (e.g., years considered, language, publication status) used as criteria for eligibility, giving rationale.	8, table 1
Information sources	7	Describe all information sources (e.g., databases with dates of coverage, contact with study authors to identify additional studies) in the search and date last searched.	7, 8
Search	8	Present full electronic search strategy for at least one database, including any limits used, such that it could be repeated.	7
Study selection	9	State the process for selecting studies (i.e., screening, eligibility, included in systematic review, and, if applicable, included in the meta-analysis).	7
Data collection process	10	Describe method of data extraction from reports (e.g., piloted forms, independently, in duplicate) and any processes for obtaining and confirming data from investigators.	9
Data items	11	List and define all variables for which data were sought (e.g., PICOS, funding sources) and any assumptions and simplifications made.	9
Risk of bias in individual studies	12	Describe methods used for assessing risk of bias of individual studies (including specification of whether this was done at the study or outcome level), and how this information is to be used in any data synthesis.	n/a
Summary measures	13	State the principal summary measures (e.g., risk ratio, difference in means).	n/a
Synthesis of results	14	Describe the methods of handling data and combining results of studies, if done, including measures of consistency (e.g., I^2) for each meta-analysis.	n/a

For peer review only - http://bmjopen.bmj.com/site/about/guidelines.xhtml

PRISMA 2009 Checklist

Section/topic	#	Checklist item	Reported on page #
Risk of bias across studies	15	Specify any assessment of risk of bias that may affect the cumulative evidence (e.g., publication bias, selective reporting within studies).	n/a
Additional analyses	16	Describe methods of additional analyses (e.g., sensitivity or subgroup analyses, meta-regression), if done, indicating which were pre-specified.	n/a
**RESULTS**			
Study selection	17	Give numbers of studies screened, assessed for eligibility, and included in the review, with reasons for exclusions at each stage, ideally with a flow diagram.	Figure 1
Study characteristics	18	For each study, present characteristics for which data were extracted (e.g., study size, PICOS, follow-up period) and provide the citations.	Table 2
Risk of bias within studies	19	Present data on risk of bias of each study and, if available, any outcome level assessment (see item 12).	n/a
Results of individual studies	20	For all outcomes considered (benefits or harms), present, for each study: (a) simple summary data for each intervention group (b) effect estimates and confidence intervals, ideally with a forest plot.	n/a
Synthesis of results	21	Present results of each meta-analysis done, including confidence intervals and measures of consistency.	n/a
Risk of bias across studies	22	Present results of any assessment of risk of bias across studies (see Item 15).	n/a
Additional analysis	23	Give results of additional analyses, if done (e.g., sensitivity or subgroup analyses, meta-regression [see Item 16]).	n/a
**DISCUSSION**			
Summary of evidence	24	Summarize the main findings including the strength of evidence for each main outcome; consider their relevance to key groups (e.g., healthcare providers, users, and policy makers).	16
Limitations	25	Discuss limitations at study and outcome level (e.g., risk of bias), and at review-level (e.g., incomplete retrieval of identified research, reporting bias).	16
Conclusions	26	Provide a general interpretation of the results in the context of other evidence, and implications for future research.	16
**FUNDING**			
Funding	27	Describe sources of funding for the systematic review and other support (e.g., supply of data); role of funders for the systematic review.	2

From: Moher D, Liberati A, Tetzlaff J, Altman DG, The PRISMA Group (2009). Preferred Reporting Items for Systematic Reviews and Meta-Analyses: The PRISMA Statement. PLoS Med 6(7): e1000097. doi:10.1371/journal.pmed1000097

For more information, visit: **www.prisma-statement.org**.

For peer review only - http://bmjopen.bmj.com/site/about/guidelines.xhtml

BMJ Open

Reporting of Outcomes in Gastric Cancer Surgery Trials: A Systematic Review.

Journal:	*BMJ Open*
Manuscript ID	bmjopen-2018-021796.R1
Article Type:	Research
Date Submitted by the Author:	29-May-2018
Complete List of Authors:	Alkhaffaf, Bilal; Department of Oesophago-Gastric Surgery<br>Blazeby, Jane; University of Bristol, Centre for Surgical Research<br>Williamson, Paula; University of Liverpool<br>Bruce, Iain; Manchester University Hospitals NHS Foundation Trust, Paediatric ENT Department<br>Glenny, Anne-Marie; University of Manchester, Division of Dentistry
**Primary Subject Heading**:	Surgery
Secondary Subject Heading:	Oncology, Evidence based practice, Research methods, Patient-centred medicine
Keywords:	Surgical Oncology, Stomach Neoplasms, Outcome Assessment, Delphi Technique, Patient Reported Outcome Measures, Outcome reporting

SCHOLARONE™
Manuscripts

For peer review only

For peer review only - http://bmjopen.bmj.com/site/about/guidelines.xhtml

Title Page

Reporting of Outcomes in Gastric Cancer Surgery Trials: A Systematic Review.

Authors

Bilal Alkhaffaf[1,2], Jane M Blazeby[3], Paula R Williamson[4], Iain A Bruce[5,6] & Anne-Marie Glenny[7]

Affiliations

[1]Department of Oesophago-Gastric Surgery, Manchester University Hospitals NHS foundation Trust, Manchester Academic Health Science Centre, Manchester, UK

[2]Division of Molecular & Clinical Cancer, Faculty of Biology, Medicine and Health, University of Manchester

[3]Centre for Surgical Research, University of Bristol

[4]MRC North West Hub for Trials Methodology Research, University of Liverpool

[5]Paediatric ENT Department, Royal Manchester Children's Hospital, Manchester University Hospitals NHS Foundation Trust, Manchester Academic Health Science Centre, Manchester, UK

[6]Division of Infection, Immunity and Respiratory Medicine, Faculty of Biology, Medicine and Health, University of Manchester, Manchester, UK

[7]Division of Dentistry, University of Manchester

Corresponding Author

Mr Bilal Alkhaffaf

Consultant Oesophago-Gastric Surgeon

Manchester University Hospitals NHS Foundation Trust

Manchester Royal Infirmary

Oxford Road

For peer review only - http://bmjopen.bmj.com/site/about/guidelines.xhtml

M13 9WL

E-mail: bilal.alkhaffaf@mft.nhs.uk

Telephone: +44 161 276 1234

Manuscript date: 09/05/2018

Reprints

Reprints will not be available from the authors.

Funding

This study is funded by the National Institute for Health Research (NIHR) Doctoral Research Fellowship Grant (DRF-2015-08-023).

Disclaimer

This paper presents independent research funded by the National Institute for Health Research (NIHR). The views expressed are those of the author(s) and not necessarily those of the NHS, the NIHR or the Department of Health.

Running Title

Reporting of Outcomes in Gastric Cancer Surgery Trials.

Word Count: 3,426

Number of tables: 5

Number of figures: 2

Number of appendices: 2

For peer review only

For peer review only - http://bmjopen.bmj.com/site/about/guidelines.xhtml

Structured Abstract

Background

The development of clinical guidelines for the surgical management of gastric cancer should be based on robust evidence from well-designed trials. Being able to reliably compare and combine the outcomes of these trials is a key factor in this process.

Objectives

To examine variation in outcome reporting by surgical trials for gastric cancer; to identify outcomes for prioritisation in an international consensus study to develop a core outcome set in this field.

Data Sources

Systematic literature searches (Evidence Based Medicine, MEDLINE, EMBASE, CINAHL, ClinicalTrials.gov and WHO ICTRP) and a review of study protocols of randomized controlled trials, published between 1996 and 2016.

Intervention

Therapeutic surgical interventions for gastric cancer. Outcomes were listed verbatim, categorized into groups (outcome themes) and examined for definitions and measurement instruments

Results

Of 1919 abstracts screened, 32 trials (9,073 participants) were identified. A total of 749 outcomes were reported of which 96 (13 per cent) were accompanied by an attempted definition. No single outcome was reported by all trials. 'Adverse events' was the most frequently reported 'outcome theme' in which 240 unique terms were described. 12 trials (38%) classified complications according to severity, with 5 (16%) using a formal classification system (Clavien-Dindo or Accordion scale). Of 27 trials which described 'short-term' mortality, 15 (47%) used one of 5 different definitions. Six out of the 32 trials (19%) described 'patient-reported outcomes'.

Conclusion

For peer review only - http://bmjopen.bmj.com/site/about/guidelines.xhtml

Reporting of outcomes in gastric cancer surgery trials is inconsistent. A consensus approach to develop a minimum set of well-defined, standardized outcomes to be used by all future trials examining therapeutic surgical interventions for gastric cancer is needed. This should consider the views of all key stakeholders, including patients.

KEYWORDS:

Surgical Oncology

Stomach Neoplasms

Outcome Assessment

Delphi Technique

Patient Reported Outcome Measures

Treatment Outcome

Outcome reporting

For peer review only

Article Summary

Strengths and Limitations of This Study

- This systematic review is the first to describe the variation in outcome reporting within the field of surgical trials for gastric cancer.
- The study is based on a reproducible and transparent methodology which has been subjected to critical appraisal during a peer-review process. The study forms part of a larger project (The GASTROS Study) to develop a 'core outcome set' (COS) for use in surgical trials for gastric cancer and was reviewed and funded by the National Institute of Health Research (UK).
- Only English-language and randomized studies were included in the analysis. Expanding the search may have resulted in the identification of other relevant outcomes reported in this field.

For peer review only

Main Article

Introduction

Background

Gastric cancer remains a leading cause of cancer-related death globally[1]. Long-term survival remains poor and has not improved significantly over the last four decades[2]. Whilst there has been a shift to multi-modal therapy over the last decade, surgery remains the primary method of curative treatment. Many developments in surgical techniques aim to improve long-term survival, whilst minimizing post-operative complications. Understanding which of these approaches are optimal for patients should be based on robust evidence from well-designed trials. This process involves the synthesis of evidence in the form of systematic reviews which can only be reliably undertaken if trials report the same outcomes and measure them in the same manner.

This review forms part of the first stage of a three-stage study, which intends to examine and address problems with inconsistent outcome reporting in gastric cancer surgery trials (GASTROS – **GA**stric Cancer **S**urgery **TR**ials **R**eported **O**utcome **S**tandardisation). The study aims to develop a 'core outcome set' (COS) – a minimum group of standardized and well-defined outcomes, relevant to key stakeholders and measured by all trials[3] – to standardize the reporting of outcomes in randomized control trials within this field. Our previously published study protocol contains an overview of all three stages[4].

Within our study protocol, we described the results from a 'rapid review' of gastric cancer surgery trials during a 24-month period which demonstrated significant variations in outcome reporting. We hypothesised that these variations were likely to represent a more widespread problem within this field. Inconsistencies in outcome reporting are prevalent within the medical literature and contribute significantly to 'research waste'[5]. Several reviews have demonstrated that trials within the same field often report different outcomes, define them poorly and use various outcome measurement instruments[6–9]. This results in data which cannot be reliably compared or combined leading to further confusion within the evidence base. As such, initiatives such as COMET (Core Outcome Measures in Effectiveness Trials) were formed to promote the development of COS to address these issues[3].

For peer review only - http://bmjopen.bmj.com/site/about/guidelines.xhtml

With respect to surgical trials for gastric cancer, a) no rigorous examination of outcome reporting has been previously undertaken and b) there is no COS for use in this field.

Aims & Objectives

This review aims demonstrate whether further work to develop a COS to be used in surgical trials for gastric cancer is required. Specifically, the objectives are:

1. to examine the degree of variation in the reporting of outcomes described by gastric cancer surgery trials.
2. to generate a 'long-list' of potentially important outcomes which will be prioritized during a Delphi survey in stage two of the study.

For peer review only

Methods

Definitions

The GASTROS study, and more specifically this review, focuses on outcome reporting in 'therapeutic surgical trials'. A 'surgical trial' has been previously defined as one of the following [10]:

- Type 1 - A trial of medical interventions in surgical patients
- Type 2 - A trial which compares a surgical intervention to another surgical intervention
- Type 3 - A trial which compares a surgical intervention to a non-surgical intervention

The GASTROS study focuses on 'type 2' trials due to the significant research activity within this field (a detailed justification can be found in our study protocol)[4]. In the context of gastric cancer, a 'therapeutic surgical intervention' is defined as a potentially curative procedure which aims to excise the gastric neoplasm resulting in partial or total organ loss.

Search strategy

A summary of the review's inclusion and exclusion criteria is summarised in table 1, with details of our search strategy presented below. An example search algorithm for the Medline via OVID database is presented in appendix 1.

Timeline

Trials were searched from 1996 when the first CONSORT statement for the reporting of RCTs was published up to and including March 2016. The COS aims to influence trial design regardless of the country of origin of participating centers and patients. Many of the surgical interventions (e.g. D2 lymphadenectomy) which have recently been examined in the West have long been established practice in the Far East and Asia. Searching trials over a 20-year period allows a comprehensive understanding of which outcomes have been measured for similar trials regardless of the trial location.

Identifying studies

Detailed search strategies were developed for each of the following electronic databases examined:

- Evidence Based Medicine Reviews via OVID

For peer review only - http://bmjopen.bmj.com/site/about/guidelines.xhtml

- Cochrane Database of Systematic Reviews 2005 to March 30, 2016,
- ACP Journal Club 1991 to March 2016,
- Database of Abstracts of Reviews of Effects 1st Quarter 2016
- Cochrane Central Register of Controlled Trials February 2016
- Health Technology Assessment 1st Quarter 2016
- NHS Economic Evaluation Database 1st Quarter 2016

- MEDLINE via OVID (January 1st 1996 to March 30, 2016)
- EMBASE via OVID (January 1st 1996 to March 30, 2016);
- CINAHL via EBSCO (January 1st 1996 to March 30, 2016).

In order to identify surgical interventions and outcome measures being used in current studies, we searched the following databases for protocols of ongoing trials, including completed trials not yet published:

- The US National Institutes of Health Trials Register (http://clinicaltrials.gov);
- The WHO International Clinical Trials Registry Platform (http://apps.who.int/trialsearch/default.aspx).

Non-English language studies were excluded from this review due to resource limitations. Trials published only as conference abstracts were excluded as they are often limited by 'word count' and hence the abstract would not represent a comprehensive list of outcomes measured in the respective study.

Assessment of eligibility

For quality assurance, two review authors (BA and AMG) independently screened the titles and abstracts retrieved from the electronic searches. This assessment was undertaken in groups of ten

For peer review only - http://bmjopen.bmj.com/site/about/guidelines.xhtml

abstracts in reverse chronological order. Once there was complete agreement with two consecutive groups of ten abstracts, the remaining abstracts were split and each reviewer screened independently. Full text copies of all study publications that appeared to meet the inclusion criteria were obtained. Full text copies were also obtained where there was insufficient information in the title or abstract to make a clear judgement. Systematic reviews of RCTs were also retrieved to find studies which had previously not been identified.

BA and AMG independently assessed the full text copies for eligibility. This assessment was undertaken in groups of ten publications in reverse chronological order. Once there was complete agreement with two consecutive groups of ten abstracts, the remaining publications were split, and each reviewer extracted data independently. Any disagreements were resolved through discussion. There were no unresolved disagreements that required referral to the GASTROS study management team for a final decision.

Data Extraction

BA and AMG independently reviewed all eligible publications and extracted data (described below) into a Microsoft Excel (Version 2013, Microsoft, Washington, DC, USA) spreadsheet.

Publication versus Study

It is not uncommon that investigators publish results at different stages of their trial and with each publication present a new set of outcomes. The GASTROS study team decided to amalgamate the outcomes published in all publications associated with a single trial to more fairly reflect outcomes being reported by research groups.

Trial Characteristics

The following data were recorded for each trial:

1. Author details
2. Title of publication

For peer review only

3. Journal

4. Year of publication

5. Number of participating centres

6. Country of 1st Author

7. Countries of participating centres

8. Total number of patients recruited to the study

9. Length of follow up

10. Interventions being investigated

Outcomes

We defined an outcome as 'a unique endpoint which attempts to describe health-related changes that occur secondary to a therapeutic intervention'[4]. The following data were recorded for each outcome:

1. Outcome measured (and whether stated as primary or secondary outcome). Where a primary outcome was not explicitly stated, the outcome on which the sample size calculation was based was taken as the primary outcome.

2. Whether the outcome was defined or not. Outcomes were considered defined if text of their meaning or a citation was provided.

3. The definition of the outcome.

4. The method of outcome measurement (indicators and/or tools used, if relevant).

5. Time points and time-period at or during which the outcome was measured (for example quality of life at 3-months post-surgery).

For peer review only - http://bmjopen.bmj.com/site/about/guidelines.xhtml

Merging Outcomes & Grouping Under 'Themes'

Outcomes were extracted verbatim from publications and minimal merging of terms was undertaken. Outcomes were merged to accommodate for variant spellings of the same words. For example, 'anastomotic leak', 'anastomotic leak**age**' and 'anastomotic leak**s**' were merged into 'anastomotic leak'. The verbatim texts and merged terms were verified and authorized respectively by the study management group.

From the experience of other groups undertaking reviews of outcome reporting, the resulting lists of outcomes are generally extremely long and unwieldy[6]. Consequently, developing a method to organize these outcomes has been necessary. The subject of taxonomy in outcome reporting, including hierarchical structure and which terms/definitions to use, is an emerging area of great significance. We set out our definitions a priori, which can be found in our study protocol[11]. Many COS developers have organized their outcomes into broad categories with common 'themes'. Our study is one of only a handful addressing outcome reporting in surgical trials related to the gastro-intestinal (GI) tract. At the time of data analysis, we opted to group outcomes under 'themes' (detailed in table 2) similar to those described by other surgical COS[6–8,12]. Doing enables COS researchers to more readily understand trends in outcome reporting within the field of GI surgery. Whilst the themes used in our review enable the reader to understand the types of outcomes being reported, this system has not been developed through wider consensus and has not been subject to a validation process.

At the time of writing, a broader taxonomy for outcome classification had been proposed[13]. This system aims to address some of the ambiguity associated with outcome classification on a wider-scale and organizes outcomes under 38 'outcomes domains' which sit under 5 'outcomes areas' ('mortality/survival', 'physiological/clinical', 'life impact', 'resource use' and 'adverse events'). Whilst the authors have demonstrated that this system is comprehensive and applicable to trials irrespective of the field being studied, they have called for further validation of their work.

For peer review only - http://bmjopen.bmj.com/site/about/guidelines.xhtml

Patient & Public Involvement

A Study Advisory Group (SAG) forms part of the management structure of the wider GASTROS study[4], of which this review forms part of the first stage. The SAG is made up of key stakeholder representatives including patients, oncology nurses and surgeons. The group provides advice on the methodology of the study, general delivery of the study against its stated objectives, and ensures that the viewpoints of all stakeholder groups are considered. The results of this systematic review were presented to a SAG meeting; the ensuing discussion influenced certain aspects of the results section within this paper such as the emphasis on patient-reported outcomes.

For peer review only

For peer review only - http://bmjopen.bmj.com/site/about/guidelines.xhtml

Table 1. Inclusion and exclusion criteria for this review.

	Included	Excluded
Types of Studies	• Type 2* surgical randomized controlled trials (RCTs) and protocols of surgical RCTs (all trial phases).<br>• Systematic reviews of type 2 surgical RCTs.<br>• English Language studies.	• Type 1 or type 3* surgical RCTs and systematic reviews of type 1 or type 3 RCTs.<br>• Non-randomized studies.<br>• Non-English language studies.
Population	• Patients aged 18 years and over.	• Patients below the age of 18.
Interventions	• Partial or total gastrectomy.<br>• Surgery with curative intent.	• Esophagectomy for gastro-esophageal junctional tumors.<br>• Surgery with non-curative intent (i.e. in stage 4 cancer with prior expectation of an R1 or R2 resection) for the relief of symptoms such as gastric outlet obstruction or bleeding.<br>• Endoscopic interventions.
Conditions	• Invasive cancer of the stomach and gastro-esophageal junction.	• Dysplasia or non-invasive gastric neoplasms.<br>• Sarcoma (including gastrointestinal stromal tumors).<br>• Gastric lymphoma.

For peer review only - http://bmjopen.bmj.com/site/about/guidelines.xhtml

*Type 1 - a trial of medical interventions in surgical patients, type 2 - a trial which compares a surgical intervention to another surgical intervention, type 3 - a trial which compares a surgical intervention to a non-surgical intervention[10].

For peer review only

For peer review only - http://bmjopen.bmj.com/site/about/guidelines.xhtml

Table 2. Number of times at least one outcome from respective theme was reported in published trials and unpublished or actively recruiting trial protocols.

**Outcome Theme**	**Theme Definition**	Published Trials (n=32) (%)	Unpublished or Actively Recruiting Trial Protocols (n=23**) (%)
Cost	Relating to delivery of surgery as part of clinical care within a healthcare system.	1 (3)	5 (23)
Patient Pathway	Outcomes related to the flow of patients through the healthcare system (e.g. hospital stay, readmission).	20 (63)	4 (17)
Patient-Reported Outcomes	Outcomes taken from the patient perspective.*	6 (19)	13 (57)
Surviving & Controlling Cancer	Measures of disease recurrence or disease progression.	15 (47)	17 (74)
Mortality	Outcomes related to short and long-term survival/death rates and cause of death.	27 (84)	19 (83)
	Short-term mortality/peri-operative death.	27 (84)	10 (43)
	Long-term survival.	13 (41)	19 (44)
Technical Aspects of Surgery	Outcomes recorded directly in the operating theatre (e.g. operation time, blood loss).	31 (97)	13 (57)

For peer review only - http://bmjopen.bmj.com/site/about/guidelines.xhtml

Recovery from Surgery	Report of patient condition following surgery and the ability to return to preoperative or premorbid state.	16 (50)	9 (39)
Adverse Events	Forms of short- and long-term postoperative complications following surgery.	31 (97)	18 (78)

* Certain patient-reported outcomes may fall under other 'themes', e.g. 'post-operative pain' may relate to 'recovery from surgery'. ** One trial protocol contained no information about planned outcomes to report, therefore 23 out of total 24 trials were included in this table.

For peer review only - http://bmjopen.bmj.com/site/about/guidelines.xhtml

Results

Summary

A total of 1,919 abstracts were screened which resulted in the identification of 48 publications from 32 trials (having recruited a total of 9073 patients) eligible for inclusion in the review (figure 1). A further 875 protocols were screened which identified 24 active or unpublished trials aiming to recruit 10,761 patients. A summary of all trials included in the analysis is described in table 3. During the data extraction process, no disagreements requiring discussion in the study management group arose between the two independent reviewers. A total of 749 (392 unique terms) outcomes were reported of which 13% (96 out of 749) were accompanied by an attempted definition. Thirty-eight percent of trials (12 out of 32) described a primary outcome or provided a sample size calculation. No single outcome was reported in every trial.

Analysis of Outcomes According to Themes

Outcomes were organised into eight 'outcome themes', illustrated in figure 2 and described in table 2. A comprehensive list of reported outcomes is presented in appendix 2.

Mortality

Death after surgery was generally described as 'short-term' and 'long-term' survival. Long-term survival was used as a primary outcome measure in 41% of trials (13 out of 32). The terms used to describe long-term mortality and the time-points at which they were measured was inconsistent (table 4). 'Short-term' mortality was reported by 84% of trials (27 out of 32) of which fifteen provided one of the following definitions (frequency each definition was used is presented in brackets):

1. 'Death within **30-days** of surgery' (3/15)
2. 'Death of **any cause** within **30 days**, or death within the **same hospitalization' (9/15)**
3. **'In-hospital** deaths and 'deaths' **within 1 month' (1/15)**
4. 'Death within **30 days** of the operation or during **any hospital stay' (1/15)**
5. **'Any death** that occurred during the **hospital stay (1/15)**

For peer review only - http://bmjopen.bmj.com/site/about/guidelines.xhtml

Adverse Events

Adverse events were the commonest outcome theme to be reported and made up half of the ten most reported outcomes (table 5). 'Anastomotic leak' was the commonest adverse event to be reported and was described using 5 different definitions (frequency each definition was used is presented in brackets):

- 'Clinical and radiological diagnosis' (2).
- 'Confirmed by gastrointestinal x-ray imaging, endoscopy, or angiography' (1).
- 'Dehiscence confirmed by radiographic examination using contrast medium' (1).
- 'Type I anastomotic leakage: a small localized leakage at the esophagojejunal anastomosis, without pleural or abdominal spillage, demonstrated by radiologic studies with barium' (1).
- 'Type II anastomotic leakage: an important dehiscence of the esophago-jejunal anastomosis, with pleural or abdominal dissemination, appearance of intestinal content through the drains, a positive methylene blue test (appearance of orally ingested methylene blue through the drains), and clear demonstration of this leakage by radiologic contrast studies' (1).

Adverse events were categorized by 12 out of 32 trials using terms including 'major', 'minor', 'short-term' and 'long-term'. Five trials used a formal classification system (Clavien-Dindo Classification or Accordian Severity Grading).

Patient Reported Outcomes

Patient-reported Outcomes (PROs) were reported in 19% of trials (6 out of 32) and included measures of quality of life (QoL) (n=3) and 'pain' (n=3). QoL was measured using validated tools for gastric cancer in two trials (EORTC QLQ-C30 with QLQ and Spitzer QoL Index) and a non-validated tool in one trial. Pain was measured using 3 different visual-analogue scales.

Multi-Centre Trials

Forty per cent (13 out of 32) of studies were multi-centre trials (table 2). Ninety-two percent (12 out of 13) of trials stated a primary outcome measure (5-year overall survival, 5-year survival, all-cause

For peer review only - http://bmjopen.bmj.com/site/about/guidelines.xhtml

mortality and 3-year disease free survival). Sixteen percent (63 out of 393) of all outcomes reported in these trials were accompanied by an attempted definition. PRO's were reported in 23% of trials (3 out of 13) with 8% (1 out of 13) of studies reporting 'quality of life' as an outcome.

Findings from Study Protocols

Most of the 24 ongoing or unpublished trials [14–37] are recruiting in China (n=13), with twenty examining 'extent of lymphadenectomy' or minimally invasive approaches to surgery. A total of 220 uniquely termed outcomes are planned to be reported, thirty-five of which (16%) have an accompanying definition in the respective protocol. The commonest term used to report 'long-term survival' is 'overall survival' (OS) which will be measured by sixteen trials. Seven of these trials plan to measure OS after 5-years of follow-up, three at 3-years of follow-up, and six did not identify time points at which OS would be measured. At the time of our search, one trial protocol contained no information about which outcomes are to be measured.

QoL is due to be measured by ten trials (42 per cent) with five trials proposing to use one or a combination of four different measurement instruments (EORTC QLQ-C30 with QLQ-STO22, SF-36, GIQLI and Euro-Quality of Life-5D). Seven protocols described the timing of the quality of life measurements as follows:

- 'Preoperative, postoperative **3 weeks** and postoperative **12 months'.**
- 'In **pre-therapy <7 days**, **pre-operative <7 days**, and post-operative at **12 months** after surgery'.
- **'90th postoperative day'.**
- **'Regularly** for three years after surgery'.
- **'Preoperatively**, **five days postoperatively**, **three** months, **six** months and **one year** postoperatively'.
- **'Baseline**, **1 week**, **1** month, **6**-month, **1 year**, **3 years'.**
- **'6 weeks**, **12**, **24**, **36**, **48** and **50 months** after surgery'.

For peer review only - http://bmjopen.bmj.com/site/about/guidelines.xhtml

Table 3. Published gastric cancer surgery trials included in the study analysis.

Trial Reference(s)	Trial Number	Author	Year of 1st Publication	Participating Countries	Number of Recruiting Sites	Patients Recruited	Interventions
38–41	1	Hartgrink et al, Bonenkamp et al., Sasako et al., Songun et al.	1995	Netherlands	80	1078	• D1 lymphadenectomy<br>• D2 lymphadenectomy
42,43	2	Cuschieri et al.	1996	UK	32 surgeons*	737	• D1 lymphadenectomy<br>• D2 lymphadenectomy
44,45	3	Marubini et al. Bozzetti et al.	1999	Italy	31	615	• D2 subtotal gastrectomy<br>• D2 total gastrectomy
46	4	Maeta et al	1999	Japan	1	70	• D3 total gastrectomy<br>• D4 total gastrectomy
47	5	Furukawa et al.	2000	Japan	1	110	• Total gastrectomy and distal pancreatectomy

For peer review only - http://bmjopen.bmj.com/site/about/guidelines.xhtml

							• Pancreas preserving total gastrectomy
[48]	6	Csendes et al.	2002	Chile	1	187	• D2 total gastrectomy with splenectomy<br>• Spleen preserving D2 total gastrectomy
[49]	7	Kitano et al.	2002	Japan	1	28	• Laparoscopic assisted distal gastrectomy<br>• Open distal gastrectomy
[50]	8	Fujii et al	2003	Japan	1	20	• Laparoscopic assisted distal gastrectomy<br>• Open distal gastrectomy
[51–53]	9	Degiuili et al	2004	Italy	5	267	• D1 lymphadenectomy<br>• D2 lymphadenectomy
[54–57]	10	Sano et al.,<br>Sasako et al.,<br>Kodera et al,<br>Tsujinaka et al.	2004	Japan	24	523	• D2 lymphadenectomy<br>• D2 lymphadenectomy & para-aortic node dissection

For peer review only

For peer review only - http://bmjopen.bmj.com/site/about/guidelines.xhtml

58	11	Shibata et al	2004	Japan	9	81	• Pylorus-preserving gastrectomy<br>• Distal gastrectomy
59	12	Inaba et el.	2004	Japan	1	410	• Midline laparotomy<br>• Transverse laparotomy
60	13	Lee et al.	2005	South Korea	1	47	• Laparoscopic assisted D2 distal gastrectomy<br>• Open D2 distal gastrectomy
61	14	Hayashi et al	2005	Japan	1	28	• Laparoscopic assisted distal gastrectomy with extra-perigastric node dissection.<br>• Open distal gastrectomy with extra-perigastric node dissection.
62	15	Huscher et al	2005	Italy	1	59	• Laparoscopic sub-total gastrectomy<br>• Open sub-total gastrectomy
63–66	16	Wu et al.	2006	Taiwan	1	221	• D1 lymphadenectomy<br>• D3 lymphadenectomy
67,68	17	Sasako et al.	2006	Japan	27	167	• Left Thoracoabdominal approach

For peer review only - http://bmjopen.bmj.com/site/about/guidelines.xhtml

		Kurokawa et al.					• Abdominal trans-hiatal approach
69	18	Yu et al	2006	South Korea	1	207	• Total gastrectomy with splenectomy<br>• Spleen preserving total gastrectomy
70	19	Kulig et al.	2007	Poland	6	275	• D2 lymphadenectomy<br>• D2 lymphadenectomy & para-aortic node dissection
71,72	20	Kim et al.	2008	South Korea	1	164	• Laparoscopic assisted distal gastrectomy<br>• Open distal gastrectomy
73	21	Yonemura et al.	2008	Japan, Taiwan, South Korea	10	269	• D2 lymphadenectomy<br>• D4 lymphadenectomy
74,75	22	Kim et al.	2010	South Korea	13	1416	• Laparoscopic assisted D2 distal gastrectomy<br>• Open distal D2 gastrectomy
76	23	Imamura et al	2011	Japan	11	210	• D2 gastrectomy and bursectomy

For peer review only - http://bmjopen.bmj.com/site/about/guidelines.xhtml

							• D2 gastrectomy without bursectomy
77	24	Cai et al.	2012	China	1	123	• Laparoscopic assisted distal gastrectomy<br>• Open distal gastrectomy
78	25	Chen Hu et al.	2012	China	1	88	• Laparosopic gastrectomy<br>• Open gastrectomy<br>• Standard post-operative protocol<br>• Fast-track post-operative protocol
79	26	Takiguchi	2013	Japan	1	40	• Laparoscopic assisted distal gastrectomy<br>• Open distal gastrectomy
80	27	Lee et al.	2013	South Korea	1	204	• D2 distal gastrectomy<br>• D2 total gastrectomy
81	28	Sakuramoto et al	2013	Japan	1	64	• Laparoscopic assisted distal gastrectomy<br>• Open distal gastrectomy
82	29	Aoyama et al	2014	Japan	1	26	• Laparoscopic assisted distal

For peer review only

							gastrectomy<br>• Open distal gastrectomy
83	30	Hirao et al.	2015	Japan	11	210	• D2 gastrectomy with bursectomy<br>• D2 gastrectomy without bursectomy
84	31	Galizia et al	2015	Italy	1	73	• D1'+' total gastrectomy<br>• D2 total gastrectomy
85	32	Hu et al.	2016	China	14	1056	• Laparoscopic assisted D2 distal gastrectomy<br>• Open D2 distal gastrectomy

*Number of sites not specified.

For peer review only
For peer review only - http://bmjopen.bmj.com/site/about/guidelines.xhtml

Table 4. Reporting of 'long-term mortality' in gastric cancer surgery trials.

Term used	Number of trials reporting outcome	Follow-up period used	Frequency defined	Definitions provided
'Overall Survival' including 'death from all causes'	19	Not described<br>3 years<br>5 years<br>6 years<br>7 years<br>10 years	7	• Date of randomisation until the day of death or the day of last follow-up (censored).<br>• Date of surgery to the date of death from any cause, censoring the follow-up time at the most recent date for living patients.<br>• Date of randomization to the date of death.<br>• Date of randomization to the date of death from any cause.<br>• OS included operative deaths.<br>• OS excluded post-operative deaths.
'Survival' including 'survival period'	13	Not described<br>5 years<br>11 years	4	• Survival excluding operative mortality<br>• Survival 5 years after curative surgery
'Disease-specific survival'	4	Not described	1	• Proportion of patients who had not died from gastric cancer.

For peer review only - http://bmjopen.bmj.com/site/about/guidelines.xhtml

including 'gastric cancer related deaths'		5 years		
Disease-free survival*	1	Not described	1	• Time from randomization to recurrence or death due to any cause.
Recurrence-free survival*	2	Not described	2	• Time from randomization to either the first recurrence or death from any cause.<br>• Time from randomization to the first documentation of cancer recurrence or death from any cause.

*Although these terms do not relate to mortality, they have been included in this table as the definitions provided by papers describe death as an end-point.

For peer review only - http://bmjopen.bmj.com/site/about/guidelines.xhtml

Table 5. The ten most frequently reported outcomes.

Outcome	Theme	Number of trials reporting outcome	Trials reporting the outcome*
Number of lymph nodes dissected/resected/retrieved	Technical aspects of surgery	22	2, 3, 5, 7, 9, 10, 13, 14, 15, 16, 17, 18, 19, 20, 21, 23, 24, 25, 26, 29, 31, 32
Operative time	Technical aspects of surgery	18	4, 5, 7, 8, 10, 11, 13, 14, 15, 16, 20, 22, 23, 24, 25, 26, 27, 30
Anastomotic leak	Adverse events	17	1, 2, 4, 6, 10, 14, 16, 17, 19, 22, 23, 24, 25, 27, 28, 31, 32
Pancreatic fistula	Adverse events	15	1, 2, 4, 5, 10, 16, 17, 19, 22, 23, 26, 27, 28, 29, 32
Duration of hospital stay	Patient pathway	12	1, 2, 5, 6, 7, 9, 13, 15, 20, 23, 26, 28
Duration of post-operative hospital stay	Recovery from surgery	11	2, 7, 9, 11, 14, 16, 19, 24, 25, 27, 32
Pneumonia	Adverse events	11	7, 10, 11, 12, 17, 23, 27, 28, 29, 31, 32
'5-year' survival	Mortality	11	1, 2, 6, 9, 10, 16, 17, 18, 19, 22, 26
Wound infection	Adverse events	10	2, 6, 12, 15, 16, 18, 19, 22, 24, 28
Abdominal abscess	Adverse events	10	5, 9, 10, 16, 17, 19, 23, 26, 28, 29

*See table 1 for trial numbers and associated publications.

For peer review only - http://bmjopen.bmj.com/site/about/guidelines.xhtml

Discussion

This review is the first to examine the subject of outcome reporting in this field and demonstrates significant inconsistencies in the outcomes measured in trials examining therapeutic surgical interventions for gastric cancer. Not only is there disagreement about 'what' outcomes should be measured, but also 'when' and 'how' they should be measured. Consequently, undertaking meta-analyses and systematic reviews of these interventions becomes problematic and impacts negatively on the ability of researchers and clinicians to formulate robust clinical guidelines for the radical treatment of gastric cancer.

This problem is not confined to previously reported trials. Our analysis of outcome reporting in multi-centre studies and review of active trial protocols has demonstrated similar issues and further highlights the potential 'research waste' within this field. Glasziou and Chambers' estimate that 85 per cent of all biomedical research is 'wasted', and that a significant proportion of this can be attributed to problems choosing and reporting relevant outcomes in trials[5]. Thus, if we combine the number of patients who have participated in gastric cancer surgery trials over the last two decades with those that actively recruiting trials wish to attract, a total of twenty thousand patients may have participated in trials which, from a methodological perspective, could have had a far greater benefit and impact. Not only does this represent an inefficient use of time and scarce financial resource, but it may also have a longer-lasting negative impact on future trial participation by patients. Similar issues related to outcome reporting have been identified by several other groups supporting the theory that this is a widespread problem[6–8,12].

Furthermore, if the methodology of a particular trial is not sufficiently robust or the outcomes reported are not relevant to key stakeholders, the natural course will be for other researchers to examine the same interventions again, using a different approach. If these subsequent trials do not address the underlying methodological issues, they only contribute to a perpetual cycle which serves to weaken the evidence base. This is reflected within the field of gastric cancer surgery where thirteen trials have examined minimally invasive gastrectomy and a further 13 are actively recruiting to trials examining the same intervention.

For peer review only - http://bmjopen.bmj.com/site/about/guidelines.xhtml

Reported outcomes should be relevant to key stakeholders including patients. Given the sheer volume of complications that are reported by gastric surgical trials, one may expect to find the impact on QoL is routinely reported. Indeed, QoL has been demonstrated as an important outcome to measure in other gastrointestinal cancer fields[86]. This has certainly not been the case with gastric cancer surgery trials over the last two decades and whilst there seems to be a greater acceptance by trials currently in recruitment that QoL is important to measure (although this group still represents less than half of ongoing trials), there remains great variation in relation to 'how' and 'when' it is measured.

To address these inconsistencies, we believe that a 'core outcome set' (COS) is required for gastric cancer surgery studies. Developing a minimum reporting standard will contribute to maximising the benefits from randomized control trials which are expensive, labour intensive and logistically challenging to set up. A COS does not aim to restrict the outcomes that are reported, but merely to ensure that the most critical outcomes (as decided by key stakeholders) are clearly defined and measured uniformly.

The challenges associated with inconsistent outcome reporting in trials is certainly not confined to the field of gastric cancer. The COMET (Core Outcome Measurement in Effectiveness Trials) initiative database (http://www.comet-initiative.org/studies/search) contains details of over 400 completed, active or planned COS projects from across many different specialities[87]. Whilst experience within this relatively new research field has grown considerably over the last decade, there is still much work to be done to further develop the various methodological approaches which can be applied. The GASTROS study aims to add to this in several ways including examining the role of 'internationalising' COS development by undertaking a multi-language Delphi survey as part of a consensus-seeking process.

Strengths and Limitations

In addition to being the first systematic review to examine this subject, this study is based on a reproducible and transparent methodology which has been subjected to critical appraisal from a study management team and peer-review process; a protocol of the GASTROS study which aims to develop this COS has been published previously[4]. Nonetheless, there are limitations. Including non-English

For peer review only - http://bmjopen.bmj.com/site/about/guidelines.xhtml

and non-randomized studies in our search strategy may have identified other different outcomes reported in this field. However, when finalising our inclusion criteria for this review, the two primary objectives of this review were considered - namely a) to describe the current landscape of outcome reporting in gastric cancer surgery RCTs and b) to take forward a 'long-list' of outcomes to be prioritised (by means of a Delphi survey) to form the basis of a COS for RCTs. Whilst we accept that such a COS would have benefits to non-RCTs and national audits, our primary focus was to improve the quality of RCTs and hence excluding other study types. In addition, there will be an opportunity during the Delphi survey (stage 2 of the GASTROS study) for participants to add further outcomes (not already identified from this review) which key stakeholders deem important to be considered for prioritisation. A further limitation to this review was that it was not prospectively registered on a public database. However, as we describe above, the GASTROS study, including its scope and systematic review plan, has been peer-reviewed and published previously[4].

In summary, the reporting of outcomes in gastric cancer surgery trials is inconsistent and there is large variation with respect to definitions, measurement tools and timing of measurement. This means that data cannot be synthesized efficiently. We believe that a COS to define a minimum set of standards to implement across all gastric surgical trial is warranted.

For peer review only - http://bmjopen.bmj.com/site/about/guidelines.xhtml

Declarations

Ethics Approval and Consent to Participate

Not applicable.

Consent for Publication

Not applicable.

Data Sharing Statement

Upon the completion of the study, the original data file (consisting of extracted data from trial papers) is available upon request from the corresponding author, following discussion and agreements by the study management team.

Competing interests

The authors declare that they have no competing interests.

Funding

This study is funded by the National Institute for Health Research (NIHR) Doctoral Research Fellowship Grant programme (DRF-2015-08-023). The funding body has no role in the study design and implementation, writing the report, or decision to submit the report for publication.

Authors' contributions

BA contributed to the study design and drafted the manuscript. BA and AMG extracted the data from manuscripts. AMG, JMB, PRW and IAB contributed to the study design and revised the manuscript. All authors read and approved the final manuscript.

Authors' Information

Not applicable.

Acknowledgements

The authors would like to thank the patient representatives on the GASTROS Study Advisory Group for their continued advice and invaluable contribution to this study.

For peer review only - http://bmjopen.bmj.com/site/about/guidelines.xhtml

For peer review only

For peer review only - http://bmjopen.bmj.com/site/about/guidelines.xhtml

References

1. Fact Sheets by Cancer. http://globocan.iarc.fr/Pages/fact_sheets_cancer.aspx. Accessed July 24, 2017.

2. Quaresma M, Coleman MP, Rachet B. 40-year trends in an index of survival for all cancers combined and survival adjusted for age and sex for each cancer in England and Wales, 1971–2011: a population-based study. Lancet. 2015;385(9974):1206-1218. doi:10.1016/S0140-6736(14)61396-9.

3. Williamson PR, Altman DG, Bagley H, et al. The COMET Handbook: version 1.0. Trials. 2017;18(S3):280. doi:10.1186/s13063-017-1978-4.

4. Alkhaffaf B, Glenny A-M, Blazeby JM, Williamson P, Bruce IA. Standardising the Reporting of Outcomes in Gastric Cancer Surgery Trials: Protocol for the Development of a Core Outcome Set and Accompanying Outcome Measurement Instrument Set (The GASTROS Study). Trials. 2017;18(1):370. doi:10.1186/s13063-017-2100-7.

5. Chalmers I, Glasziou P. Avoidable waste in the production and reporting of research evidence. Lancet (London, England). 2009;374(9683):86-89. doi:10.1016/S0140-6736(09)60329-9.

6. Hopkins JC, Howes N, Chalmers K, et al. Outcome reporting in bariatric surgery: an in-depth analysis to inform the development of a core outcome set, the BARIACT Study. Obes Rev. 2015;16(1):88-106. doi:10.1111/obr.12240.

7. Blencowe NS, Strong S, McNair AGK, et al. Reporting of short-term clinical outcomes after esophagectomy: a systematic review. Ann Surg. 2012;255(4):658-666. doi:10.1097/SLA.0b013e3182480a6a.

8. Whistance RN, Forsythe RO, McNair AGK, et al. A systematic review of outcome reporting in colorectal cancer surgery. Colorectal Dis. 2013;15(10):e548-60. doi:10.1111/codi.12378.

9. Duffy JMN, Hirsch M, Gale C, et al. A systematic review of primary outcomes and outcome measure reporting in randomized trials evaluating treatments for pre-eclampsia. Int J Gynecol

For peer review only - http://bmjopen.bmj.com/site/about/guidelines.xhtml

Obstet. 2017;139(3):262-267. doi:10.1002/ijgo.12298.

10. McCulloch P. Randomised trials in surgery: problems and possible solutions. BMJ. 2002;324(7351):1448-1451. doi:10.1136/bmj.324.7351.1448.

11. Alkhaffaf B, Glenny A-M, Blazeby JM, Williamson P, Bruce IA. Standardising the reporting of outcomes in gastric cancer surgery trials: Protocol for the development of a core outcome set and accompanying outcome measurement instrument set (the GASTROS study). Trials. 2017;18(1). doi:10.1186/s13063-017-2100-7.

12. Currie A, Brigic A, Blencowe NS, et al. Systematic review of surgical innovation reporting in laparoendoscopic colonic polyp resection. Br J Surg. 2015;102(2):e108-e116. doi:10.1002/bjs.9675.

13. Dodd S, Clarke M, Becker L, Mavergames C, Fish R, Williamson PR. A taxonomy has been developed for outcomes in medical research to help improve knowledge discovery. J Clin Epidemiol. 2018;96:84-92. doi:10.1016/j.jclinepi.2017.12.020.

14. Hasegawa S, Yamamoto Y, Taguri M, et al. A Randomized Phase II Trial of Omentum-preserving Gastrectomy for Advanced Gastric Cancer. doi:10.1093/jjco/hys208.

15. Nakamura K, Katai H, Mizusawa J, et al. A Phase III Study of Laparoscopy-assisted Versus Open Distal Gastrectomy with Nodal Dissection for Clinical Stage IA/IB Gastric Cancer (JCOG0912). Jpn J Clin Oncol. 2013;43(3):324-327. doi:10.1093/jjco/hys220.

16. Yoshikawa T, Fukunaga T, Taguri M, et al. Laparoscopic or Open Distal Gastrectomy After Neoadjuvant Chemotherapy for Operable Gastric Cancer, a Randomized Phase II Trial (LANDSCOPE Trial). Jpn J Clin Oncol. 2012;42(7):654-657. doi:10.1093/jjco/hys057.

17. Effect of Laparoscopy-Assisted Distal Gastrectomy for Locally Advanced Gastric Cancer - Full Text View - ClinicalTrials.gov. https://clinicaltrials.gov/ct2/show/NCT02464215. Accessed July 25, 2017.

18. The Long-term Effect of D4 Lymphadenectomy for Gastric Cancer - Full Text View -

For peer review only - http://bmjopen.bmj.com/site/about/guidelines.xhtml

ClinicalTrials.gov. https://clinicaltrials.gov/ct2/show/NCT02423278. Accessed July 25, 2017.

19. A Comparison of Laparoscopic With Open Distal Gastrectomy in Advanced Gastric Cancer After Neoadjuvant Chemotherapy - Full Text View - ClinicalTrials.gov. https://clinicaltrials.gov/ct2/show/NCT02404753. Accessed July 25, 2017.

20. Randomized Controlled Trials Comparing Clinical Outcomes of 3D Versus 2D Laparoscopic Surgery for Gastric Cancer - Full Text View - ClinicalTrials.gov. https://clinicaltrials.gov/ct2/show/NCT02327481. Accessed July 25, 2017.

21. Prospective Study on Modified Delta-shaped Gastroduodenostomy in Totally Laparoscopic Surgery for Distal Gastric Cancer - Full Text View - ClinicalTrials.gov. https://clinicaltrials.gov/ct2/show/NCT02289183. Accessed July 25, 2017.

22. Haverkamp L, Brenkman HJF, Seesing MFJ, et al. Laparoscopic versus open gastrectomy for gastric cancer, a multicenter prospectively randomized controlled trial (LOGICA-trial). BMC Cancer. 2015;15:556. doi:10.1186/s12885-015-1551-z.

23. Laparoscopy-assisted and Open Distal Gastrectomy for Gastric Cancer in the Elderly Patients. https://clinicaltrials.gov/ct2/show/NCT02246153.

24. A Clinical Trial of D1+ Versus D2 Distal Gastrectomy for Stage IB & II Advanced Gastric Cancer - Full Text View - ClinicalTrials.gov. https://clinicaltrials.gov/ct2/show/NCT02144727. Accessed July 25, 2017.

25. Straatman J, van der Wielen N, Cuesta MA, et al. Surgical techniques, open versus minimally invasive gastrectomy after chemotherapy (STOMACH trial): study protocol for a randomized controlled trial. Trials. 2015;16(1):123. doi:10.1186/s13063-015-0638-9.

26. D2 and D2+ Radical Surgery for the Treatment of Advanced Distal Gastric Cancer - Full Text View - ClinicalTrials.gov. https://clinicaltrials.gov/ct2/show/NCT01836991. Accessed July 25, 2017.

27. Park JY, Kim Y-W, Ryu KW, et al. Assessment of laparoscopic stomach preserving surgery

For peer review only

For peer review only - http://bmjopen.bmj.com/site/about/guidelines.xhtml

with sentinel basin dissection versus standard gastrectomy with lymphadenectomy in early gastric cancer–A multicenter randomized phase III clinical trial (SENORITA trial) protocol. BMC Cancer. 2016;16(1):340. doi:10.1186/s12885-016-2336-8.

28. Laparoscopy-assisted Total Gastrectomy for Clinical Stage I Gastric Cancer (KLASS-03). https://clinicaltrials.gov/ct2/show/study/NCT01584336. Accessed July 25, 2017.

29. Efficacy of Laparoscopic Subtotal Gastrectomy With D2 Lymph Node Dissection for Locally Advanced Gastric Cancer (KLASS-02-RCT). https://clinicaltrials.gov/ct2/show/NCT01456598.

30. GCSSG-SPNX: Trial to Evaluate Splenectomy in Total Gastrectomy for Proximal Gastric Carcinoma: JCOG0110 - Full Text View - ClinicalTrials.gov. https://clinicaltrials.gov/ct2/show/NCT00112099. Accessed July 25, 2017.

31. Randomized Controlled Trial to Evaluate Laparoscopic versus Open Surgery for Advanced Gastric Cancer (JLSSG0901: Adv.GC-LAP/OPEN, PII/III). http://apps.who.int/trialsearch/Trial2.aspx?TrialID=JPRN-UMIN000003420. Accessed July 25, 2017.

32. Comparsion of D2 verus D3 surgical procedure outcome in locally advanced gastric cancer patients following perioperative chemotherapy. http://ctri.nic.in/Clinicaltrials/pmaindet2.php?trialid=6992&EncHid=&userName=CTRI/2013/08/003882. Accessed July 25, 2017.

33. Prospective clinical trial on the long-term outcome and survival of vagus (coeliac branch)-preserving gastrectomy for cT1-3 gastric cancer. http://www.chictr.org.cn/showprojen.aspx?proj=4360. Accessed July 25, 2017.

34. Prospective clinical trial on the long-term outcome and survival of pylorus-preserving gastrectomy for early gastric cancer. http://www.chictr.org.cn/showprojen.aspx?proj=4361. Accessed July 25, 2017.

For peer review only - http://bmjopen.bmj.com/site/about/guidelines.xhtml

35. Prospective and open multicenter randomize control study for enlarged lymphadenectomy of gastric cancer. http://www.chictr.org.cn/showprojen.aspx?proj=7870. Accessed July 25, 2017.

36. Open vs minimal invasive surgery for gastric submucosal tumor: a randomized trial on efficacy and safety. http://www.chictr.org.cn/showprojen.aspx?proj=4811. Accessed July 25, 2017.

37. A Multicenter Clinical Trial on Laparoscopic Gastric Cancer Surgery Compared With Open Surgery - Full Text View - ClinicalTrials.gov. https://clinicaltrials.gov/ct2/show/NCT02302794. Accessed July 25, 2017.

38. Hartgrink HH. Extended Lymph Node Dissection for Gastric Cancer: Who May Benefit? Final Results of the Randomized Dutch Gastric Cancer Group Trial. J Clin Oncol. 2004;22(11):2069-2077. doi:10.1200/JCO.2004.08.026.

39. Bonenkamp JJ, Songun I, Welvaart K, et al. Randomised comparison of morbidity after D1 and D2 dissection for gastric cancer in 996 Dutch patients. Lancet. 1995;345(8952):745-748. doi:10.1016/S0140-6736(95)90637-1.

40. Sasako M. Risk factors for surgical treatment in the Dutch Gastric Cancer Trial. Br J Surg. 1997;84(11):1567-1571.

41. Songun I, Putter H, Kranenbarg EM-K, Sasako M, van de Velde CJH. Surgical treatment of gastric cancer: 15-year follow-up results of the randomised nationwide Dutch D1D2 trial. Lancet Oncol. 2010;11(5):439-449. doi:10.1016/S1470-2045(10)70070-X.

42. Cuschieri a, Weeden S, Fielding J, et al. Patient survival after D1 and D2 resections for gastric cancer: long-term results of the MRC randomized surgical trial. Surgical Co-operative Group. Br J Cancer. 1999;79(9-10):1522-1530. doi:10.1016/S0959-8049(01)80998-9.

43. Cuschieri a, Fayers P, Fielding J, et al. Postoperative morbidity and mortality after D1 and D2 resections for gastric cancer: preliminary results of the MRC randomised controlled surgical trial. The Surgical Cooperative Group. Lancet. 1996;347:995-999. doi:10.1016/S0959-

For peer review only - http://bmjopen.bmj.com/site/about/guidelines.xhtml

8049(01)80998-9.

44. Marubini E, Bozzetti F, Miceli R, Bonfanti G, Gennari L. Lymphadenectomy in gastric cancer: prognostic role and therapeutic implications. Eur J Surg Oncol. 2002;28(4):406-412. doi:Clinical Trial Comparative Study Randomized Controlled Trial Research Support, Non-U.S. Gov't.

45. Bozzetti F, Marubini E, Bonfanti G, Miceli R, Piano C, Gennari L. Subtotal versus total gastrectomy for gastric cancer: five-year survival rates in a multicenter randomized Italian trial. Italian Gastrointestinal Tumor Study Group. Ann Surg. 1999;230(2):170-178. doi:10.1097/00000658-199908000-00006.

46. Maeta M, Yamashiro H, Saito H, et al. A prospective pilot study of extended (D3) and superextended para-aortic lymphadenectomy (D4) in patients with T3 or T4 gastric cancer managed by total gastrectomy. Surgery. 1999;125(3):325-331. doi:10.1016/S0039-6060(99)70244-8.

47. Furukawa H, Hiratsuka M, Ishikawa O, et al. Total gastrectomy with dissection of lymph nodes along the splenic artery: a pancreas-preserving method. Ann Surg Oncol. 2000;7(9):669-673.

48. Csendes A, Burdiles P, Rojas J, Braghetto I, Diaz JC, Maluenda F. A prospective randomized study comparing D2 total gastrectomy versus D2 total gastrectomy plus splenectomy in 187 patients with gastric carcinoma. Surgery. 2002;131(4):401-407. doi:10.1067/msy.2002.121891.

49. Kitano S, Shiraishi N, Fujii K, Yasuda K, Inomata M, Adachi Y. A randomized controlled trial comparing open vs laparoscopy-assisted distal gastrectomy for the treatment of early gastric cancer: an interim report. Surgery. 2002;131(1 Suppl):0-11. doi:a120115 [pii].

50. Fujii K, Sonoda K, Izumi K, Shiraishi N, Adachi Y, Kitano S. T lymphocyte subsets and Th1/Th2 balance after laparoscopy-assisted distal gastrectomy. Surg Endosc Other Interv

For peer review only - http://bmjopen.bmj.com/site/about/guidelines.xhtml

Tech. 2003;17(9):1440-1444. doi:10.1007/s00464-002-9149-3.

51. Degiuli M, Sasako M, Calgaro M, et al. Morbidity and mortality after D1 and D2 gastrectomy for cancer: interim analysis of the Italian Gastric Cancer Study Group (IGCSG) randomised surgical trial. Eur J Surg Oncol. 2004;30(3):303-308. doi:10.1016/j.ejso.2003.11.020.

52. Degiuli M, Sasako M, Ponti a. Morbidity and mortality in the italian gastric cancer study group randomized clinical trial of D1 versus D2 resection for gastric cancer. Br J Surg. 2010;97(5):643-649. doi:10.1002/bjs.6936.

53. Degiuli M, Sasako M, Ponti A, et al. Randomized clinical trial comparing survival after D1 or D2 gastrectomy for gastric cancer. Br J Surg. 2014;101(2):23-31. doi:10.1002/bjs.9345.

54. Sasako M, Sano T, Yamamoto S, et al. D2 lymphadenectomy alone or with para-aortic nodal dissection for gastric cancer. N Engl J Med. 2008;359(5):453-462. doi:10.1056/NEJMoa0707035.

55. Takeshi Sano. Gastric Cancer Surgery : Morbidity and Mortality Results From a Prospective Randomized Controlled Trial Comparing D2 and Extended Para-Aortic Lymphadenectomy — Japan Clinical Oncology Group Study 9501. J Clin Oncol. 2004;22(14):2767-2773. doi:10.1200/JCO.2004.10.184.

56. Kodera Y, Sasako M, Yamamoto S, Sano T, Nashimoto a, Kurita a. Identification of risk factors for the development of complications following extended and superextended lymphadenectomies for gastric cancer. Br J Surg. 2005;92(9):1103-1109. doi:10.1002/bjs.4979.

57. Tsujinaka T, Sasako M, Yamamoto S, et al. Influence of overweight on surgical complications for gastric cancer: results from a randomized control trial comparing D2 and extended para-aortic D3 lymphadenectomy (JCOG9501). Ann Surg Oncol. 2007;14(2):355-361. doi:10.1245/s10434-006-9209-3.

58. Shibata C, Shiiba K, Funayama Y, et al. Outcomes after Pylorus-preserving Gastrectomy for

For peer review only - http://bmjopen.bmj.com/site/about/guidelines.xhtml

Early Gastric Cancer: A Prospective Multicenter Trial. World J Surg. 2004;28(9):857-861. doi:10.1007/s00268-004-7369-5.

59. Inaba T, Okinaga K, Fukushima R, et al. Prospective randomized study of two laparotomy incisions for gastrectomy: Midline incision versus transverse incision. Gastric Cancer. 2004;7(3):167-171. doi:10.1007/s10120-004-0291-6.

60. Lee JH, Han HS. A prospective randomized study comparing open vs laparoscopy-assisted distal gastrectomy in early gastric cancer: early results. Surg Endosc. 2005;19(2):168-173. doi:10.1007/s00464-004-8808-y.

61. Hayashi H, Ochiai T, Shimada H, Gunji Y. Prospective randomized study of open versus laparoscopy-assisted distal gastrectomy with extraperigastric lymph node dissection for early gastric cancer. Surg Endosc. 2005;19(9):1172-1176. doi:10.1007/s00464-004-8207-4.

62. Huscher CGSS, Mingoli A, Sgarzini G, et al. Laparoscopic versus open subtotal gastrectomy for distal gastric cancer: five-year results of a randomized prospective trial. Ann Surg. 2005;241(2):232-237. doi:10.1097/01.sla.0000151892.35922.f2.

63. Wu C-W, Hsiung C a, Lo S-S, et al. Nodal dissection for patients with gastric cancer: a randomised controlled trial. Lancet Oncol. 2006;7(4):309-315. doi:10.1016/S1470-2045(06)70623-4.

64. Wu C-W, Chiou J-M, Ko F-S, et al. Quality of life after curative gastrectomy for gastric cancer in a randomised controlled trial. Br J Cancer. 2008;98(1):54-59. doi:10.1038/sj.bjc.6604097.

65. Wu CW, Hsiung C a., Lo SS, Hsieh MC, Shia LT, Whang-Peng J. Randomized clinical trial of morbidity after D1 and D3 surgery for gastric cancer. Br J Surg. 2004;91(3):283-287. doi:10.1002/bjs.4433.

66. Wu C-W, Chang I-S, Lo S, et al. Complications following D3 gastrectomy: post hoc analysis of a randomized trial. World J Surg. 2006;30(1):12-16. doi:10.1007/s00268-005-7951-5.

67. Sasako M, Sano T, Yamamoto S, et al. Left thoracoabdominal approach versus abdominal-

For peer review only - http://bmjopen.bmj.com/site/about/guidelines.xhtml

transhiatal approach for gastric cancer of the cardia or subcardia: a randomised controlled trial. Lancet Oncol. 2006;7(8):644-651. doi:10.1016/S1470-2045(06)70766-5.

68. Kurokawa Y, Sasako M, Sano T, et al. Ten-year follow-up results of a randomized clinical trial comparing left thoracoabdominal and abdominal transhiatal approaches to total gastrectomy for adenocarcinoma of the oesophagogastric junction or gastric cardia. Br J Surg. 2015;102(4):341-348. doi:10.1002/bjs.9764.

69. Yu W, Choi GS, Chung HY. Randomized clinical trial of splenectomy versus splenic preservation in patients with proximal gastric cancer. Br J Surg. 2006;93(5):559-563. doi:10.1002/bjs.5353.

70. Kulig J, Popiela T, Kolodziejczyk P, Sierzega M, Szczepanik A. Standard D2 versus extended D2 (D2+) lymphadenectomy for gastric cancer: an interim safety analysis of a multicenter, randomized, clinical trial. Am J Surg. 2007;193(1):10-15. doi:10.1016/j.amjsurg.2006.04.018.

71. Kim YW, Baik YH, Yun YH, et al. Improved quality of life outcomes after laparoscopy-assisted distal gastrectomy for early gastric cancer: results of a prospective randomized clinical trial. Ann Surg. 2008;248(5):721-727. doi:10.1097/SLA.0b013e318185e62e\r00000658-200811000-00006 [pii].

72. Kim YW, Yoon HM, Yun YH, et al. Long-term outcomes of laparoscopy-assisted distal gastrectomy for early gastric cancer: Result of a randomized controlled trial (COACT 0301). Surg Endosc Other Interv Tech. 2013;27(11):4267-4276. doi:10.1007/s00464-013-3037-x.

73. Yonemura Y, Wu CC, Fukushima N, et al. Randomized clinical trial of D2 and extended paraaortic lymphadenectomy in patients with gastric cancer. Int J Clin Oncol. 2008;13(2):132-137. doi:10.1007/s10147-007-0727-1.

74. Kim W, Kim H-H, Han S-U, et al. Decreased Morbidity of Laparoscopic Distal Gastrectomy Compared With Open Distal Gastrectomy for Stage I Gastric Cancer. Ann Surg. 2015;XX(X):1. doi:10.1097/SLA.0000000000001346.

For peer review only - http://bmjopen.bmj.com/site/about/guidelines.xhtml

75. Kim H-H, Hyung WJ, Cho GS, et al. Morbidity and mortality of laparoscopic gastrectomy versus open gastrectomy for gastric cancer: an interim report--a phase III multicenter, prospective, randomized Trial (KLASS Trial). Ann Surg. 2010;251(3):417-420. doi:10.1097/SLA.0b013e3181cc8f6b.

76. Imamura H, Kurokawa Y, Kawada J, et al. Influence of bursectomy on operative morbidity and mortality after radical gastrectomy for gastric cancer: Results of a randomized controlled trial. World J Surg. 2011;35(3):625-630. doi:10.1007/s00268-010-0914-5.

77. Cai J, Wei D, Gao CF, Zhang CS, Zhang H, Zhao T. A prospective randomized study comparing open versus laparoscopy-assisted D2 radical gastrectomy in advanced gastric cancer. Dig Surg. 2012;28(5-6):331-337. doi:10.1159/000330782.

78. Chen Hu J, Xin Jiang L, Cai L, et al. Preliminary experience of fast-track surgery combined with laparoscopy-assisted radical distal gastrectomy for gastric cancer. J Gastrointest Surg. 2012;16(10):1830-1839. doi:10.1007/s11605-012-1969-4.

79. Takiguchi S, Fujiwara Y, Yamasaki M. Laparoscopy-Assisted Distal Gastrectomy Versus Open Distal. World J Surg. 2013;37(10):2379-2386. doi:10.1007/s00268-013-2121-7.

80. Lee J-H, Son S-Y, Lee CM, Ahn SH, Park DJ, Kim H-H. Morbidity and mortality after laparoscopic gastrectomy for advanced gastric cancer: Results of a phase II clinical trial. Surg Endosc Other Interv Tech. 2013;27(8):2877-2885. doi:10.1007/s00464-013-2848-0.

81. Sakuramoto S, Yamashita K, Kikuchi S, et al. Laparoscopy versus open distal gastrectomy by expert surgeons for early gastric cancer in Japanese patients: short-term clinical outcomes of a randomized clinical trial. Surg Endosc. 2013;27(5):1695-1705. doi:10.1007/s00464-012-2658-9.

82. Aoyama T, Yoshikawa T, Hayashi T, et al. Randomized comparison of surgical stress and the nutritional status between laparoscopy-assisted and open distal gastrectomy for gastric cancer. Ann Surg Oncol. 2014;21(6):1983-1990. doi:10.1245/s10434-014-3509-9.

For peer review only

83. Hirao M, Kurokawa Y, Fujita J, et al. Long-term outcomes after prophylactic bursectomy in patients with resectable gastric cancer: Final analysis of a multicenter randomized controlled trial. Surgery. 2015;157(6):1099-1105. doi:10.1016/j.surg.2014.12.024.

84. Galizia G, Lieto E, De Vita F, et al. Modified versus standard D2 lymphadenectomy in total gastrectomy for nonjunctional gastric carcinoma with lymph node metastasis. Surg (United States). 2015;157(2):285-296. doi:10.1016/j.surg.2014.09.012.

85. Hu Y, Huang C, Sun Y, et al. Morbidity and Mortality of Laparoscopic Versus Open D2 Distal Gastrectomy for Advanced Gastric Cancer: A Randomized Controlled Trial. J Clin Oncol. 2016. doi:10.1200/JCO.2015.63.7215.

86. Avery KNL, Chalmers KA, Brookes ST, et al. Development of a Core Outcome Set for Clinical Effectiveness Trials in Esophageal Cancer Resection Surgery. Ann Surg. March 2017:1. doi:10.1097/SLA.0000000000002204.

87. Database Search - Core Outcome Measures in Effectiveness Trials Initiative (COMET). http://www.comet-initiative.org/studies/search. Accessed May 19, 2016.

For peer review only

For peer review only - http://bmjopen.bmj.com/site/about/guidelines.xhtml

Figure Legends

Figure 1. Study selection and inclusion.

Figure 2. Outcome themes reported in a) gastric cancer surgery trials and b) in future trials based on study protocols.

For peer review only

For peer review only - http://bmjopen.bmj.com/site/about/guidelines.xhtml

Figure 1. Study selection and inclusion.

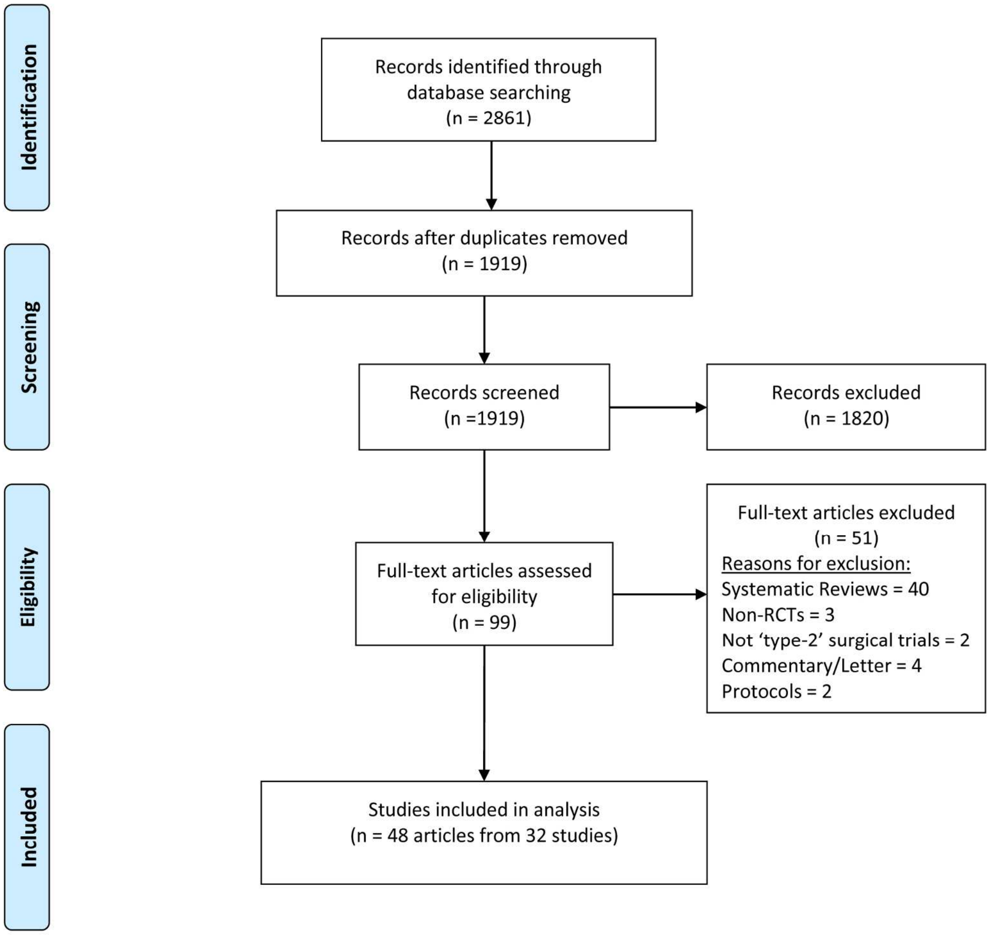

Figure 1. Study selection and inclusion.

185x192mm (300 x 300 DPI)

Figure 2. Outcome themes reported in a) gastric cancer surgery trials and b) in future trials based on study protocols.

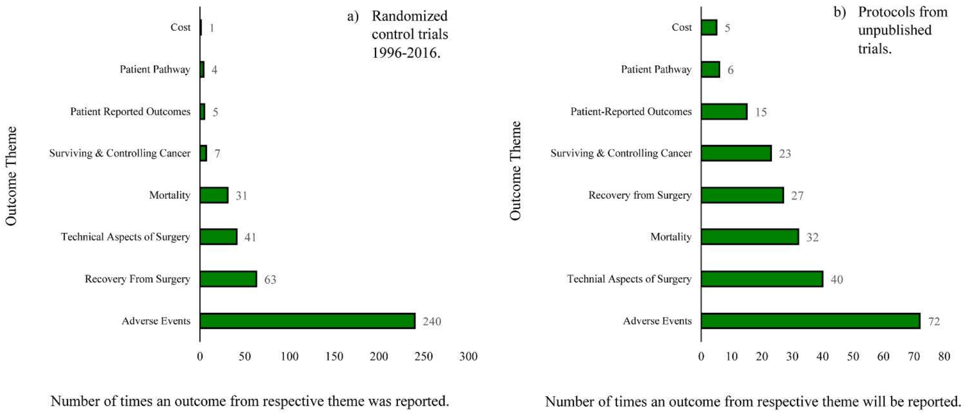

Figure 2. Outcome themes reported in a) gastric cancer surgery trials and b) in future trials based on study protocols.

258x126mm (300 x 300 DPI)

For peer review only - http://bmjopen.bmj.com/site/about/guidelines.xhtml

Appendix 1. Search Algorithm

Database: Ovid MEDLINE (R)
Search Strategy:
----
1 (Gastr$ or stomach$ or oesophagogastric junction or esophagogastric junction).mp. (645201)
2 exp Stomach/ (112711)
3 adenocarcinoma/ or neoplasms/ (457904)
4 1 or 2 (648680)
5 3 and 4 (37749)
6 exp Stomach Neoplasms/ (81208)
7 5 or 6 (100682)
8 chemoradiotherapy/ or chemotherapy, adjuvant/ or consolidation chemotherapy/ or antineoplastic combined chemotherapy protocols/ or induction chemotherapy/ (140865)
9 (antineoplast$ or antitumor$ or anti-tumor$ or anti-neoplast$ or chemotherp$).mp. (454727)
10 exp Gastrectomy/ or endoscopy, gastrointestinal/ or duodenoscopy/ or esophagoscopy/ or gastroscopy/ (69973)
11 (gastrectom$ or duodenoscop$ or esophagoscop$ or oesophagoscop$ or gastroscop$).mp. [mp=title, abstract, original title, name of substance word, subject heading word, keyword heading word, protocol supplementary concept word, rare disease supplementary concept word, unique identifier] (66259)
12 radiotherapy/ or brachytherapy/ or radioimmunotherapy/ or radiotherapy, adjuvant/ (74386)
13 (radiotherap$ or chemoradiotherap$ or chemo-radiotherap$ or "radiation therap$" or bracytherap$ or irradiat$).mp. (366760)
14 8 or 9 or 10 or 12 or 13 (854226)
15 7 and 14 (32194)
16 randomi?ed controlled trial.pt. (416592)
17 controlled clinical trial.pt. (92207)
18 randomized.ab. (308489)
19 randomly.ab. (218498)
20 exp animals/ not humans.sh. (4150916)
21 16 or 17 or 18 or 19 (743790)
22 21 not 20 (673762)
23 15 and 22 (2255)
24 limit 23 to yr="1996 -Current" (1806)
25 (Gastr$ or stomach$ or oesophagogastric junction or esophagogastric junction).mp. (645201)
26 exp Stomach/ (112711)
27 adenocarcinoma/ or neoplasms/ (457904)
28 25 or 26 (648680)
29 27 and 28 (37749)
30 exp Stomach Neoplasms/ (81208)
31 29 or 30 (100682)
32 chemoradiotherapy/ or chemotherapy, adjuvant/ or consolidation chemotherapy/ or antineoplastic combined chemotherapy protocols/ or induction chemotherapy/ (140865)
33 (antineoplast$ or antitumor$ or anti-tumor$ or anti-neoplast$ or chemotherp$).mp. (454727)
34 exp Gastrectomy/ or endoscopy, gastrointestinal/ or duodenoscopy/ or esophagoscopy/ or gastroscopy/ (69973)
35 (gastrectom$ or duodenoscop$ or esophagoscop$ or oesophagoscop$ or gastroscop$).mp. [mp=title, abstract, original title, name of substance word, subject heading word, keyword heading word, protocol supplementary concept word, rare disease supplementary concept word, unique identifier] (66259)
36 radiotherapy/ or brachytherapy/ or radioimmunotherapy/ or radiotherapy, adjuvant/ (74386)
37 (radiotherap$ or chemoradiotherap$ or chemo-radiotherap$ or "radiation therap$" or bracytherap$ or irradiat$).mp. (366760)
38 32 or 33 or 34 or 36 or 37 (854226)
39 31 and 38 (32194)
40 randomi?ed controlled trial.pt. (416592)
41 controlled clinical trial.pt. (92207)

For peer review only

42 randomized.ab. (308489)
43 randomly.ab. (218498)
44 exp animals/ not humans.sh. (4150916)
45 40 or 41 or 42 or 43 (743790)
46 45 not 44 (673762)
47 39 and 46 (2255)
48 limit 47 to yr="1996 -Current" (1806)

For peer review only

Appendix 2. Outcomes reported in 48 publications from 32 gastric cancer surgery trials.

Theme	Original Outcome	Frequency Reported
Adverse Events	Abdominal abscess	10
Adverse Events	Abdominal distention	1
Adverse Events	Abdominal drainage	1
Adverse Events	Abdominal liquid accumulation	1
Adverse Events	Abscess intra-abdominal	7
Adverse Events	Abscess subphrenic	1
Adverse Events	Acute enteritis	1
Adverse Events	Acute urinary retention	1
Adverse Events	Adverse drug reaction	1
Adverse Events	Afferent loop syndrome	2
Adverse Events	Allogenic blood transfusion	1
Adverse Events	Amylase level in drainage fluid	1
Adverse Events	Anastomosis failure	1
Adverse Events	Anastomosis stricture	2
Adverse Events	Anastomotic bleeding	1
Adverse Events	Anastomotic dehiscence	2
Adverse Events	Anastomotic leak	17
Adverse Events	Anastomotic leakage from GJ	1
Adverse Events	Anastomotic leakage from OJ	1
Adverse Events	Anastomotic leakage type 1	1
Adverse Events	Anastomotic leakage type 2	1
Adverse Events	Anastomotic stenosis	5
Adverse Events	Any complication	2
Adverse Events	ARDS	1

For peer review only

Adverse Events	Arteriosclerosis obliterans of the leg	1
Adverse Events	Ascites	2
Adverse Events	Atelectasis	2
Adverse Events	Atelectasis or pleural effusion	1
Adverse Events	Atrial fibrillation	1
Adverse Events	Bleeding	4
Adverse Events	Bleeding abdominal	1
Adverse Events	Bleeding from anastomosis	1
Adverse Events	Blood transfusion	2
Adverse Events	Blood transfusion volume	2
Adverse Events	Body temperature exceeding 37 ° C (days)	2
Adverse Events	Bowel obstruction	1
Adverse Events	Bowel obstruction/ileus	1
Adverse Events	Bronchopneumonia	1
Adverse Events	Bronchoscopic toilet	1
Adverse Events	Cardiac complications	6
Adverse Events	Cardiac failure	1
Adverse Events	Cardiocirculatory	1
Adverse Events	Cardiopulmonary disaese	1
Adverse Events	Catheter-induced sepsis	1
Adverse Events	Cerebrovascular	1
Adverse Events	Cholecystitis	3
Adverse Events	Cholecystitis acute	1
Adverse Events	Cholecystitis requiring percutaneous drainage	1
Adverse Events	Chyle leakage	2
Adverse Events	Chylous drainage	1
Adverse Events	Chylous lymphorrhea	1

For peer review only

Adverse Events	Colonic perforation	1
Adverse Events	Complications	3
Adverse Events	Complications after discharge	1
Adverse Events	Complications number of	1
Adverse Events	Deep vein thrombosis	3
Adverse Events	Delayed gastric emptying	2
Adverse Events	Delayed gastric emptying without obstruction	1
Adverse Events	Diarrhoea	1
Adverse Events	Drug-induced hepatitis	1
Adverse Events	Dumping syndrome	1
Adverse Events	Duodenal leak	1
Adverse Events	Duodenal stump leak	3
Adverse Events	Early dumping syndrome	1
Adverse Events	Early surgical complications	1
Adverse Events	Empyema thoracis	1
Adverse Events	Endocrine complications	1
Adverse Events	Endocrine events	1
Adverse Events	Enterocutaneous fistula	3
Adverse Events	Esophagus and remnant stomach infarction	1
Adverse Events	Fever	1
Adverse Events	Fluid collection	1
Adverse Events	Fluid collection/abscesses	1
Adverse Events	Gastric atonia	1
Adverse Events	Gastric remnant necrosis	1
Adverse Events	Gastrointestinal bleeding	1
Adverse Events	Gastrointestinal complications	1
Adverse Events	Gastrointestinal injury	1

For peer review only

Adverse Events	Gastroparesis	3
Adverse Events	Haemorrhage	2
Adverse Events	Hb	2
Adverse Events	Hepatic complications	2
Adverse Events	Hepatic failure	1
Adverse Events	Herpes zoster	1
Adverse Events	Hiccups	1
Adverse Events	Hospital morbidity	1
Adverse Events	Hypercapnia	1
Adverse Events	Iatrogenic spleen injury	1
Adverse Events	Idiopathic small bowel perforation	1
Adverse Events	Iileus mechanical	1
Adverse Events	Ileus	2
Adverse Events	Ileus adhesive	2
Adverse Events	Ileus paralytic	1
Adverse Events	Ileus prolonged	1
Adverse Events	Incision fat liquefaction	1
Adverse Events	Incision infection	1
Adverse Events	Infection	1
Adverse Events	Intestinal fistula	2
Adverse Events	Intestinal ischaemia	1
Adverse Events	Intestinal obstruction	3
Adverse Events	Intra-abdominal bleeding	3
Adverse Events	Intra-abdominal collections	1
Adverse Events	Intra-abdominal complications	1
Adverse Events	Intra-abdominal infection	1
Adverse Events	Intraluminal bleeding	3

For peer review only

Adverse Events	Intraoperative blood transfusion	2
Adverse Events	Intraoperative complications	3
Adverse Events	intraoperative major bleeding	1
Adverse Events	Intraperitoneal haemorrhage	1
Adverse Events	Late surgical complications	1
Adverse Events	Leakage	1
Adverse Events	Liver dysfunction	1
Adverse Events	Local complications	1
Adverse Events	Long-term complications	1
Adverse Events	Lung Infection	1
Adverse Events	Lymphatic leakage	1
Adverse Events	Lymphorrhoea	1
Adverse Events	Major abdominal infections	1
Adverse Events	Major cardiorespiratory incidents	1
Adverse Events	Major complications	2
Adverse Events	Major post-operative complication	1
Adverse Events	Major surgical complications	1
Adverse Events	Malabsorption	1
Adverse Events	Mediastinitis	1
Adverse Events	Medical complications	1
Adverse Events	Metabolic complications	1
Adverse Events	Minor complications	1
Adverse Events	Minor discharge of pancreatic juice	1
Adverse Events	Minor leakage	1
Adverse Events	Minor patchy pulmonary collapse	1
Adverse Events	Minor pulmonary atelectasis	2
Adverse Events	Morbidity	8

For peer review only

Adverse Events	Morbidity rate	1
Adverse Events	Multiple organ failure	1
Adverse Events	Myocardial infarction	1
Adverse Events	Nausea	1
Adverse Events	Need for blood transfusion	1
Adverse Events	Non-surgical Complications	6
Adverse Events	Number of patients with complications	1
Adverse Events	Operative complications	1
Adverse Events	Operative morbidity	3
Adverse Events	Other complications	7
Adverse Events	Overall complications	2
Adverse Events	Overall Post-operative complications	1
Adverse Events	Pancreas-related complications	1
Adverse Events	Pancreatic fistula	15
Adverse Events	Pancreatic injury	2
Adverse Events	Pancreatic leak	4
Adverse Events	Pancreatitis	1
Adverse Events	Pancreatitis acute	3
Adverse Events	Pancreatitis edematous	1
Adverse Events	Pancreatitis severe	1
Adverse Events	Pancreatitis traumatic	1
Adverse Events	Peri-operative complications	1
Adverse Events	Peritoneal haemorrhage	1
Adverse Events	Pleural	1
Adverse Events	Pleural effusion	5
Adverse Events	Pleural fluid	1
Adverse Events	Pneumonia	11

For peer review only

Adverse Events	Post-operative bleeding	1
Adverse Events	Post-operative complications	5
Adverse Events	Post-operative drain discharge	1
Adverse Events	Post-operative glucose tolerance	1
Adverse Events	Post-operative hemorrhage	2
Adverse Events	Post-operative major complications	1
Adverse Events	Post-operative minor complication	1
Adverse Events	Post-operative morbidity	1
Adverse Events	Post-operative psychosis	1
Adverse Events	Post-operative respiratory care	1
Adverse Events	Post-operative respiratory function	1
Adverse Events	Post-operative surgical parameters	1
Adverse Events	Post-operative symptoms	1
Adverse Events	Presence of gallstones	1
Adverse Events	Procedure-related morbidity and mortality	1
Adverse Events	Prolonged diarrhea	1
Adverse Events	Prolonged retention of intra-abdominal fluid	1
Adverse Events	Pulmonary	4
Adverse Events	Pulmonary complications	4
Adverse Events	Pulmonary edema	1
Adverse Events	Pulmonary embolism	2
Adverse Events	Pulmonary infection	1
Adverse Events	Pyothorax	1
Adverse Events	Rate of reinsertion of NG tube	1
Adverse Events	Recurrent laryngeal nerve palsy	1
Adverse Events	Reflux oesophagitis	2
Adverse Events	Re-laparotomy	2

For peer review only

Adverse Events	Renal complications	2
Adverse Events	Renal failure	2
Adverse Events	Re-operation	8
Adverse Events	Re-operation details	1
Adverse Events	Respirator use after surgery	1
Adverse Events	Respiratory complications	1
Adverse Events	Respiratory failure	1
Adverse Events	Return to theatre	1
Adverse Events	Septic complications	1
Adverse Events	Serious and potentially fatal complications	1
Adverse Events	Severe diarrhoea	1
Adverse Events	Severe feeding problem requiring prolonged hyperalimentation	1
Adverse Events	Severity of complications	2
Adverse Events	Severity of post-operative complications	1
Adverse Events	Short-term complications	1
Adverse Events	Small-bowel obstruction	1
Adverse Events	Splenic artery pseudoaneurysm	1
Adverse Events	Splenic injury	1
Adverse Events	Stasis	1
Adverse Events	Stenosis	1
Adverse Events	Surgical complications	8
Adverse Events	Surgical risk	1
Adverse Events	Systemic complications	1
Adverse Events	Systemic infections	1
Adverse Events	Thermal injury	1
Adverse Events	Thoracic effusion requiring thoracic drainage	1

For peer review only

Adverse Events	Thromboembolic complications	1
Adverse Events	Total complications	1
Adverse Events	Total major complications	1
Adverse Events	Total morbidity	4
Adverse Events	Tracheotomy	1
Adverse Events	Transfusion	3
Adverse Events	Transfusions received	1
Adverse Events	Transient ischemic attack	1
Adverse Events	Transient LFT abnormality	1
Adverse Events	Trocar related injury	1
Adverse Events	Tube tracheotomy	1
Adverse Events	Uncomplicated calf vein thrombosis	1
Adverse Events	Upper gastro-intestinal haemorrhage	1
Adverse Events	Urinary complications	2
Adverse Events	Urinary retention	1
Adverse Events	Urinary tract complications	1
Adverse Events	Urinary tract infection	3
Adverse Events	Viral infection	1
Adverse Events	Vomiting	1
Adverse Events	Wound abscess	1
Adverse Events	Wound complications	2
Adverse Events	Wound dehiscence	2
Adverse Events	Wound evisceration	1
Adverse Events	Wound haematoma	1
Adverse Events	Wound infection	10
Adverse Events	Wound infection/dehiscence	1
Adverse Events	Wound problem	1

For peer review only

Adverse Events	Wound seroma	2
Cost	Medical cost	1
Mortality	Death	8
Mortality	Death from a post-operative complication	1
Mortality	Death from all causes	1
Mortality	Death from gastric cancer as a cause	1
Mortality	Disease free survival	4
Mortality	Disease free survival 4-year	2
Mortality	Disease free survival 5-year	3
Mortality	Disease specific survival	1
Mortality	Disease specific survival 5-year	1
Mortality	Gastric cancer related deaths	1
Mortality	Hospital death	3
Mortality	Hospital mortality	7
Mortality	In-hospital mortality	2
Mortality	Mortality not related to surgery	1
Mortality	Operative death	1
Mortality	Operative mortality	3
Mortality	Overall survival	8
Mortality	Overall survival 10-year	1
Mortality	Overall survival 3-year	2
Mortality	Overall survival 5-year	6
Mortality	Overall survival 6-year	1
Mortality	Overall survival 7-year	1
Mortality	Post-operative death	4
Mortality	Post-operative mortality	3
Mortality	Post-operative survival	1

For peer review only

Mortality	Recurrence-free survival	5
Mortality	Relapse-free survival	1
Mortality	Survival 11-year	1
Mortality	Survival 5-year	11
Mortality	Survival Period	1
Mortality	Treatment related deaths	1
Patient Pathway	Days of hospitalization	1
Patient Pathway	Duration of hospital stay	12
Patient Pathway	Duration of post-operative hospital stay	11
Patient Pathway	Readmission	1
Patient Reported Outcomes	Degree of pain	1
Patient Reported Outcomes	Overall satisfaction	1
Patient Reported Outcomes	Pain	1
Patient Reported Outcomes	Post-operative Pain	1
Patient Reported Outcomes	QoL	3
Recovery From Surgery	4-day post-operative use of analgesics	1
Recovery From Surgery	Blood urea nitrogen	1
Recovery From Surgery	Body weight	1
Recovery From Surgery	CK	1
Recovery From Surgery	CRP	4
Recovery From Surgery	CRP 3 days after surgery	1
Recovery From Surgery	Days of fever	1
Recovery From Surgery	Days to sips of water	1
Recovery From Surgery	Decrease in body weight	1
Recovery From Surgery	Decrease of body weight 1 month after the operation	1
Recovery From Surgery	Dose of analgesic (mg)	1
Recovery From Surgery	Duration of pain control	1

For peer review only

Recovery From Surgery	Eating	1
Recovery From Surgery	FEV1(L)	1
Recovery From Surgery	FEVC(L)	1
Recovery From Surgery	Fever	1
Recovery From Surgery	First eating (post-operative day)	1
Recovery From Surgery	First walking (post-operative day)	1
Recovery From Surgery	Food intake	1
Recovery From Surgery	Frequency of analgesics injection	1
Recovery From Surgery	Frequency of injection given according to analgesic requests	1
Recovery From Surgery	IL-6	1
Recovery From Surgery	Immediate postoperative inflammatory and immune responses	1
Recovery From Surgery	Immunological response to surgery	2
Recovery From Surgery	Lean body mass	1
Recovery From Surgery	LFT	1
Recovery From Surgery	Number of days to get out of bed	1
Recovery From Surgery	Nutritional Status	1
Recovery From Surgery	Pain control	2
Recovery From Surgery	Post-operative analgesia	1
Recovery From Surgery	Post-operative course	1
Recovery From Surgery	Post-operative pain	1
Recovery From Surgery	Post-operative recovery	1
Recovery From Surgery	Prealbumin	1
Recovery From Surgery	Progression of oral intake	1
Recovery From Surgery	Pulmonary function	1
Recovery From Surgery	Recovery of Physical Activity	1
Recovery From Surgery	Residual pain at day 7	1

For peer review only

Recovery From Surgery	SaO2	1
Recovery From Surgery	Serum Albumin	1
Recovery From Surgery	Surgical stress response	1
Recovery From Surgery	The early recovery course	1
Recovery From Surgery	Time of first flatus/index of peristalsis recovery	1
Recovery From Surgery	Time to ambulation	1
Recovery From Surgery	Time to first flatus	4
Recovery From Surgery	Time to first flatus (days)	5
Recovery From Surgery	Time to first liquid intake	1
Recovery From Surgery	Time to first soft diet uptake	1
Recovery From Surgery	Time to food intake	1
Recovery From Surgery	Time to liquid diet	1
Recovery From Surgery	Time to removal of epidural anesthesia (days)	1
Recovery From Surgery	Time to sips of water	2
Recovery From Surgery	Time to start oral intake (days)	1
Recovery From Surgery	Time until removal of the naso-gastric tube	1
Recovery From Surgery	Time until start of meals	1
Recovery From Surgery	Times analgesic given	1
Recovery From Surgery	Times of pain rescue	1
Recovery From Surgery	Total amount of analgesics infused	1
Recovery From Surgery	Total body weight	1
Recovery From Surgery	Total protein	1
Recovery From Surgery	Walking	1
Recovery From Surgery	WBC	1
Recovery From Surgery	WCC	3
Surviving & Controlling Cancer	Cumulative risk of recurrence	2

For peer review only

Surviving & Controlling Cancer	Disease recurrence rate	1
Surviving & Controlling Cancer	Port site metastasis	2
Surviving & Controlling Cancer	Recurrence	4
Surviving & Controlling Cancer	Recurrence patterns	1
Surviving & Controlling Cancer	Recurrent disease	1
Surviving & Controlling Cancer	Tumor recurrence	1
Technical Aspects of Surgery	Amount of bleeding	1
Technical Aspects of Surgery	Blood loss	7
Technical Aspects of Surgery	Clear margin distance	1
Technical Aspects of Surgery	Conversion to open surgery	3
Technical Aspects of Surgery	Dissected Lymph nodes - mediastinal	1
Technical Aspects of Surgery	Dissected Lymph nodes - para-aortic	1
Technical Aspects of Surgery	Distal resection margin	2
Technical Aspects of Surgery	Duration of surgery	3
Technical Aspects of Surgery	Estimated blood loss	5
Technical Aspects of Surgery	Intraoperative blood loss	7
Technical Aspects of Surgery	Length of laparotomy incision	1
Technical Aspects of Surgery	Length of lesser curvature of resected stomach	1
Technical Aspects of Surgery	Length of lesser curvature of resected stomach	1
Technical Aspects of Surgery	Length of longest wound	1
Technical Aspects of Surgery	Length of resection on greater cureve	1

For peer review only

Technical Aspects of Surgery	Length of resection on lesser curve	1
Technical Aspects of Surgery	Main wound size	1
Technical Aspects of Surgery	Mean blood loss	2
Technical Aspects of Surgery	Mean operating time	1
Technical Aspects of Surgery	Number of lymph nodes dissected or resected or retrieved	22
Technical Aspects of Surgery	Number of lymph nodes removed N1 group	1
Technical Aspects of Surgery	Number of lymph nodes removed N2 group	1
Technical Aspects of Surgery	Number of lymph nodes removed N3 group	1
Technical Aspects of Surgery	Number of lymph nodes removed N4 group	1
Technical Aspects of Surgery	Operative blood loss	2
Technical Aspects of Surgery	Operative time	18
Technical Aspects of Surgery	Pathological outcomes	1
Technical Aspects of Surgery	Proximal margin positive/negative	1
Technical Aspects of Surgery	Proximal resection margin	2
Technical Aspects of Surgery	R0 resection	2
Technical Aspects of Surgery	Radicality R0	1
Technical Aspects of Surgery	Radicality R1	1
Technical Aspects of Surgery	Resection line involvement - distal	1
Technical Aspects of Surgery	Resection line involvement - proximal	1
Technical Aspects of Surgery	Residual Tumour	1
Technical Aspects of Surgery	Residual tumour R0	2
Technical Aspects of Surgery	Residual tumour R1/2	1
Technical Aspects of Surgery	Residual tumour R1/2	1
Technical Aspects of Surgery	Surgical time	2
Technical Aspects of Surgery	Time for operation	1
Technical Aspects of Surgery	Wound size	5

For peer review only

PRISMA 2009 Checklist

Section/topic	#	Checklist item	Reported on page #
**TITLE**			
Title	1	Identify the report as a systematic review, meta-analysis, or both.	1
**ABSTRACT**			
Structured summary	2	Provide a structured summary including, as applicable: background; objectives; data sources; study eligibility criteria, participants, and interventions; study appraisal and synthesis methods; results; limitations; conclusions and implications of key findings; systematic review registration number.	3
**INTRODUCTION**			
Rationale	3	Describe the rationale for the review in the context of what is already known.	6
Objectives	4	Provide an explicit statement of questions being addressed with reference to participants, interventions, comparisons, outcomes, and study design (PICOS).	7
**METHODS**			
Protocol and registration	5	Indicate if a review protocol exists, if and where it can be accessed (e.g., Web address), and, if available, provide registration information including registration number.	6
Eligibility criteria	6	Specify study characteristics (e.g., PICOS, length of follow-up) and report characteristics (e.g., years considered, language, publication status) used as criteria for eligibility, giving rationale.	8, table 1
Information sources	7	Describe all information sources (e.g., databases with dates of coverage, contact with study authors to identify additional studies) in the search and date last searched.	8,9
Search	8	Present full electronic search strategy for at least one database, including any limits used, such that it could be repeated.	App 1
Study selection	9	State the process for selecting studies (i.e., screening, eligibility, included in systematic review, and, if applicable, included in the meta-analysis).	9
Data collection process	10	Describe method of data extraction from reports (e.g., piloted forms, independently, in duplicate) and any processes for obtaining and confirming data from investigators.	10
Data items	11	List and define all variables for which data were sought (e.g., PICOS, funding sources) and any assumptions and simplifications made.	10
Risk of bias in individual studies	12	Describe methods used for assessing risk of bias of individual studies (including specification of whether this was done at the study or outcome level), and how this information is to be used in any data synthesis.	n/a
Summary measures	13	State the principal summary measures (e.g., risk ratio, difference in means).	n/a
Synthesis of results	14	Describe the methods of handling data and combining results of studies, if done, including measures of consistency (e.g., I^2) for each meta-analysis.	n/a

For peer review only - http://bmjopen.bmj.com/site/about/guidelines.xhtml

PRISMA 2009 Checklist

Section/topic	#	Checklist item	Reported on page #
Risk of bias across studies	15	Specify any assessment of risk of bias that may affect the cumulative evidence (e.g., publication bias, selective reporting within studies).	n/a
Additional analyses	16	Describe methods of additional analyses (e.g., sensitivity or subgroup analyses, meta-regression), if done, indicating which were pre-specified.	n/a
**RESULTS**			
Study selection	17	Give numbers of studies screened, assessed for eligibility, and included in the review, with reasons for exclusions at each stage, ideally with a flow diagram.	Figure 1
Study characteristics	18	For each study, present characteristics for which data were extracted (e.g., study size, PICOS, follow-up period) and provide the citations.	Table 2
Risk of bias within studies	19	Present data on risk of bias of each study and, if available, any outcome level assessment (see item 12).	n/a
Results of individual studies	20	For all outcomes considered (benefits or harms), present, for each study: (a) simple summary data for each intervention group (b) effect estimates and confidence intervals, ideally with a forest plot.	n/a
Synthesis of results	21	Present results of each meta-analysis done, including confidence intervals and measures of consistency.	n/a
Risk of bias across studies	22	Present results of any assessment of risk of bias across studies (see Item 15).	n/a
Additional analysis	23	Give results of additional analyses, if done (e.g., sensitivity or subgroup analyses, meta-regression [see Item 16]).	n/a
**DISCUSSION**			
Summary of evidence	24	Summarize the main findings including the strength of evidence for each main outcome; consider their relevance to key groups (e.g., healthcare providers, users, and policy makers).	30
Limitations	25	Discuss limitations at study and outcome level (e.g., risk of bias), and at review-level (e.g., incomplete retrieval of identified research, reporting bias).	31
Conclusions	26	Provide a general interpretation of the results in the context of other evidence, and implications for future research.	32
**FUNDING**			
Funding	27	Describe sources of funding for the systematic review and other support (e.g., supply of data); role of funders for the systematic review.	2

From: Moher D, Liberati A, Tetzlaff J, Altman DG, The PRISMA Group (2009). Preferred Reporting Items for Systematic Reviews and Meta-Analyses: The PRISMA Statement. PLoS Med 6(7): e1000097. doi:10.1371/journal.pmed1000097

For more information, visit: **www.prisma-statement.org**.

For peer review only - http://bmjopen.bmj.com/site/about/guidelines.xhtml

BMJ Open

Reporting of Outcomes in Gastric Cancer Surgery Trials: A Systematic Review.

Journal:	*BMJ Open*
Manuscript ID	bmjopen-2018-021796.R2
Article Type:	Research
Date Submitted by the Author:	06-Aug-2018
Complete List of Authors:	Alkhaffaf, Bilal; Manchester University NHS Foundation Trust, Department of Oesophago-Gastric Surgery<br>Blazeby, Jane; University of Bristol, Centre for Surgical Research<br>Williamson, Paula; MRC North West Hub for Trials Methodology Research, University of Liverpool<br>Bruce, Iain; Manchester University NHS Foundation Trust, Paediatric ENT Department<br>Glenny, Anne-Marie; University of Manchester, Division of Dentistry
**Primary Subject Heading**:	Surgery
Secondary Subject Heading:	Oncology, Evidence based practice, Research methods, Patient-centred medicine
Keywords:	Surgical Oncology, Stomach Neoplasms, Outcome Assessment, Delphi Technique, Patient Reported Outcome Measures, Outcome reporting

For peer review only

SCHOLARONE™ Manuscripts

Title Page

Reporting of Outcomes in Gastric Cancer Surgery Trials: A Systematic Review.

Authors

Bilal Alkhaffaf[1,2], Jane M Blazeby[3], Paula R Williamson[4], Iain A Bruce[5,6] & Anne-Marie Glenny[7]

Affiliations

[1]Department of Oesophago-Gastric Surgery, Manchester University NHS foundation Trust, Manchester Academic Health Science Centre, Manchester, UK

[2]Division of Molecular & Clinical Cancer, Faculty of Biology, Medicine and Health, University of Manchester

[3]Centre for Surgical Research, University of Bristol

[4]MRC North West Hub for Trials Methodology Research, University of Liverpool

[5]Paediatric ENT Department, Royal Manchester Children's Hospital, Manchester University NHS Foundation Trust, Manchester Academic Health Science Centre, Manchester, UK

[6]Division of Infection, Immunity and Respiratory Medicine, Faculty of Biology, Medicine and Health, University of Manchester, Manchester, UK

[7]Division of Dentistry, University of Manchester

Corresponding Author

Mr Bilal Alkhaffaf

Consultant Oesophago-Gastric Surgeon

Manchester University Hospitals NHS Foundation Trust

Manchester Royal Infirmary

Oxford Road

For peer review only

M13 9WL

E-mail: bilal.alkhaffaf@mft.nhs.uk

Telephone: +44 161 276 1234

Manuscript date: 09/05/2018

Reprints

Reprints will not be available from the authors.

Funding

This study is funded by the National Institute for Health Research (NIHR) Doctoral Research Fellowship Grant (DRF-2015-08-023).

Disclaimer

This paper presents independent research funded by the National Institute for Health Research (NIHR). The views expressed are those of the author(s) and not necessarily those of the NHS, the NIHR or the Department of Health.

Running Title

Reporting of Outcomes in Gastric Cancer Surgery Trials.

Word Count: 3,426

Number of tables: 5

Number of figures: 2

Number of appendices: 2

For peer review only

Structured Abstract

Background

The development of clinical guidelines for the surgical management of gastric cancer should be based on robust evidence from well-designed trials. Being able to reliably compare and combine the outcomes of these trials is a key factor in this process.

Objectives

To examine variation in outcome reporting by surgical trials for gastric cancer; to identify outcomes for prioritisation in an international consensus study to develop a core outcome set in this field.

Data Sources

Systematic literature searches (Evidence Based Medicine, MEDLINE, EMBASE, CINAHL, ClinicalTrials.gov and WHO ICTRP) and a review of study protocols of randomized controlled trials, published between 1996 and 2016.

Intervention

Therapeutic surgical interventions for gastric cancer. Outcomes were listed verbatim, categorized into groups (outcome themes) and examined for definitions and measurement instruments

Results

Of 1919 abstracts screened, 32 trials (9,073 participants) were identified. A total of 749 outcomes were reported of which 96 (13 per cent) were accompanied by an attempted definition. No single outcome was reported by all trials. 'Adverse events' was the most frequently reported 'outcome theme' in which 240 unique terms were described. 12 trials (38%) classified complications according to severity, with 5 (16%) using a formal classification system (Clavien-Dindo or Accordion scale). Of 27 trials which described 'short-term' mortality, 15 (47%) used one of 5 different definitions. Six out of the 32 trials (19%) described 'patient-reported outcomes'.

Conclusion

For peer review only - http://bmjopen.bmj.com/site/about/guidelines.xhtml

Reporting of outcomes in gastric cancer surgery trials is inconsistent. A consensus approach to develop a minimum set of well-defined, standardized outcomes to be used by all future trials examining therapeutic surgical interventions for gastric cancer is needed. This should consider the views of all key stakeholders, including patients.

KEYWORDS:

Surgical Oncology

Stomach Neoplasms

Outcome Assessment

Delphi Technique

Patient Reported Outcome Measures

Treatment Outcome

Outcome reporting

For peer review only

Article Summary

Strengths and Limitations of This Study

- This systematic review is the first to describe the variation in outcome reporting within the field of surgical trials for gastric cancer.
- The study is based on a reproducible and transparent methodology which has been subjected to critical appraisal during a peer-review process.
- The study forms part of a larger project (The GASTROS Study) to develop a 'core outcome set' (COS) for use in surgical trials for gastric cancer and was reviewed and funded by the National Institute of Health Research (UK).
- Only English-language and randomized studies were included in the analysis; Expanding the search may have resulted in the identification of other relevant outcomes reported in this field.

For peer review only

Main Article

Introduction

Background

Gastric cancer remains a leading cause of cancer-related death globally[1]. Long-term survival remains poor and has not improved significantly over the last four decades[2]. Whilst there has been a shift to multi-modal therapy over the last decade, surgery remains the primary method of curative treatment. Many developments in surgical techniques aim to improve long-term survival, whilst minimizing post-operative complications. Understanding which of these approaches are optimal for patients should be based on robust evidence from well-designed trials. This process involves the synthesis of evidence in the form of systematic reviews which can only be reliably undertaken if trials report the same outcomes and measure them in the same manner.

This review forms part of the first stage of a three-stage study, which intends to examine and address problems with inconsistent outcome reporting in gastric cancer surgery trials (GASTROS – **GA**stric Cancer **S**urgery **TR**ials **R**eported **O**utcome **S**tandardisation). The study aims to develop a 'core outcome set' (COS) – a minimum group of standardized and well-defined outcomes, relevant to key stakeholders and measured by all trials[3] – to standardize the reporting of outcomes in randomized control trials within this field. Our previously published study protocol contains an overview of all three stages[4].

Within our study protocol, we described the results from a 'rapid review' of gastric cancer surgery trials during a 24-month period which demonstrated significant variations in outcome reporting. We hypothesised that these variations were likely to represent a more widespread problem within this field. Inconsistencies in outcome reporting are prevalent within the medical literature and contribute significantly to 'research waste'[5]. Several reviews have demonstrated that trials within the same field often report different outcomes, define them poorly and use various outcome measurement instruments[6–9]. This results in data which cannot be reliably compared or combined leading to further confusion within the evidence base. As such, initiatives such as COMET (Core Outcome Measures in Effectiveness Trials) were formed to promote the development of COS to address these issues[3].

For peer review only - http://bmjopen.bmj.com/site/about/guidelines.xhtml

With respect to surgical trials for gastric cancer, a) no rigorous examination of outcome reporting has been previously undertaken and b) there is no COS for use in this field.

Aims & Objectives

This review aims demonstrate whether further work to develop a COS to be used in surgical trials for gastric cancer is required. Specifically, the objectives are:

1. to examine the degree of variation in the reporting of outcomes described by gastric cancer surgery trials.
2. to generate a 'long-list' of potentially important outcomes which will be prioritized during a Delphi survey in stage two of the study.

For peer review only

For peer review only - http://bmjopen.bmj.com/site/about/guidelines.xhtml

Methods

Definitions

The GASTROS study, and more specifically this review, focuses on outcome reporting in 'therapeutic surgical trials'. A 'surgical trial' has been previously defined as one of the following [10]:

- Type 1 - A trial of medical interventions in surgical patients
- Type 2 - A trial which compares a surgical intervention to another surgical intervention
- Type 3 - A trial which compares a surgical intervention to a non-surgical intervention

The GASTROS study focuses on 'type 2' trials due to the significant research activity within this field (a detailed justification can be found in our study protocol)[4]. In the context of gastric cancer, a 'therapeutic surgical intervention' is defined as a potentially curative procedure which aims to excise the gastric neoplasm resulting in partial or total organ loss.

Search strategy

A summary of the review's inclusion and exclusion criteria is summarised in table 1, with details of our search strategy presented below. An example search algorithm for the Medline via OVID database is presented in appendix 1.

Timeline

Trials were searched from 1996 when the first CONSORT statement for the reporting of RCTs was published up to and including March 2016. The COS aims to influence trial design regardless of the country of origin of participating centers and patients. Many of the surgical interventions (e.g. D2 lymphadenectomy) which have recently been examined in the West have long been established practice in the Far East and Asia. Searching trials over a 20-year period allows a comprehensive understanding of which outcomes have been measured for similar trials regardless of the trial location.

Identifying studies

Detailed search strategies were developed for each of the following electronic databases examined:

- Evidence Based Medicine Reviews via OVID

For peer review only - http://bmjopen.bmj.com/site/about/guidelines.xhtml

- Cochrane Database of Systematic Reviews 2005 to March 30, 2016,
- ACP Journal Club 1991 to March 2016,
- Database of Abstracts of Reviews of Effects 1st Quarter 2016
- Cochrane Central Register of Controlled Trials February 2016
- Health Technology Assessment 1st Quarter 2016
- NHS Economic Evaluation Database 1st Quarter 2016

- MEDLINE via OVID (January 1st 1996 to March 30, 2016)
- EMBASE via OVID (January 1st 1996 to March 30, 2016);
- CINAHL via EBSCO (January 1st 1996 to March 30, 2016).

In order to identify surgical interventions and outcome measures being used in current studies, we searched the following databases for protocols of ongoing trials, including completed trials not yet published:

- The US National Institutes of Health Trials Register (http://clinicaltrials.gov);
- The WHO International Clinical Trials Registry Platform (http://apps.who.int/trialsearch/default.aspx).

Non-English language studies were excluded from this review due to resource limitations. Trials published only as conference abstracts were excluded as they are often limited by 'word count' and hence the abstract would not represent a comprehensive list of outcomes measured in the respective study.

Assessment of eligibility

For quality assurance, two review authors (BA and AMG) independently screened the titles and abstracts retrieved from the electronic searches. This assessment was undertaken in groups of ten

For peer review only - http://bmjopen.bmj.com/site/about/guidelines.xhtml

abstracts in reverse chronological order. Once there was complete agreement with two consecutive groups of ten abstracts, the remaining abstracts were split and each reviewer screened independently. Full text copies of all study publications that appeared to meet the inclusion criteria were obtained. Full text copies were also obtained where there was insufficient information in the title or abstract to make a clear judgement. Systematic reviews of RCTs were also retrieved to find studies which had previously not been identified.

BA and AMG independently assessed the full text copies for eligibility. This assessment was undertaken in groups of ten publications in reverse chronological order. Once there was complete agreement with two consecutive groups of ten abstracts, the remaining publications were split, and each reviewer extracted data independently. Any disagreements were resolved through discussion. There were no unresolved disagreements that required referral to the GASTROS study management team for a final decision.

Data Extraction

BA and AMG independently reviewed all eligible publications and extracted data (described below) into a Microsoft Excel (Version 2013, Microsoft, Washington, DC, USA) spreadsheet.

Publication versus Study

It is not uncommon that investigators publish results at different stages of their trial and with each publication present a new set of outcomes. The GASTROS study team decided to amalgamate the outcomes published in all publications associated with a single trial to more fairly reflect outcomes being reported by research groups.

Trial Characteristics

The following data were recorded for each trial:

1. Author details

2. Title of publication

For peer review only - http://bmjopen.bmj.com/site/about/guidelines.xhtml

3. Journal

4. Year of publication

5. Number of participating centres

6. Country of 1st Author

7. Countries of participating centres

8. Total number of patients recruited to the study

9. Length of follow up

10. Interventions being investigated

Outcomes

We defined an outcome as 'a unique endpoint which attempts to describe health-related changes that occur secondary to a therapeutic intervention'[4]. The following data were recorded for each outcome:

1. Outcome measured (and whether stated as primary or secondary outcome). Where a primary outcome was not explicitly stated, the outcome on which the sample size calculation was based was taken as the primary outcome.

2. Whether the outcome was defined or not. Outcomes were considered defined if text of their meaning or a citation was provided.

3. The definition of the outcome.

4. The method of outcome measurement (indicators and/or tools used, if relevant).

5. Time points and time-period at or during which the outcome was measured (for example quality of life at 3-months post-surgery).

For peer review only - http://bmjopen.bmj.com/site/about/guidelines.xhtml

Merging Outcomes & Grouping Under 'Themes'

Outcomes were extracted verbatim from publications and minimal merging of terms was undertaken. Outcomes were merged to accommodate for variant spellings of the same words. For example, 'anastomotic leak', 'anastomotic leak**age**' and 'anastomotic leak**s**' were merged into 'anastomotic leak'. The verbatim texts and merged terms were verified and authorized respectively by the study management group.

From the experience of other groups undertaking reviews of outcome reporting, the resulting lists of outcomes are generally extremely long and unwieldy[6]. Consequently, developing a method to organize these outcomes has been necessary. The subject of taxonomy in outcome reporting, including hierarchical structure and which terms/definitions to use, is an emerging area of great significance. We set out our definitions a priori, which can be found in our study protocol[11]. Many COS developers have organized their outcomes into broad categories with common 'themes'. Our study is one of only a handful addressing outcome reporting in surgical trials related to the gastro-intestinal (GI) tract. At the time of data analysis, we opted to group outcomes under 'themes' (detailed in table 2) similar to those described by other surgical COS[6–8,12]. Doing so enables COS researchers to more readily understand trends in outcome reporting within the field of GI surgery. Whilst the themes used in our review enable the reader to understand the types of outcomes being reported, this system has not been developed through wider consensus and has not been subject to a validation process.

At the time of writing, a broader taxonomy for outcome classification had been proposed[13]. This system aims to address some of the ambiguity associated with outcome classification on a wider-scale and organizes outcomes under 38 'outcomes domains' which sit under 5 'outcomes areas' ('mortality/survival', 'physiological/clinical', 'life impact', 'resource use' and 'adverse events'). Whilst the authors have demonstrated that this system is comprehensive and applicable to trials irrespective of the field being studied, they have called for further validation of their work.

For peer review only - http://bmjopen.bmj.com/site/about/guidelines.xhtml

Patient & Public Involvement

A Study Advisory Group (SAG) forms part of the management structure of the wider GASTROS study[4], of which this review forms part of the first stage. The SAG is made up of key stakeholder representatives including patients, oncology nurses and surgeons. The group provides advice on the methodology of the study, general delivery of the study against its stated objectives, and ensures that the viewpoints of all stakeholder groups are considered. The results of this systematic review were presented to a SAG meeting; the ensuing discussion influenced certain aspects of the results section within this paper such as the emphasis on patient-reported outcomes.

For peer review only

For peer review only - http://bmjopen.bmj.com/site/about/guidelines.xhtml

Table 1. Inclusion and exclusion criteria for this review.

	Included	Excluded
Types of Studies	• Type 2* surgical randomized controlled trials (RCTs) and protocols of surgical RCTs (all trial phases).<br>• Systematic reviews of type 2 surgical RCTs.<br>• English Language studies.	• Type 1 or type 3* surgical RCTs and systematic reviews of type 1 or type 3 RCTs.<br>• Non-randomized studies.<br>• Non-English language studies.
Population	• Patients aged 18 years and over.	• Patients below the age of 18.
Interventions	• Partial or total gastrectomy.<br>• Surgery with curative intent.	• Esophagectomy for gastro-esophageal junctional tumors.<br>• Surgery with non-curative intent (i.e. in stage 4 cancer with prior expectation of an R1 or R2 resection) for the relief of symptoms such as gastric outlet obstruction or bleeding.<br>• Endoscopic interventions.
Conditions	• Invasive cancer of the stomach and gastro-esophageal junction.	• Dysplasia or non-invasive gastric neoplasms.<br>• Sarcoma (including gastrointestinal stromal tumors).<br>• Gastric lymphoma.

For peer review only - http://bmjopen.bmj.com/site/about/guidelines.xhtml

*Type 1 - a trial of medical interventions in surgical patients, type 2 - a trial which compares a surgical intervention to another surgical intervention, type 3 - a trial which compares a surgical intervention to a non-surgical intervention[10].

For peer review only - http://bmjopen.bmj.com/site/about/guidelines.xhtml

Table 2. Number of times at least one outcome from respective theme was reported in published trials and unpublished or actively recruiting trial protocols.

**Outcome Theme**	**Theme Definition**	Published Trials (n=32) (%)	Unpublished or Actively Recruiting Trial Protocols (n=23**) (%)
Cost	Relating to delivery of surgery as part of clinical care within a healthcare system.	1 (3)	5 (23)
Patient Pathway	Outcomes related to the flow of patients through the healthcare system (e.g. hospital stay, readmission).	20 (63)	4 (17)
Patient-Reported Outcomes	Outcomes taken from the patient perspective.*	6 (19)	13 (57)
Surviving & Controlling Cancer	Measures of disease recurrence or disease progression.	15 (47)	17 (74)
Mortality	Outcomes related to short and long-term survival/death rates and cause of death.	27 (84)	19 (83)
	Short-term mortality/peri-operative death.	27 (84)	10 (43)
	Long-term survival.	13 (41)	19 (44)
Technical Aspects of Surgery	Outcomes recorded directly in the operating theatre (e.g. operation time, blood loss).	31 (97)	13 (57)

For peer review only - http://bmjopen.bmj.com/site/about/guidelines.xhtml

Recovery from Surgery	Report of patient condition following surgery and the ability to return to preoperative or premorbid state.	16 (50)	9 (39)
Adverse Events	Forms of short- and long-term postoperative complications following surgery.	31 (97)	18 (78)

* Certain patient-reported outcomes may fall under other 'themes', e.g. 'post-operative pain' may relate to 'recovery from surgery'. ** One trial protocol contained no information about planned outcomes to report, therefore 23 out of total 24 trials were included in this table.

For peer review only

Results

Summary

A total of 1,919 abstracts were screened which resulted in the identification of 48 publications from 32 trials (having recruited a total of 9073 patients) eligible for inclusion in the review (figure 1). A further 875 protocols were screened which identified 24 active or unpublished trials aiming to recruit 10,761 patients. A summary of all trials included in the analysis is described in table 3. During the data extraction process, no disagreements requiring discussion in the study management group arose between the two independent reviewers. A total of 749 (392 unique terms) outcomes were reported of which 13% (96 out of 749) were accompanied by an attempted definition. Thirty-eight percent of trials (12 out of 32) described a primary outcome or provided a sample size calculation. No single outcome was reported in every trial.

Analysis of Outcomes According to Themes

Outcomes were organised into eight 'outcome themes', illustrated in figure 2 and described in table 2. A comprehensive list of reported outcomes is presented in appendix 2. Below, we present a summary of some of the most commonly reported short and long-term outcome themes.

Mortality

Death after surgery was generally described as 'short-term' and 'long-term' survival. Long-term survival was used as a primary outcome measure in 41% of trials (13 out of 32). The terms used to describe long-term mortality and the time-points at which they were measured was inconsistent (table 4). 'Short-term' mortality was reported by 84% of trials (27 out of 32) of which fifteen provided one of the following definitions (frequency each definition was used is presented in brackets):

1. 'Death within **30-days** of surgery' (3/15)
2. 'Death of **any cause** within **30 days**, or death within the **same hospitalization' (9/15)**
3. **'In-hospital** deaths and 'deaths' **within 1 month' (1/15)**
4. 'Death within **30 days** of the operation or during **any hospital stay' (1/15)**

For peer review only - http://bmjopen.bmj.com/site/about/guidelines.xhtml

5. **‘Any death** that occurred during the **hospital stay (1/15)**

Adverse Events

Adverse events were the commonest outcome theme to be reported and made up half of the ten most reported outcomes (table 5). ‘Anastomotic leak’ was the commonest adverse event to be reported and was described using 5 different definitions (frequency each definition was used is presented in brackets):

- ‘Clinical and radiological diagnosis’ (2).
- ‘Confirmed by gastrointestinal x-ray imaging, endoscopy, or angiography’ (1).
- ‘Dehiscence confirmed by radiographic examination using contrast medium’ (1).
- ‘Type I anastomotic leakage: a small localized leakage at the esophagojejunal anastomosis, without pleural or abdominal spillage, demonstrated by radiologic studies with barium’ (1).
- ‘Type II anastomotic leakage: an important dehiscence of the esophago-jejunal anastomosis, with pleural or abdominal dissemination, appearance of intestinal content through the drains, a positive methylene blue test (appearance of orally ingested methylene blue through the drains), and clear demonstration of this leakage by radiologic contrast studies’ (1).

Adverse events were categorized by 12 out of 32 trials using terms including ‘major’, ‘minor’, ‘short-term’ and ‘long-term’. Five trials used a formal classification system (Clavien-Dindo Classification or Accordian Severity Grading).

Patient Reported Outcomes

Patient-reported Outcomes (PROs) were reported in 19% of trials (6 out of 32) and included measures of quality of life (QoL) (n=3) and ‘pain’ (n=3). QoL was measured using validated tools for gastric cancer in two trials (EORTC QLQ-C30 with QLQ and Spitzer QoL Index) and a non-validated tool in one trial. Pain was measured using 3 different visual-analogue scales.

For peer review only - http://bmjopen.bmj.com/site/about/guidelines.xhtml

Multi-Centre Trials

Forty per cent (13 out of 32) of studies were multi-centre trials (table 2). Ninety-two percent (12 out of 13) of trials stated a primary outcome measure (5-year overall survival, 5-year survival, all-cause mortality and 3-year disease free survival). Sixteen percent (63 out of 393) of all outcomes reported in these trials were accompanied by an attempted definition. PRO's were reported in 23% of trials (3 out of 13) with 8% (1 out of 13) of studies reporting 'quality of life' as an outcome.

Findings from Study Protocols

Most of the 24 ongoing or unpublished trials [14–37] are recruiting in China (n=13), with twenty examining 'extent of lymphadenectomy' or minimally invasive approaches to surgery. A total of 220 uniquely termed outcomes are planned to be reported, thirty-five of which (16%) have an accompanying definition in the respective protocol. The commonest term used to report 'long-term survival' is 'overall survival' (OS) which will be measured by sixteen trials. Seven of these trials plan to measure OS after 5-years of follow-up, three at 3-years of follow-up, and six did not identify time points at which OS would be measured. At the time of our search, one trial protocol contained no information about which outcomes are to be measured.

QoL is due to be measured by ten trials (42 per cent) with five trials proposing to use one or a combination of four different measurement instruments (EORTC QLQ-C30 with QLQ-STO22, SF-36, GIQLI and Euro-Quality of Life-5D). Seven protocols described the timing of the quality of life measurements as follows:

- 'Preoperative, postoperative **3 weeks** and postoperative **12 months'.**
- 'In **pre-therapy <7 days**, **pre-operative <7 days**, and post-operative at **12 months** after surgery'.
- **'90th postoperative day'.**
- **'Regularly** for three years after surgery'.

For peer review only - http://bmjopen.bmj.com/site/about/guidelines.xhtml

- **‘Preoperatively**, **five days postoperatively**, **three** months, **six** months and **one year** postoperatively’.
- **‘Baseline**, **1 week**, **1** month, **6**-month, **1 year**, **3 years’.**
- **‘6 weeks**, **12**, **24**, **36**, **48** and **50 months** after surgery’.

For peer review only

For peer review only - http://bmjopen.bmj.com/site/about/guidelines.xhtml

Table 3. Published gastric cancer surgery trials included in the study analysis.

Trial Reference(s)	Trial Number	Author	Year of 1st Publication	Participating Countries	Number of Recruiting Sites	Patients Recruited	Interventions
38–41	1	Hartgrink et al, Bonenkamp et al., Sasako et al., Songun et al.	1995	Netherlands	80	1078	• D1 lymphadenectomy<br>• D2 lymphadenectomy
42,43	2	Cuschieri et al.	1996	UK	32 surgeons*	737	• D1 lymphadenectomy<br>• D2 lymphadenectomy
44,45	3	Marubini et al. Bozzetti et al.	1999	Italy	31	615	• D2 subtotal gastrectomy<br>• D2 total gastrectomy
46	4	Maeta et al	1999	Japan	1	70	• D3 total gastrectomy<br>• D4 total gastrectomy
47	5	Furukawa et al.	2000	Japan	1	110	• Total gastrectomy and distal pancreatectomy

For peer review only - http://bmjopen.bmj.com/site/about/guidelines.xhtml

							• Pancreas preserving total gastrectomy
48	6	Csendes et al.	2002	Chile	1	187	• D2 total gastrectomy with splenectomy<br>• Spleen preserving D2 total gastrectomy
49	7	Kitano et al.	2002	Japan	1	28	• Laparoscopic assisted distal gastrectomy<br>• Open distal gastrectomy
50	8	Fujii et al	2003	Japan	1	20	• Laparoscopic assisted distal gastrectomy<br>• Open distal gastrectomy
51–53	9	Degiuili et al	2004	Italy	5	267	• D1 lymphadenectomy<br>• D2 lymphadenectomy
54–57	10	Sano et al., Sasako et al., Kodera et al, Tsujinaka et al.	2004	Japan	24	523	• D2 lymphadenectomy<br>• D2 lymphadenectomy & para-aortic node dissection

For peer review only - http://bmjopen.bmj.com/site/about/guidelines.xhtml

58	11	Shibata et al	2004	Japan	9	81	• Pylorus-preserving gastrectomy<br>• Distal gastrectomy
59	12	Inaba et el.	2004	Japan	1	410	• Midline laparotomy<br>• Transverse laparotomy
60	13	Lee et al.	2005	South Korea	1	47	• Laparoscopic assisted D2 distal gastrectomy<br>• Open D2 distal gastrectomy
61	14	Hayashi et al	2005	Japan	1	28	• Laparoscopic assisted distal gastrectomy with extra-perigastric node dissection.<br>• Open distal gastrectomy with extra-perigastric node dissection.
62	15	Huscher et al	2005	Italy	1	59	• Laparoscopic sub-total gastrectomy<br>• Open sub-total gastrectomy
63–66	16	Wu et al.	2006	Taiwan	1	221	• D1 lymphadenectomy<br>• D3 lymphadenectomy
67,68	17	Sasako et al.	2006	Japan	27	167	• Left Thoracoabdominal approach

For peer review only - http://bmjopen.bmj.com/site/about/guidelines.xhtml

		Kurokawa et al.					• Abdominal trans-hiatal approach
69	18	Yu et al	2006	South Korea	1	207	• Total gastrectomy with splenectomy<br>• Spleen preserving total gastrectomy
70	19	Kulig et al.	2007	Poland	6	275	• D2 lymphadenectomy<br>• D2 lymphadenectomy & para-aortic node dissection
71,72	20	Kim et al.	2008	South Korea	1	164	• Laparoscopic assisted distal gastrectomy<br>• Open distal gastrectomy
73	21	Yonemura et al.	2008	Japan, Taiwan, South Korea	10	269	• D2 lymphadenectomy<br>• D4 lymphadenectomy
74,75	22	Kim et al.	2010	South Korea	13	1416	• Laparoscopic assisted D2 distal gastrectomy<br>• Open distal D2 gastrectomy
76	23	Imamura et al	2011	Japan	11	210	• D2 gastrectomy and bursectomy

For peer review only - http://bmjopen.bmj.com/site/about/guidelines.xhtml

							• D2 gastrectomy without bursectomy
77	24	Cai et al.	2012	China	1	123	• Laparoscopic assisted distal gastrectomy<br>• Open distal gastrectomy
78	25	Chen Hu et al.	2012	China	1	88	• Laparosopic gastrectomy<br>• Open gastrectomy<br>• Standard post-operative protocol<br>• Fast-track post-operative protocol
79	26	Takiguchi	2013	Japan	1	40	• Laparoscopic assisted distal gastrectomy<br>• Open distal gastrectomy
80	27	Lee et al.	2013	South Korea	1	204	• D2 distal gastrectomy<br>• D2 total gastrectomy
81	28	Sakuramoto et al	2013	Japan	1	64	• Laparoscopic assisted distal gastrectomy<br>• Open distal gastrectomy
82	29	Aoyama et al	2014	Japan	1	26	• Laparoscopic assisted distal

For peer review only

							gastrectomy<br>• Open distal gastrectomy
83	30	Hirao et al.	2015	Japan	11	210	• D2 gastrectomy with bursectomy<br>• D2 gastrectomy without bursectomy
84	31	Galizia et al	2015	Italy	1	73	• D1'+' total gastrectomy<br>• D2 total gastrectomy
85	32	Hu et al.	2016	China	14	1056	• Laparoscopic assisted D2 distal gastrectomy<br>• Open D2 distal gastrectomy

*Number of sites not specified.

For peer review only

Table 4. Reporting of 'long-term mortality' in gastric cancer surgery trials.

Term used	Number of trials reporting outcome	Follow-up period used	Frequency defined	Definitions provided
'Overall Survival' including 'death from all causes'	19	Not described<br>3 years<br>5 years<br>6 years<br>7 years<br>10 years	7	• Date of randomisation until the day of death or the day of last follow-up (censored).<br>• Date of surgery to the date of death from any cause, censoring the follow-up time at the most recent date for living patients.<br>• Date of randomization to the date of death.<br>• Date of randomization to the date of death from any cause.<br>• OS included operative deaths.<br>• OS excluded post-operative deaths.
'Survival' including 'survival period'	13	Not described<br>5 years<br>11 years	4	• Survival excluding operative mortality<br>• Survival 5 years after curative surgery
'Disease-specific survival'	4	Not described	1	• Proportion of patients who had not died from gastric cancer.

For peer review only - http://bmjopen.bmj.com/site/about/guidelines.xhtml

including 'gastric cancer related deaths'		5 years		
Disease-free survival*	1	Not described	1	• Time from randomization to recurrence or death due to any cause.
Recurrence-free survival*	2	Not described	2	• Time from randomization to either the first recurrence or death from any cause.<br>• Time from randomization to the first documentation of cancer recurrence or death from any cause.

*Although these terms do not relate to mortality, they have been included in this table as the definitions provided by papers describe death as an end-point.

For peer review only

For peer review only - http://bmjopen.bmj.com/site/about/guidelines.xhtml

Table 5. The ten most frequently reported outcomes.

Outcome	Theme	Number of trials reporting outcome	Trials reporting the outcome*
Number of lymph nodes dissected/resected/retrieved	Technical aspects of surgery	22	2, 3, 5, 7, 9, 10, 13, 14, 15, 16, 17, 18, 19, 20, 21, 23, 24, 25, 26, 29, 31, 32
Operative time	Technical aspects of surgery	18	4, 5, 7, 8, 10, 11, 13, 14, 15, 16, 20, 22, 23, 24, 25, 26, 27, 30
Anastomotic leak	Adverse events	17	1, 2, 4, 6, 10, 14, 16, 17, 19, 22, 23, 24, 25, 27, 28, 31, 32
Pancreatic fistula	Adverse events	15	1, 2, 4, 5, 10, 16, 17, 19, 22, 23, 26, 27, 28, 29, 32
Duration of hospital stay	Patient pathway	12	1, 2, 5, 6, 7, 9, 13, 15, 20, 23, 26, 28
Duration of post-operative hospital stay	Recovery from surgery	11	2, 7, 9, 11, 14, 16, 19, 24, 25, 27, 32
Pneumonia	Adverse events	11	7, 10, 11, 12, 17, 23, 27, 28, 29, 31, 32
'5-year' survival	Mortality	11	1, 2, 6, 9, 10, 16, 17, 18, 19, 22, 26
Wound infection	Adverse events	10	2, 6, 12, 15, 16, 18, 19, 22, 24, 28
Abdominal abscess	Adverse events	10	5, 9, 10, 16, 17, 19, 23, 26, 28, 29

*See table 1 for trial numbers and associated publications.

For peer review only - http://bmjopen.bmj.com/site/about/guidelines.xhtml

Discussion

This review is the first to examine the subject of outcome reporting in this field and demonstrates significant inconsistencies in the outcomes measured in trials examining therapeutic surgical interventions for gastric cancer. Not only is there disagreement about 'what' outcomes should be measured, but also 'when' and 'how' they should be measured. Consequently, undertaking meta-analyses and systematic reviews of these interventions becomes problematic and impacts negatively on the ability of researchers and clinicians to formulate robust clinical guidelines for the radical treatment of gastric cancer.

This problem is not confined to previously reported trials. Our analysis of outcome reporting in multi-centre studies and review of active trial protocols has demonstrated similar issues and further highlights the potential 'research waste' within this field. Glasziou and Chambers' estimate that 85 per cent of all biomedical research is 'wasted', and that a significant proportion of this can be attributed to problems choosing and reporting relevant outcomes in trials[5]. Thus, if we combine the number of patients who have participated in gastric cancer surgery trials over the last two decades with those that actively recruiting trials wish to attract, a total of twenty thousand patients may have participated in trials which, from a methodological perspective, could have had a far greater benefit and impact. Not only does this represent an inefficient use of time and scarce financial resource, but it may also have a longer-lasting negative impact on future trial participation by patients. Similar issues related to outcome reporting have been identified by several other groups supporting the theory that this is a widespread problem[6–8,12].

Furthermore, if the methodology of a particular trial is not sufficiently robust or the outcomes reported are not relevant to key stakeholders, the natural course will be for other researchers to examine the same interventions again, using a different approach. If these subsequent trials do not address the underlying methodological issues, they only contribute to a perpetual cycle which serves to weaken the evidence base. This is reflected within the field of gastric cancer surgery where thirteen trials have examined minimally invasive gastrectomy and a further 13 are actively recruiting to trials examining the same intervention.

For peer review only - http://bmjopen.bmj.com/site/about/guidelines.xhtml

Reported outcomes should be relevant to key stakeholders including patients. Given the sheer volume of complications that are reported by gastric surgical trials, one may expect to find the impact on QoL is routinely reported. Indeed, QoL has been demonstrated as an important outcome to measure in other gastrointestinal cancer fields[86]. This has certainly not been the case with gastric cancer surgery trials over the last two decades and whilst there seems to be a greater acceptance by trials currently in recruitment that QoL is important to measure (although this group still represents less than half of ongoing trials), there remains great variation in relation to 'how' and 'when' it is measured.

To address these inconsistencies, we believe that a 'core outcome set' (COS) is required for gastric cancer surgery studies. Developing a minimum reporting standard will contribute to maximising the benefits from randomized control trials which are expensive, labour intensive and logistically challenging to set up. A COS does not aim to restrict the outcomes that are reported, but merely to ensure that the most critical outcomes (as decided by key stakeholders) are clearly defined and measured uniformly.

The challenges associated with inconsistent outcome reporting in trials is certainly not confined to the field of gastric cancer. The COMET (Core Outcome Measurement in Effectiveness Trials) initiative database (http://www.comet-initiative.org/studies/search) contains details of over 400 completed, active or planned COS projects from across many different specialities[87]. Whilst experience within this relatively new research field has grown considerably over the last decade, there is still much work to be done to further develop the various methodological approaches which can be applied. The GASTROS study aims to add to this in several ways including examining the role of 'internationalising' COS development by undertaking a multi-language Delphi survey as part of a consensus-seeking process.

Strengths and Limitations

In addition to being the first systematic review to examine this subject, this study is based on a reproducible and transparent methodology which has been subjected to critical appraisal from a study management team and peer-review process; a protocol of the GASTROS study which aims to develop this COS has been published previously[4]. Nonetheless, there are limitations. Including non-English

For peer review only - http://bmjopen.bmj.com/site/about/guidelines.xhtml

and non-randomized studies in our search strategy may have identified other different outcomes reported in this field. However, when finalising our inclusion criteria for this review, the two primary objectives of this review were considered - namely a) to describe the current landscape of outcome reporting in gastric cancer surgery RCTs and b) to take forward a ‘long-list’ of outcomes to be prioritised (by means of a Delphi survey) to form the basis of a COS for RCTs. Whilst we accept that such a COS would have benefits to non-RCTs and national audits, our primary focus was to improve the quality of RCTs and hence excluding other study types. In addition, there will be an opportunity during the Delphi survey (stage 2 of the GASTROS study) for participants to add further outcomes (not already identified from this review) which key stakeholders deem important to be considered for prioritisation. A further limitation to this review was that it was not prospectively registered on a public database. However, as we describe above, the GASTROS study, including its scope and systematic review plan, has been peer-reviewed and published previously[4].

In summary, the reporting of outcomes in gastric cancer surgery trials is inconsistent and there is large variation with respect to definitions, measurement tools and timing of measurement. This means that data cannot be synthesized efficiently. We believe that a COS to define a minimum set of standards to implement across all gastric surgical trial is warranted.

For peer review only

For peer review only - http://bmjopen.bmj.com/site/about/guidelines.xhtml

Declarations

Ethics Approval and Consent to Participate

Not applicable.

Consent for Publication

Not applicable.

Data Sharing Statement

Upon the completion of the study, the original data file (consisting of extracted data from trial papers) is available upon request from the corresponding author, following discussion and agreements by the study management team.

Competing interests

The authors declare that they have no competing interests.

Funding

This study is funded by the National Institute for Health Research (NIHR) Doctoral Research Fellowship Grant programme (DRF-2015-08-023). The funding body has no role in the study design and implementation, writing the report, or decision to submit the report for publication.

Authors' contributions

BA contributed to the study design and drafted the manuscript. BA and AMG extracted the data from manuscripts. AMG, JMB, PRW and IAB contributed to the study design and revised the manuscript. All authors read and approved the final manuscript.

Authors' Information

Not applicable.

Acknowledgements

The authors would like to thank the patient representatives on the GASTROS Study Advisory Group for their continued advice and invaluable contribution to this study.

For peer review only - http://bmjopen.bmj.com/site/about/guidelines.xhtml

For peer review only

For peer review only - http://bmjopen.bmj.com/site/about/guidelines.xhtml

References

1. Fact Sheets by Cancer. http://globocan.iarc.fr/Pages/fact_sheets_cancer.aspx. Accessed July 24, 2017.

2. Quaresma M, Coleman MP, Rachet B. 40-year trends in an index of survival for all cancers combined and survival adjusted for age and sex for each cancer in England and Wales, 1971–2011: a population-based study. Lancet. 2015;385(9974):1206-1218. doi:10.1016/S0140-6736(14)61396-9.

3. Williamson PR, Altman DG, Bagley H, et al. The COMET Handbook: version 1.0. Trials. 2017;18(S3):280. doi:10.1186/s13063-017-1978-4.

4. Alkhaffaf B, Glenny A-M, Blazeby JM, Williamson P, Bruce IA. Standardising the Reporting of Outcomes in Gastric Cancer Surgery Trials: Protocol for the Development of a Core Outcome Set and Accompanying Outcome Measurement Instrument Set (The GASTROS Study). Trials. 2017;18(1):370. doi:10.1186/s13063-017-2100-7.

5. Chalmers I, Glasziou P. Avoidable waste in the production and reporting of research evidence. Lancet (London, England). 2009;374(9683):86-89. doi:10.1016/S0140-6736(09)60329-9.

6. Hopkins JC, Howes N, Chalmers K, et al. Outcome reporting in bariatric surgery: an in-depth analysis to inform the development of a core outcome set, the BARIACT Study. Obes Rev. 2015;16(1):88-106. doi:10.1111/obr.12240.

7. Blencowe NS, Strong S, McNair AGK, et al. Reporting of short-term clinical outcomes after esophagectomy: a systematic review. Ann Surg. 2012;255(4):658-666. doi:10.1097/SLA.0b013e3182480a6a.

8. Whistance RN, Forsythe RO, McNair AGK, et al. A systematic review of outcome reporting in colorectal cancer surgery. Colorectal Dis. 2013;15(10):e548-60. doi:10.1111/codi.12378.

9. Duffy JMN, Hirsch M, Gale C, et al. A systematic review of primary outcomes and outcome measure reporting in randomized trials evaluating treatments for pre-eclampsia. Int J Gynecol

For peer review only - http://bmjopen.bmj.com/site/about/guidelines.xhtml

Obstet. 2017;139(3):262-267. doi:10.1002/ijgo.12298.

10. McCulloch P. Randomised trials in surgery: problems and possible solutions. BMJ. 2002;324(7351):1448-1451. doi:10.1136/bmj.324.7351.1448.

11. Alkhaffaf B, Glenny A-M, Blazeby JM, Williamson P, Bruce IA. Standardising the reporting of outcomes in gastric cancer surgery trials: Protocol for the development of a core outcome set and accompanying outcome measurement instrument set (the GASTROS study). Trials. 2017;18(1). doi:10.1186/s13063-017-2100-7.

12. Currie A, Brigic A, Blencowe NS, et al. Systematic review of surgical innovation reporting in laparoendoscopic colonic polyp resection. Br J Surg. 2015;102(2):e108-e116. doi:10.1002/bjs.9675.

13. Dodd S, Clarke M, Becker L, Mavergames C, Fish R, Williamson PR. A taxonomy has been developed for outcomes in medical research to help improve knowledge discovery. J Clin Epidemiol. 2018;96:84-92. doi:10.1016/j.jclinepi.2017.12.020.

14. Hasegawa S, Yamamoto Y, Taguri M, et al. A Randomized Phase II Trial of Omentum-preserving Gastrectomy for Advanced Gastric Cancer. doi:10.1093/jjco/hys208.

15. Nakamura K, Katai H, Mizusawa J, et al. A Phase III Study of Laparoscopy-assisted Versus Open Distal Gastrectomy with Nodal Dissection for Clinical Stage IA/IB Gastric Cancer (JCOG0912). Jpn J Clin Oncol. 2013;43(3):324-327. doi:10.1093/jjco/hys220.

16. Yoshikawa T, Fukunaga T, Taguri M, et al. Laparoscopic or Open Distal Gastrectomy After Neoadjuvant Chemotherapy for Operable Gastric Cancer, a Randomized Phase II Trial (LANDSCOPE Trial). Jpn J Clin Oncol. 2012;42(7):654-657. doi:10.1093/jjco/hys057.

17. Effect of Laparoscopy-Assisted Distal Gastrectomy for Locally Advanced Gastric Cancer - Full Text View - ClinicalTrials.gov. https://clinicaltrials.gov/ct2/show/NCT02464215. Accessed July 25, 2017.

18. The Long-term Effect of D4 Lymphadenectomy for Gastric Cancer - Full Text View -

For peer review only - http://bmjopen.bmj.com/site/about/guidelines.xhtml

ClinicalTrials.gov. https://clinicaltrials.gov/ct2/show/NCT02423278. Accessed July 25, 2017.

19. A Comparison of Laparoscopic With Open Distal Gastrectomy in Advanced Gastric Cancer After Neoadjuvant Chemotherapy - Full Text View - ClinicalTrials.gov. https://clinicaltrials.gov/ct2/show/NCT02404753. Accessed July 25, 2017.

20. Randomized Controlled Trials Comparing Clinical Outcomes of 3D Versus 2D Laparoscopic Surgery for Gastric Cancer - Full Text View - ClinicalTrials.gov. https://clinicaltrials.gov/ct2/show/NCT02327481. Accessed July 25, 2017.

21. Prospective Study on Modified Delta-shaped Gastroduodenostomy in Totally Laparoscopic Surgery for Distal Gastric Cancer - Full Text View - ClinicalTrials.gov. https://clinicaltrials.gov/ct2/show/NCT02289183. Accessed July 25, 2017.

22. Haverkamp L, Brenkman HJF, Seesing MFJ, et al. Laparoscopic versus open gastrectomy for gastric cancer, a multicenter prospectively randomized controlled trial (LOGICA-trial). BMC Cancer. 2015;15:556. doi:10.1186/s12885-015-1551-z.

23. Laparoscopy-assisted and Open Distal Gastrectomy for Gastric Cancer in the Elderly Patients. https://clinicaltrials.gov/ct2/show/NCT02246153.

24. A Clinical Trial of D1+ Versus D2 Distal Gastrectomy for Stage IB & II Advanced Gastric Cancer - Full Text View - ClinicalTrials.gov. https://clinicaltrials.gov/ct2/show/NCT02144727. Accessed July 25, 2017.

25. Straatman J, van der Wielen N, Cuesta MA, et al. Surgical techniques, open versus minimally invasive gastrectomy after chemotherapy (STOMACH trial): study protocol for a randomized controlled trial. Trials. 2015;16(1):123. doi:10.1186/s13063-015-0638-9.

26. D2 and D2+ Radical Surgery for the Treatment of Advanced Distal Gastric Cancer - Full Text View - ClinicalTrials.gov. https://clinicaltrials.gov/ct2/show/NCT01836991. Accessed July 25, 2017.

27. Park JY, Kim Y-W, Ryu KW, et al. Assessment of laparoscopic stomach preserving surgery

For peer review only

with sentinel basin dissection versus standard gastrectomy with lymphadenectomy in early gastric cancer–A multicenter randomized phase III clinical trial (SENORITA trial) protocol. BMC Cancer. 2016;16(1):340. doi:10.1186/s12885-016-2336-8.

28. Laparoscopy-assisted Total Gastrectomy for Clinical Stage I Gastric Cancer (KLASS-03). https://clinicaltrials.gov/ct2/show/study/NCT01584336. Accessed July 25, 2017.

29. Efficacy of Laparoscopic Subtotal Gastrectomy With D2 Lymph Node Dissection for Locally Advanced Gastric Cancer (KLASS-02-RCT). https://clinicaltrials.gov/ct2/show/NCT01456598.

30. GCSSG-SPNX: Trial to Evaluate Splenectomy in Total Gastrectomy for Proximal Gastric Carcinoma: JCOG0110 - Full Text View - ClinicalTrials.gov. https://clinicaltrials.gov/ct2/show/NCT00112099. Accessed July 25, 2017.

31. Randomized Controlled Trial to Evaluate Laparoscopic versus Open Surgery for Advanced Gastric Cancer (JLSSG0901: Adv.GC-LAP/OPEN, PII/III). http://apps.who.int/trialsearch/Trial2.aspx?TrialID=JPRN-UMIN000003420. Accessed July 25, 2017.

32. Comparsion of D2 verus D3 surgical procedure outcome in locally advanced gastric cancer patients following perioperative chemotherapy. http://ctri.nic.in/Clinicaltrials/pmaindet2.php?trialid=6992&EncHid=&userName=CTRI/2013/08/003882. Accessed July 25, 2017.

33. Prospective clinical trial on the long-term outcome and survival of vagus (coeliac branch)-preserving gastrectomy for cT1-3 gastric cancer. http://www.chictr.org.cn/showprojen.aspx?proj=4360. Accessed July 25, 2017.

34. Prospective clinical trial on the long-term outcome and survival of pylorus-preserving gastrectomy for early gastric cancer. http://www.chictr.org.cn/showprojen.aspx?proj=4361. Accessed July 25, 2017.

For peer review only - http://bmjopen.bmj.com/site/about/guidelines.xhtml

35. Prospective and open multicenter randomize control study for enlarged lymphadenectomy of gastric cancer. http://www.chictr.org.cn/showprojen.aspx?proj=7870. Accessed July 25, 2017.

36. Open vs minimal invasive surgery for gastric submucosal tumor: a randomized trial on efficacy and safety. http://www.chictr.org.cn/showprojen.aspx?proj=4811. Accessed July 25, 2017.

37. A Multicenter Clinical Trial on Laparoscopic Gastric Cancer Surgery Compared With Open Surgery - Full Text View - ClinicalTrials.gov. https://clinicaltrials.gov/ct2/show/NCT02302794. Accessed July 25, 2017.

38. Hartgrink HH. Extended Lymph Node Dissection for Gastric Cancer: Who May Benefit? Final Results of the Randomized Dutch Gastric Cancer Group Trial. J Clin Oncol. 2004;22(11):2069-2077. doi:10.1200/JCO.2004.08.026.

39. Bonenkamp JJ, Songun I, Welvaart K, et al. Randomised comparison of morbidity after D1 and D2 dissection for gastric cancer in 996 Dutch patients. Lancet. 1995;345(8952):745-748. doi:10.1016/S0140-6736(95)90637-1.

40. Sasako M. Risk factors for surgical treatment in the Dutch Gastric Cancer Trial. Br J Surg. 1997;84(11):1567-1571.

41. Songun I, Putter H, Kranenbarg EM-K, Sasako M, van de Velde CJH. Surgical treatment of gastric cancer: 15-year follow-up results of the randomised nationwide Dutch D1D2 trial. Lancet Oncol. 2010;11(5):439-449. doi:10.1016/S1470-2045(10)70070-X.

42. Cuschieri a, Weeden S, Fielding J, et al. Patient survival after D1 and D2 resections for gastric cancer: long-term results of the MRC randomized surgical trial. Surgical Co-operative Group. Br J Cancer. 1999;79(9-10):1522-1530. doi:10.1016/S0959-8049(01)80998-9.

43. Cuschieri a, Fayers P, Fielding J, et al. Postoperative morbidity and mortality after D1 and D2 resections for gastric cancer: preliminary results of the MRC randomised controlled surgical trial. The Surgical Cooperative Group. Lancet. 1996;347:995-999. doi:10.1016/S0959-

For peer review only - http://bmjopen.bmj.com/site/about/guidelines.xhtml

8049(01)80998-9.

44. Marubini E, Bozzetti F, Miceli R, Bonfanti G, Gennari L. Lymphadenectomy in gastric cancer: prognostic role and therapeutic implications. Eur J Surg Oncol. 2002;28(4):406-412. doi:Clinical Trial Comparative Study Randomized Controlled Trial Research Support, Non-U.S. Gov't.

45. Bozzetti F, Marubini E, Bonfanti G, Miceli R, Piano C, Gennari L. Subtotal versus total gastrectomy for gastric cancer: five-year survival rates in a multicenter randomized Italian trial. Italian Gastrointestinal Tumor Study Group. Ann Surg. 1999;230(2):170-178. doi:10.1097/00000658-199908000-00006.

46. Maeta M, Yamashiro H, Saito H, et al. A prospective pilot study of extended (D3) and superextended para-aortic lymphadenectomy (D4) in patients with T3 or T4 gastric cancer managed by total gastrectomy. Surgery. 1999;125(3):325-331. doi:10.1016/S0039-6060(99)70244-8.

47. Furukawa H, Hiratsuka M, Ishikawa O, et al. Total gastrectomy with dissection of lymph nodes along the splenic artery: a pancreas-preserving method. Ann Surg Oncol. 2000;7(9):669-673.

48. Csendes A, Burdiles P, Rojas J, Braghetto I, Diaz JC, Maluenda F. A prospective randomized study comparing D2 total gastrectomy versus D2 total gastrectomy plus splenectomy in 187 patients with gastric carcinoma. Surgery. 2002;131(4):401-407. doi:10.1067/msy.2002.121891.

49. Kitano S, Shiraishi N, Fujii K, Yasuda K, Inomata M, Adachi Y. A randomized controlled trial comparing open vs laparoscopy-assisted distal gastrectomy for the treatment of early gastric cancer: an interim report. Surgery. 2002;131(1 Suppl):0-11. doi:a120115 [pii].

50. Fujii K, Sonoda K, Izumi K, Shiraishi N, Adachi Y, Kitano S. T lymphocyte subsets and Th1/Th2 balance after laparoscopy-assisted distal gastrectomy. Surg Endosc Other Interv

For peer review only - http://bmjopen.bmj.com/site/about/guidelines.xhtml

Tech. 2003;17(9):1440-1444. doi:10.1007/s00464-002-9149-3.

51. Degiuli M, Sasako M, Calgaro M, et al. Morbidity and mortality after D1 and D2 gastrectomy for cancer: interim analysis of the Italian Gastric Cancer Study Group (IGCSG) randomised surgical trial. Eur J Surg Oncol. 2004;30(3):303-308. doi:10.1016/j.ejso.2003.11.020.

52. Degiuli M, Sasako M, Ponti a. Morbidity and mortality in the italian gastric cancer study group randomized clinical trial of D1 versus D2 resection for gastric cancer. Br J Surg. 2010;97(5):643-649. doi:10.1002/bjs.6936.

53. Degiuli M, Sasako M, Ponti A, et al. Randomized clinical trial comparing survival after D1 or D2 gastrectomy for gastric cancer. Br J Surg. 2014;101(2):23-31. doi:10.1002/bjs.9345.

54. Sasako M, Sano T, Yamamoto S, et al. D2 lymphadenectomy alone or with para-aortic nodal dissection for gastric cancer. N Engl J Med. 2008;359(5):453-462. doi:10.1056/NEJMoa0707035.

55. Takeshi Sano. Gastric Cancer Surgery : Morbidity and Mortality Results From a Prospective Randomized Controlled Trial Comparing D2 and Extended Para-Aortic Lymphadenectomy — Japan Clinical Oncology Group Study 9501. J Clin Oncol. 2004;22(14):2767-2773. doi:10.1200/JCO.2004.10.184.

56. Kodera Y, Sasako M, Yamamoto S, Sano T, Nashimoto a, Kurita a. Identification of risk factors for the development of complications following extended and superextended lymphadenectomies for gastric cancer. Br J Surg. 2005;92(9):1103-1109. doi:10.1002/bjs.4979.

57. Tsujinaka T, Sasako M, Yamamoto S, et al. Influence of overweight on surgical complications for gastric cancer: results from a randomized control trial comparing D2 and extended para-aortic D3 lymphadenectomy (JCOG9501). Ann Surg Oncol. 2007;14(2):355-361. doi:10.1245/s10434-006-9209-3.

58. Shibata C, Shiiba K, Funayama Y, et al. Outcomes after Pylorus-preserving Gastrectomy for

For peer review only

For peer review only - http://bmjopen.bmj.com/site/about/guidelines.xhtml

Early Gastric Cancer: A Prospective Multicenter Trial. World J Surg. 2004;28(9):857-861. doi:10.1007/s00268-004-7369-5.

59. Inaba T, Okinaga K, Fukushima R, et al. Prospective randomized study of two laparotomy incisions for gastrectomy: Midline incision versus transverse incision. Gastric Cancer. 2004;7(3):167-171. doi:10.1007/s10120-004-0291-6.

60. Lee JH, Han HS. A prospective randomized study comparing open vs laparoscopy-assisted distal gastrectomy in early gastric cancer: early results. Surg Endosc. 2005;19(2):168-173. doi:10.1007/s00464-004-8808-y.

61. Hayashi H, Ochiai T, Shimada H, Gunji Y. Prospective randomized study of open versus laparoscopy-assisted distal gastrectomy with extraperigastric lymph node dissection for early gastric cancer. Surg Endosc. 2005;19(9):1172-1176. doi:10.1007/s00464-004-8207-4.

62. Huscher CGSS, Mingoli A, Sgarzini G, et al. Laparoscopic versus open subtotal gastrectomy for distal gastric cancer: five-year results of a randomized prospective trial. Ann Surg. 2005;241(2):232-237. doi:10.1097/01.sla.0000151892.35922.f2.

63. Wu C-W, Hsiung C a, Lo S-S, et al. Nodal dissection for patients with gastric cancer: a randomised controlled trial. Lancet Oncol. 2006;7(4):309-315. doi:10.1016/S1470-2045(06)70623-4.

64. Wu C-W, Chiou J-M, Ko F-S, et al. Quality of life after curative gastrectomy for gastric cancer in a randomised controlled trial. Br J Cancer. 2008;98(1):54-59. doi:10.1038/sj.bjc.6604097.

65. Wu CW, Hsiung C a., Lo SS, Hsieh MC, Shia LT, Whang-Peng J. Randomized clinical trial of morbidity after D1 and D3 surgery for gastric cancer. Br J Surg. 2004;91(3):283-287. doi:10.1002/bjs.4433.

66. Wu C-W, Chang I-S, Lo S, et al. Complications following D3 gastrectomy: post hoc analysis of a randomized trial. World J Surg. 2006;30(1):12-16. doi:10.1007/s00268-005-7951-5.

67. Sasako M, Sano T, Yamamoto S, et al. Left thoracoabdominal approach versus abdominal-

For peer review only - http://bmjopen.bmj.com/site/about/guidelines.xhtml

transhiatal approach for gastric cancer of the cardia or subcardia: a randomised controlled trial. Lancet Oncol. 2006;7(8):644-651. doi:10.1016/S1470-2045(06)70766-5.

68. Kurokawa Y, Sasako M, Sano T, et al. Ten-year follow-up results of a randomized clinical trial comparing left thoracoabdominal and abdominal transhiatal approaches to total gastrectomy for adenocarcinoma of the oesophagogastric junction or gastric cardia. Br J Surg. 2015;102(4):341-348. doi:10.1002/bjs.9764.

69. Yu W, Choi GS, Chung HY. Randomized clinical trial of splenectomy versus splenic preservation in patients with proximal gastric cancer. Br J Surg. 2006;93(5):559-563. doi:10.1002/bjs.5353.

70. Kulig J, Popiela T, Kolodziejczyk P, Sierzega M, Szczepanik A. Standard D2 versus extended D2 (D2+) lymphadenectomy for gastric cancer: an interim safety analysis of a multicenter, randomized, clinical trial. Am J Surg. 2007;193(1):10-15. doi:10.1016/j.amjsurg.2006.04.018.

71. Kim YW, Baik YH, Yun YH, et al. Improved quality of life outcomes after laparoscopy-assisted distal gastrectomy for early gastric cancer: results of a prospective randomized clinical trial. Ann Surg. 2008;248(5):721-727. doi:10.1097/SLA.0b013e318185e62e\r00000658-200811000-00006 [pii].

72. Kim YW, Yoon HM, Yun YH, et al. Long-term outcomes of laparoscopy-assisted distal gastrectomy for early gastric cancer: Result of a randomized controlled trial (COACT 0301). Surg Endosc Other Interv Tech. 2013;27(11):4267-4276. doi:10.1007/s00464-013-3037-x.

73. Yonemura Y, Wu CC, Fukushima N, et al. Randomized clinical trial of D2 and extended paraaortic lymphadenectomy in patients with gastric cancer. Int J Clin Oncol. 2008;13(2):132-137. doi:10.1007/s10147-007-0727-1.

74. Kim W, Kim H-H, Han S-U, et al. Decreased Morbidity of Laparoscopic Distal Gastrectomy Compared With Open Distal Gastrectomy for Stage I Gastric Cancer. Ann Surg. 2015;XX(X):1. doi:10.1097/SLA.0000000000001346.

For peer review only - http://bmjopen.bmj.com/site/about/guidelines.xhtml

75. Kim H-H, Hyung WJ, Cho GS, et al. Morbidity and mortality of laparoscopic gastrectomy versus open gastrectomy for gastric cancer: an interim report--a phase III multicenter, prospective, randomized Trial (KLASS Trial). Ann Surg. 2010;251(3):417-420. doi:10.1097/SLA.0b013e3181cc8f6b.

76. Imamura H, Kurokawa Y, Kawada J, et al. Influence of bursectomy on operative morbidity and mortality after radical gastrectomy for gastric cancer: Results of a randomized controlled trial. World J Surg. 2011;35(3):625-630. doi:10.1007/s00268-010-0914-5.

77. Cai J, Wei D, Gao CF, Zhang CS, Zhang H, Zhao T. A prospective randomized study comparing open versus laparoscopy-assisted D2 radical gastrectomy in advanced gastric cancer. Dig Surg. 2012;28(5-6):331-337. doi:10.1159/000330782.

78. Chen Hu J, Xin Jiang L, Cai L, et al. Preliminary experience of fast-track surgery combined with laparoscopy-assisted radical distal gastrectomy for gastric cancer. J Gastrointest Surg. 2012;16(10):1830-1839. doi:10.1007/s11605-012-1969-4.

79. Takiguchi S, Fujiwara Y, Yamasaki M. Laparoscopy-Assisted Distal Gastrectomy Versus Open Distal. World J Surg. 2013;37(10):2379-2386. doi:10.1007/s00268-013-2121-7.

80. Lee J-H, Son S-Y, Lee CM, Ahn SH, Park DJ, Kim H-H. Morbidity and mortality after laparoscopic gastrectomy for advanced gastric cancer: Results of a phase II clinical trial. Surg Endosc Other Interv Tech. 2013;27(8):2877-2885. doi:10.1007/s00464-013-2848-0.

81. Sakuramoto S, Yamashita K, Kikuchi S, et al. Laparoscopy versus open distal gastrectomy by expert surgeons for early gastric cancer in Japanese patients: short-term clinical outcomes of a randomized clinical trial. Surg Endosc. 2013;27(5):1695-1705. doi:10.1007/s00464-012-2658-9.

82. Aoyama T, Yoshikawa T, Hayashi T, et al. Randomized comparison of surgical stress and the nutritional status between laparoscopy-assisted and open distal gastrectomy for gastric cancer. Ann Surg Oncol. 2014;21(6):1983-1990. doi:10.1245/s10434-014-3509-9.

For peer review only

For peer review only - http://bmjopen.bmj.com/site/about/guidelines.xhtml

83. Hirao M, Kurokawa Y, Fujita J, et al. Long-term outcomes after prophylactic bursectomy in patients with resectable gastric cancer: Final analysis of a multicenter randomized controlled trial. Surgery. 2015;157(6):1099-1105. doi:10.1016/j.surg.2014.12.024.

84. Galizia G, Lieto E, De Vita F, et al. Modified versus standard D2 lymphadenectomy in total gastrectomy for nonjunctional gastric carcinoma with lymph node metastasis. Surg (United States). 2015;157(2):285-296. doi:10.1016/j.surg.2014.09.012.

85. Hu Y, Huang C, Sun Y, et al. Morbidity and Mortality of Laparoscopic Versus Open D2 Distal Gastrectomy for Advanced Gastric Cancer: A Randomized Controlled Trial. J Clin Oncol. 2016. doi:10.1200/JCO.2015.63.7215.

86. Avery KNL, Chalmers KA, Brookes ST, et al. Development of a Core Outcome Set for Clinical Effectiveness Trials in Esophageal Cancer Resection Surgery. Ann Surg. March 2017:1. doi:10.1097/SLA.0000000000002204.

87. Database Search - Core Outcome Measures in Effectiveness Trials Initiative (COMET). http://www.comet-initiative.org/studies/search. Accessed May 19, 2016.

For peer review only

Figure Legends

Figure 1. Study selection and inclusion.

Figure 2. Outcome themes reported in a) gastric cancer surgery trials and b) in future trials based on study protocols.

For peer review only

For peer review only - http://bmjopen.bmj.com/site/about/guidelines.xhtml

Figure 1. Study selection and inclusion.

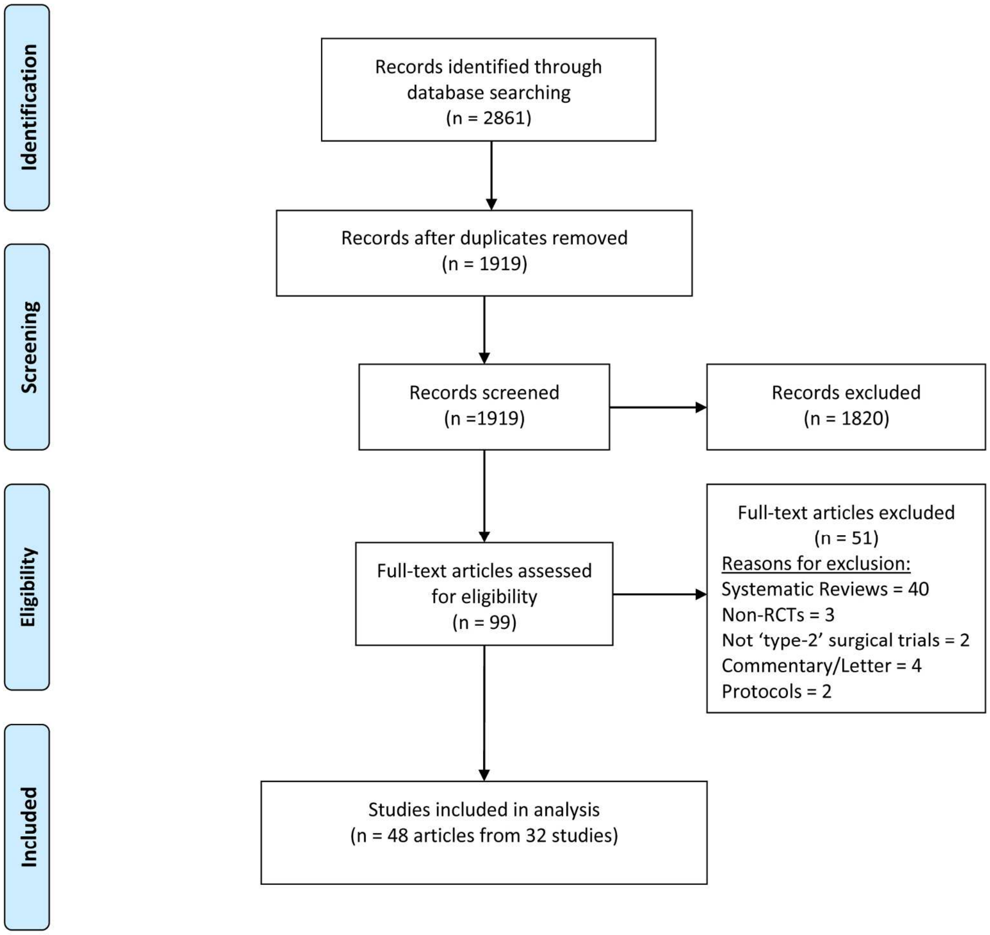

Figure 1. Study selection and inclusion.

185x192mm (300 x 300 DPI)

Figure 2. Outcome themes reported in a) gastric cancer surgery trials and b) in future trials based on study protocols.

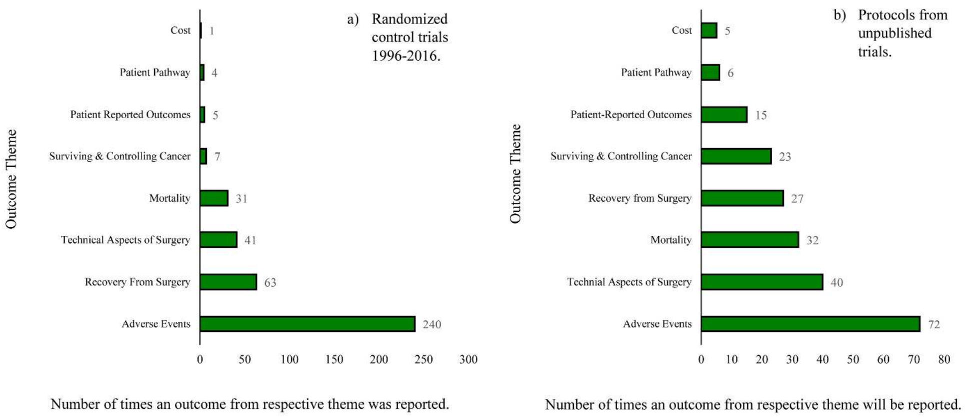

Figure 2. Outcome themes reported in a) gastric cancer surgery trials and b) in future trials based on study protocols.

258x126mm (300 x 300 DPI)

For peer review only

Appendix 1. Search Algorithm

Database: Ovid MEDLINE (R)
Search Strategy:
----
1 (Gastr$ or stomach$ or oesophagogastric junction or esophagogastric junction).mp. (645201)
2 exp Stomach/ (112711)
3 adenocarcinoma/ or neoplasms/ (457904)
4 1 or 2 (648680)
5 3 and 4 (37749)
6 exp Stomach Neoplasms/ (81208)
7 5 or 6 (100682)
8 chemoradiotherapy/ or chemotherapy, adjuvant/ or consolidation chemotherapy/ or antineoplastic combined chemotherapy protocols/ or induction chemotherapy/ (140865)
9 (antineoplast$ or antitumor$ or anti-tumor$ or anti-neoplast$ or chemotherp$).mp. (454727)
10 exp Gastrectomy/ or endoscopy, gastrointestinal/ or duodenoscopy/ or esophagoscopy/ or gastroscopy/ (69973)
11 (gastrectom$ or duodenoscop$ or esophagoscop$ or oesophagoscop$ or gastroscop$).mp. [mp=title, abstract, original title, name of substance word, subject heading word, keyword heading word, protocol supplementary concept word, rare disease supplementary concept word, unique identifier] (66259)
12 radiotherapy/ or brachytherapy/ or radioimmunotherapy/ or radiotherapy, adjuvant/ (74386)
13 (radiotherap$ or chemoradiotherap$ or chemo-radiotherap$ or "radiation therap$" or bracytherap$ or irradiat$).mp. (366760)
14 8 or 9 or 10 or 12 or 13 (854226)
15 7 and 14 (32194)
16 randomi?ed controlled trial.pt. (416592)
17 controlled clinical trial.pt. (92207)
18 randomized.ab. (308489)
19 randomly.ab. (218498)
20 exp animals/ not humans.sh. (4150916)
21 16 or 17 or 18 or 19 (743790)
22 21 not 20 (673762)
23 15 and 22 (2255)
24 limit 23 to yr="1996 -Current" (1806)
25 (Gastr$ or stomach$ or oesophagogastric junction or esophagogastric junction).mp. (645201)
26 exp Stomach/ (112711)
27 adenocarcinoma/ or neoplasms/ (457904)
28 25 or 26 (648680)
29 27 and 28 (37749)
30 exp Stomach Neoplasms/ (81208)
31 29 or 30 (100682)
32 chemoradiotherapy/ or chemotherapy, adjuvant/ or consolidation chemotherapy/ or antineoplastic combined chemotherapy protocols/ or induction chemotherapy/ (140865)
33 (antineoplast$ or antitumor$ or anti-tumor$ or anti-neoplast$ or chemotherp$).mp. (454727)
34 exp Gastrectomy/ or endoscopy, gastrointestinal/ or duodenoscopy/ or esophagoscopy/ or gastroscopy/ (69973)
35 (gastrectom$ or duodenoscop$ or esophagoscop$ or oesophagoscop$ or gastroscop$).mp. [mp=title, abstract, original title, name of substance word, subject heading word, keyword heading word, protocol supplementary concept word, rare disease supplementary concept word, unique identifier] (66259)
36 radiotherapy/ or brachytherapy/ or radioimmunotherapy/ or radiotherapy, adjuvant/ (74386)
37 (radiotherap$ or chemoradiotherap$ or chemo-radiotherap$ or "radiation therap$" or bracytherap$ or irradiat$).mp. (366760)
38 32 or 33 or 34 or 36 or 37 (854226)
39 31 and 38 (32194)
40 randomi?ed controlled trial.pt. (416592)
41 controlled clinical trial.pt. (92207)

For peer review only

42 randomized.ab. (308489)
43 randomly.ab. (218498)
44 exp animals/ not humans.sh. (4150916)
45 40 or 41 or 42 or 43 (743790)
46 45 not 44 (673762)
47 39 and 46 (2255)
48 limit 47 to yr="1996 -Current" (1806)

For peer review only

Appendix 2. Outcomes reported in 48 publications from 32 gastric cancer surgery trials.

Theme	Original Outcome	Frequency Reported
Adverse Events	Abdominal abscess	10
Adverse Events	Abdominal distention	1
Adverse Events	Abdominal drainage	1
Adverse Events	Abdominal liquid accumulation	1
Adverse Events	Abscess intra-abdominal	7
Adverse Events	Abscess subphrenic	1
Adverse Events	Acute enteritis	1
Adverse Events	Acute urinary retention	1
Adverse Events	Adverse drug reaction	1
Adverse Events	Afferent loop syndrome	2
Adverse Events	Allogenic blood transfusion	1
Adverse Events	Amylase level in drainage fluid	1
Adverse Events	Anastomosis failure	1
Adverse Events	Anastomosis stricture	2
Adverse Events	Anastomotic bleeding	1
Adverse Events	Anastomotic dehiscence	2
Adverse Events	Anastomotic leak	17
Adverse Events	Anastomotic leakage from GJ	1
Adverse Events	Anastomotic leakage from OJ	1
Adverse Events	Anastomotic leakage type 1	1
Adverse Events	Anastomotic leakage type 2	1
Adverse Events	Anastomotic stenosis	5
Adverse Events	Any complication	2
Adverse Events	ARDS	1

For peer review only

Adverse Events	Arteriosclerosis obliterans of the leg	1
Adverse Events	Ascites	2
Adverse Events	Atelectasis	2
Adverse Events	Atelectasis or pleural effusion	1
Adverse Events	Atrial fibrillation	1
Adverse Events	Bleeding	4
Adverse Events	Bleeding abdominal	1
Adverse Events	Bleeding from anastomosis	1
Adverse Events	Blood transfusion	2
Adverse Events	Blood transfusion volume	2
Adverse Events	Body temperature exceeding 37 ° C (days)	2
Adverse Events	Bowel obstruction	1
Adverse Events	Bowel obstruction/ileus	1
Adverse Events	Bronchopneumonia	1
Adverse Events	Bronchoscopic toilet	1
Adverse Events	Cardiac complications	6
Adverse Events	Cardiac failure	1
Adverse Events	Cardiocirculatory	1
Adverse Events	Cardiopulmonary disaese	1
Adverse Events	Catheter-induced sepsis	1
Adverse Events	Cerebrovascular	1
Adverse Events	Cholecystitis	3
Adverse Events	Cholecystitis acute	1
Adverse Events	Cholecystitis requiring percutaneous drainage	1
Adverse Events	Chyle leakage	2
Adverse Events	Chylous drainage	1
Adverse Events	Chylous lymphorrhea	1

For peer review only

Adverse Events	Colonic perforation	1
Adverse Events	Complications	3
Adverse Events	Complications after discharge	1
Adverse Events	Complications number of	1
Adverse Events	Deep vein thrombosis	3
Adverse Events	Delayed gastric emptying	2
Adverse Events	Delayed gastric emptying without obstruction	1
Adverse Events	Diarrhoea	1
Adverse Events	Drug-induced hepatitis	1
Adverse Events	Dumping syndrome	1
Adverse Events	Duodenal leak	1
Adverse Events	Duodenal stump leak	3
Adverse Events	Early dumping syndrome	1
Adverse Events	Early surgical complications	1
Adverse Events	Empyema thoracis	1
Adverse Events	Endocrine complications	1
Adverse Events	Endocrine events	1
Adverse Events	Enterocutaneous fistula	3
Adverse Events	Esophagus and remnant stomach infarction	1
Adverse Events	Fever	1
Adverse Events	Fluid collection	1
Adverse Events	Fluid collection/abscesses	1
Adverse Events	Gastric atonia	1
Adverse Events	Gastric remnant necrosis	1
Adverse Events	Gastrointestinal bleeding	1
Adverse Events	Gastrointestinal complications	1
Adverse Events	Gastrointestinal injury	1

For peer review only

Adverse Events	Gastroparesis	3
Adverse Events	Haemorrhage	2
Adverse Events	Hb	2
Adverse Events	Hepatic complications	2
Adverse Events	Hepatic failure	1
Adverse Events	Herpes zoster	1
Adverse Events	Hiccups	1
Adverse Events	Hospital morbidity	1
Adverse Events	Hypercapnia	1
Adverse Events	Iatrogenic spleen injury	1
Adverse Events	Idiopathic small bowel perforation	1
Adverse Events	Iileus mechanical	1
Adverse Events	Ileus	2
Adverse Events	Ileus adhesive	2
Adverse Events	Ileus paralytic	1
Adverse Events	Ileus prolonged	1
Adverse Events	Incision fat liquefaction	1
Adverse Events	Incision infection	1
Adverse Events	Infection	1
Adverse Events	Intestinal fistula	2
Adverse Events	Intestinal ischaemia	1
Adverse Events	Intestinal obstruction	3
Adverse Events	Intra-abdominal bleeding	3
Adverse Events	Intra-abdominal collections	1
Adverse Events	Intra-abdominal complications	1
Adverse Events	Intra-abdominal infection	1
Adverse Events	Intraluminal bleeding	3

For peer review only

Adverse Events	Intraoperative blood transfusion	2
Adverse Events	Intraoperative complications	3
Adverse Events	intraoperative major bleeding	1
Adverse Events	Intraperitoneal haemorrhage	1
Adverse Events	Late surgical complications	1
Adverse Events	Leakage	1
Adverse Events	Liver dysfunction	1
Adverse Events	Local complications	1
Adverse Events	Long-term complications	1
Adverse Events	Lung Infection	1
Adverse Events	Lymphatic leakage	1
Adverse Events	Lymphorrhoea	1
Adverse Events	Major abdominal infections	1
Adverse Events	Major cardiorespiratory incidents	1
Adverse Events	Major complications	2
Adverse Events	Major post-operative complication	1
Adverse Events	Major surgical complications	1
Adverse Events	Malabsorption	1
Adverse Events	Mediastinitis	1
Adverse Events	Medical complications	1
Adverse Events	Metabolic complications	1
Adverse Events	Minor complications	1
Adverse Events	Minor discharge of pancreatic juice	1
Adverse Events	Minor leakage	1
Adverse Events	Minor patchy pulmonary collapse	1
Adverse Events	Minor pulmonary atelectasis	2
Adverse Events	Morbidity	8

For peer review only

Adverse Events	Morbidity rate	1
Adverse Events	Multiple organ failure	1
Adverse Events	Myocardial infarction	1
Adverse Events	Nausea	1
Adverse Events	Need for blood transfusion	1
Adverse Events	Non-surgical Complications	6
Adverse Events	Number of patients with complications	1
Adverse Events	Operative complications	1
Adverse Events	Operative morbidity	3
Adverse Events	Other complications	7
Adverse Events	Overall complications	2
Adverse Events	Overall Post-operative complications	1
Adverse Events	Pancreas-related complications	1
Adverse Events	Pancreatic fistula	15
Adverse Events	Pancreatic injury	2
Adverse Events	Pancreatic leak	4
Adverse Events	Pancreatitis	1
Adverse Events	Pancreatitis acute	3
Adverse Events	Pancreatitis edematous	1
Adverse Events	Pancreatitis severe	1
Adverse Events	Pancreatitis traumatic	1
Adverse Events	Peri-operative complications	1
Adverse Events	Peritoneal haemorrhage	1
Adverse Events	Pleural	1
Adverse Events	Pleural effusion	5
Adverse Events	Pleural fluid	1
Adverse Events	Pneumonia	11

For peer review only

Adverse Events	Post-operative bleeding	1
Adverse Events	Post-operative complications	5
Adverse Events	Post-operative drain discharge	1
Adverse Events	Post-operative glucose tolerance	1
Adverse Events	Post-operative hemorrhage	2
Adverse Events	Post-operative major complications	1
Adverse Events	Post-operative minor complication	1
Adverse Events	Post-operative morbidity	1
Adverse Events	Post-operative psychosis	1
Adverse Events	Post-operative respiratory care	1
Adverse Events	Post-operative respiratory function	1
Adverse Events	Post-operative surgical parameters	1
Adverse Events	Post-operative symptoms	1
Adverse Events	Presence of gallstones	1
Adverse Events	Procedure-related morbidity and mortality	1
Adverse Events	Prolonged diarrhea	1
Adverse Events	Prolonged retention of intra-abdominal fluid	1
Adverse Events	Pulmonary	4
Adverse Events	Pulmonary complications	4
Adverse Events	Pulmonary edema	1
Adverse Events	Pulmonary embolism	2
Adverse Events	Pulmonary infection	1
Adverse Events	Pyothorax	1
Adverse Events	Rate of reinsertion of NG tube	1
Adverse Events	Recurrent laryngeal nerve palsy	1
Adverse Events	Reflux oesophagitis	2
Adverse Events	Re-laparotomy	2

For peer review only

Adverse Events	Renal complications	2
Adverse Events	Renal failure	2
Adverse Events	Re-operation	8
Adverse Events	Re-operation details	1
Adverse Events	Respirator use after surgery	1
Adverse Events	Respiratory complications	1
Adverse Events	Respiratory failure	1
Adverse Events	Return to theatre	1
Adverse Events	Septic complications	1
Adverse Events	Serious and potentially fatal complications	1
Adverse Events	Severe diarrhoea	1
Adverse Events	Severe feeding problem requiring prolonged hyperalimentation	1
Adverse Events	Severity of complications	2
Adverse Events	Severity of post-operative complications	1
Adverse Events	Short-term complications	1
Adverse Events	Small-bowel obstruction	1
Adverse Events	Splenic artery pseudoaneurysm	1
Adverse Events	Splenic injury	1
Adverse Events	Stasis	1
Adverse Events	Stenosis	1
Adverse Events	Surgical complications	8
Adverse Events	Surgical risk	1
Adverse Events	Systemic complications	1
Adverse Events	Systemic infections	1
Adverse Events	Thermal injury	1
Adverse Events	Thoracic effusion requiring thoracic drainage	1

For peer review only

Adverse Events	Thromboembolic complications	1
Adverse Events	Total complications	1
Adverse Events	Total major complications	1
Adverse Events	Total morbidity	4
Adverse Events	Tracheotomy	1
Adverse Events	Transfusion	3
Adverse Events	Transfusions received	1
Adverse Events	Transient ischemic attack	1
Adverse Events	Transient LFT abnormality	1
Adverse Events	Trocar related injury	1
Adverse Events	Tube tracheotomy	1
Adverse Events	Uncomplicated calf vein thrombosis	1
Adverse Events	Upper gastro-intestinal haemorrhage	1
Adverse Events	Urinary complications	2
Adverse Events	Urinary retention	1
Adverse Events	Urinary tract complications	1
Adverse Events	Urinary tract infection	3
Adverse Events	Viral infection	1
Adverse Events	Vomiting	1
Adverse Events	Wound abscess	1
Adverse Events	Wound complications	2
Adverse Events	Wound dehiscence	2
Adverse Events	Wound evisceration	1
Adverse Events	Wound haematoma	1
Adverse Events	Wound infection	10
Adverse Events	Wound infection/dehiscence	1
Adverse Events	Wound problem	1

For peer review only

Adverse Events	Wound seroma	2
Cost	Medical cost	1
Mortality	Death	8
Mortality	Death from a post-operative complication	1
Mortality	Death from all causes	1
Mortality	Death from gastric cancer as a cause	1
Mortality	Disease free survival	4
Mortality	Disease free survival 4-year	2
Mortality	Disease free survival 5-year	3
Mortality	Disease specific survival	1
Mortality	Disease specific survival 5-year	1
Mortality	Gastric cancer related deaths	1
Mortality	Hospital death	3
Mortality	Hospital mortality	7
Mortality	In-hospital mortality	2
Mortality	Mortality not related to surgery	1
Mortality	Operative death	1
Mortality	Operative mortality	3
Mortality	Overall survival	8
Mortality	Overall survival 10-year	1
Mortality	Overall survival 3-year	2
Mortality	Overall survival 5-year	6
Mortality	Overall survival 6-year	1
Mortality	Overall survival 7-year	1
Mortality	Post-operative death	4
Mortality	Post-operative mortality	3
Mortality	Post-operative survival	1

For peer review only

Mortality	Recurrence-free survival	5
Mortality	Relapse-free survival	1
Mortality	Survival 11-year	1
Mortality	Survival 5-year	11
Mortality	Survival Period	1
Mortality	Treatment related deaths	1
Patient Pathway	Days of hospitalization	1
Patient Pathway	Duration of hospital stay	12
Patient Pathway	Duration of post-operative hospital stay	11
Patient Pathway	Readmission	1
Patient Reported Outcomes	Degree of pain	1
Patient Reported Outcomes	Overall satisfaction	1
Patient Reported Outcomes	Pain	1
Patient Reported Outcomes	Post-operative Pain	1
Patient Reported Outcomes	QoL	3
Recovery From Surgery	4-day post-operative use of analgesics	1
Recovery From Surgery	Blood urea nitrogen	1
Recovery From Surgery	Body weight	1
Recovery From Surgery	CK	1
Recovery From Surgery	CRP	4
Recovery From Surgery	CRP 3 days after surgery	1
Recovery From Surgery	Days of fever	1
Recovery From Surgery	Days to sips of water	1
Recovery From Surgery	Decrease in body weight	1
Recovery From Surgery	Decrease of body weight 1 month after the operation	1
Recovery From Surgery	Dose of analgesic (mg)	1
Recovery From Surgery	Duration of pain control	1

For peer review only

Recovery From Surgery	Eating	1
Recovery From Surgery	FEV1(L)	1
Recovery From Surgery	FEVC(L)	1
Recovery From Surgery	Fever	1
Recovery From Surgery	First eating (post-operative day)	1
Recovery From Surgery	First walking (post-operative day)	1
Recovery From Surgery	Food intake	1
Recovery From Surgery	Frequency of analgesics injection	1
Recovery From Surgery	Frequency of injection given according to analgesic requests	1
Recovery From Surgery	IL-6	1
Recovery From Surgery	Immediate postoperative inflammatory and immune responses	1
Recovery From Surgery	Immunological response to surgery	2
Recovery From Surgery	Lean body mass	1
Recovery From Surgery	LFT	1
Recovery From Surgery	Number of days to get out of bed	1
Recovery From Surgery	Nutritional Status	1
Recovery From Surgery	Pain control	2
Recovery From Surgery	Post-operative analgesia	1
Recovery From Surgery	Post-operative course	1
Recovery From Surgery	Post-operative pain	1
Recovery From Surgery	Post-operative recovery	1
Recovery From Surgery	Prealbumin	1
Recovery From Surgery	Progression of oral intake	1
Recovery From Surgery	Pulmonary function	1
Recovery From Surgery	Recovery of Physical Activity	1
Recovery From Surgery	Residual pain at day 7	1

For peer review only

Recovery From Surgery	SaO2	1
Recovery From Surgery	Serum Albumin	1
Recovery From Surgery	Surgical stress response	1
Recovery From Surgery	The early recovery course	1
Recovery From Surgery	Time of first flatus/index of peristalsis recovery	1
Recovery From Surgery	Time to ambulation	1
Recovery From Surgery	Time to first flatus	4
Recovery From Surgery	Time to first flatus (days)	5
Recovery From Surgery	Time to first liquid intake	1
Recovery From Surgery	Time to first soft diet uptake	1
Recovery From Surgery	Time to food intake	1
Recovery From Surgery	Time to liquid diet	1
Recovery From Surgery	Time to removal of epidural anesthesia (days)	1
Recovery From Surgery	Time to sips of water	2
Recovery From Surgery	Time to start oral intake (days)	1
Recovery From Surgery	Time until removal of the naso-gastric tube	1
Recovery From Surgery	Time until start of meals	1
Recovery From Surgery	Times analgesic given	1
Recovery From Surgery	Times of pain rescue	1
Recovery From Surgery	Total amount of analgesics infused	1
Recovery From Surgery	Total body weight	1
Recovery From Surgery	Total protein	1
Recovery From Surgery	Walking	1
Recovery From Surgery	WBC	1
Recovery From Surgery	WCC	3
Surviving & Controlling Cancer	Cumulative risk of recurrence	2

For peer review only

Surviving & Controlling Cancer	Disease recurrence rate	1
Surviving & Controlling Cancer	Port site metastasis	2
Surviving & Controlling Cancer	Recurrence	4
Surviving & Controlling Cancer	Recurrence patterns	1
Surviving & Controlling Cancer	Recurrent disease	1
Surviving & Controlling Cancer	Tumor recurrence	1
Technical Aspects of Surgery	Amount of bleeding	1
Technical Aspects of Surgery	Blood loss	7
Technical Aspects of Surgery	Clear margin distance	1
Technical Aspects of Surgery	Conversion to open surgery	3
Technical Aspects of Surgery	Dissected Lymph nodes - mediastinal	1
Technical Aspects of Surgery	Dissected Lymph nodes - para-aortic	1
Technical Aspects of Surgery	Distal resection margin	2
Technical Aspects of Surgery	Duration of surgery	3
Technical Aspects of Surgery	Estimated blood loss	5
Technical Aspects of Surgery	Intraoperative blood loss	7
Technical Aspects of Surgery	Length of laparotomy incision	1
Technical Aspects of Surgery	Length of lesser curvature of resected stomach	1
Technical Aspects of Surgery	Length of lesser curvature of resected stomach	1
Technical Aspects of Surgery	Length of longest wound	1
Technical Aspects of Surgery	Length of resection on greater cureve	1

For peer review only

Technical Aspects of Surgery	Length of resection on lesser curve	1
Technical Aspects of Surgery	Main wound size	1
Technical Aspects of Surgery	Mean blood loss	2
Technical Aspects of Surgery	Mean operating time	1
Technical Aspects of Surgery	Number of lymph nodes dissected or resected or retrieved	22
Technical Aspects of Surgery	Number of lymph nodes removed N1 group	1
Technical Aspects of Surgery	Number of lymph nodes removed N2 group	1
Technical Aspects of Surgery	Number of lymph nodes removed N3 group	1
Technical Aspects of Surgery	Number of lymph nodes removed N4 group	1
Technical Aspects of Surgery	Operative blood loss	2
Technical Aspects of Surgery	Operative time	18
Technical Aspects of Surgery	Pathological outcomes	1
Technical Aspects of Surgery	Proximal margin positive/negative	1
Technical Aspects of Surgery	Proximal resection margin	2
Technical Aspects of Surgery	R0 resection	2
Technical Aspects of Surgery	Radicality R0	1
Technical Aspects of Surgery	Radicality R1	1
Technical Aspects of Surgery	Resection line involvement - distal	1
Technical Aspects of Surgery	Resection line involvement - proximal	1
Technical Aspects of Surgery	Residual Tumour	1
Technical Aspects of Surgery	Residual tumour R0	2
Technical Aspects of Surgery	Residual tumour R1/2	1
Technical Aspects of Surgery	Residual tumour R1/2	1
Technical Aspects of Surgery	Surgical time	2
Technical Aspects of Surgery	Time for operation	1
Technical Aspects of Surgery	Wound size	5

For peer review only

PRISMA 2009 Checklist

Section/topic	#	Checklist item	Reported on page #
**TITLE**			
Title	1	Identify the report as a systematic review, meta-analysis, or both.	1
**ABSTRACT**			
Structured summary	2	Provide a structured summary including, as applicable: background; objectives; data sources; study eligibility criteria, participants, and interventions; study appraisal and synthesis methods; results; limitations; conclusions and implications of key findings; systematic review registration number.	3
**INTRODUCTION**			
Rationale	3	Describe the rationale for the review in the context of what is already known.	6
Objectives	4	Provide an explicit statement of questions being addressed with reference to participants, interventions, comparisons, outcomes, and study design (PICOS).	7
**METHODS**			
Protocol and registration	5	Indicate if a review protocol exists, if and where it can be accessed (e.g., Web address), and, if available, provide registration information including registration number.	6
Eligibility criteria	6	Specify study characteristics (e.g., PICOS, length of follow-up) and report characteristics (e.g., years considered, language, publication status) used as criteria for eligibility, giving rationale.	8, table 1
Information sources	7	Describe all information sources (e.g., databases with dates of coverage, contact with study authors to identify additional studies) in the search and date last searched.	8,9
Search	8	Present full electronic search strategy for at least one database, including any limits used, such that it could be repeated.	App 1
Study selection	9	State the process for selecting studies (i.e., screening, eligibility, included in systematic review, and, if applicable, included in the meta-analysis).	9
Data collection process	10	Describe method of data extraction from reports (e.g., piloted forms, independently, in duplicate) and any processes for obtaining and confirming data from investigators.	10
Data items	11	List and define all variables for which data were sought (e.g., PICOS, funding sources) and any assumptions and simplifications made.	10
Risk of bias in individual studies	12	Describe methods used for assessing risk of bias of individual studies (including specification of whether this was done at the study or outcome level), and how this information is to be used in any data synthesis.	n/a
Summary measures	13	State the principal summary measures (e.g., risk ratio, difference in means).	n/a
Synthesis of results	14	Describe the methods of handling data and combining results of studies, if done, including measures of consistency (e.g., I^2) for each meta-analysis.	n/a

For peer review only - http://bmjopen.bmj.com/site/about/guidelines.xhtml

PRISMA 2009 Checklist

Section/topic	#	Checklist item	Reported on page #
Risk of bias across studies	15	Specify any assessment of risk of bias that may affect the cumulative evidence (e.g., publication bias, selective reporting within studies).	n/a
Additional analyses	16	Describe methods of additional analyses (e.g., sensitivity or subgroup analyses, meta-regression), if done, indicating which were pre-specified.	n/a
**RESULTS**			
Study selection	17	Give numbers of studies screened, assessed for eligibility, and included in the review, with reasons for exclusions at each stage, ideally with a flow diagram.	Figure 1
Study characteristics	18	For each study, present characteristics for which data were extracted (e.g., study size, PICOS, follow-up period) and provide the citations.	Table 2
Risk of bias within studies	19	Present data on risk of bias of each study and, if available, any outcome level assessment (see item 12).	n/a
Results of individual studies	20	For all outcomes considered (benefits or harms), present, for each study: (a) simple summary data for each intervention group (b) effect estimates and confidence intervals, ideally with a forest plot.	n/a
Synthesis of results	21	Present results of each meta-analysis done, including confidence intervals and measures of consistency.	n/a
Risk of bias across studies	22	Present results of any assessment of risk of bias across studies (see Item 15).	n/a
Additional analysis	23	Give results of additional analyses, if done (e.g., sensitivity or subgroup analyses, meta-regression [see Item 16]).	n/a
**DISCUSSION**			
Summary of evidence	24	Summarize the main findings including the strength of evidence for each main outcome; consider their relevance to key groups (e.g., healthcare providers, users, and policy makers).	30
Limitations	25	Discuss limitations at study and outcome level (e.g., risk of bias), and at review-level (e.g., incomplete retrieval of identified research, reporting bias).	31
Conclusions	26	Provide a general interpretation of the results in the context of other evidence, and implications for future research.	32
**FUNDING**			
Funding	27	Describe sources of funding for the systematic review and other support (e.g., supply of data); role of funders for the systematic review.	2

From: Moher D, Liberati A, Tetzlaff J, Altman DG, The PRISMA Group (2009). Preferred Reporting Items for Systematic Reviews and Meta-Analyses: The PRISMA Statement. PLoS Med 6(7): e1000097. doi:10.1371/journal.pmed1000097

For more information, visit: **www.prisma-statement.org**.

For peer review only - http://bmjopen.bmj.com/site/about/guidelines.xhtml
